# *APOE* genotypes differentially remodel the astrocytic lipid droplet proteome to shape lipid droplet dynamics

**DOI:** 10.1038/s41467-026-76565-6

**Published:** 2026-08-19

**Authors:** Carla Cuní-López, Jessica T. Root, Ying Hao, Niek Blomberg, Sascha J. Koppes-den Hertog, Dylan Sacks, Amber E. Trujillo, Rodolfo Ghirlando, Isabelle Kowal, Jacob Epstein, Linda G. Yang, Mark R. Cookson, Elise Marsan, Rik van der Kant, Martin Giera, Yue Andy Qi, Priyanka S. Narayan

**Affiliations:** 1 Genetics and Biochemistry Branch, National Institute of Diabetes and Digestive and Kidney Diseases, National Institutes of Health, Bethesda, MD USA; 2 Center for Alzheimer’s and Related Dementias (CARD), National Institute on Aging, National Institutes of Health, Bethesda, MD USA; 3 Leiden University Medical Center, Center for Proteomics and Metabolomics, Leiden, the Netherlands; 4 Center for Neurogenomics and Cognitive Research, Vrije Universiteit Amsterdam, Amsterdam Neuroscience, Amsterdam, the Netherlands; 5 Alzheimer Center Amsterdam, Department of Neurology, Amsterdam University Medical Center, Amsterdam Neuroscience, Amsterdam, the Netherlands; 6 DataTecnica, Washington, DC USA; 7 Laboratory of Molecular Biology, National Institute of Diabetes, Digestive and Kidney Diseases, National Institutes of Health, Bethesda, MD USA; 8 Laboratory of Neurogenetics, National Institute on Aging, National Institutes of Health, Bethesda, MD USA

**Keywords:** Organelles, Lipids, Mechanisms of disease

## Abstract

Lipid droplets are dynamic cellular organelles that store neutral lipids and coordinate metabolic and stress-response pathways. In the brain, lipid droplets in glial cells, including astrocytes, have been implicated in Alzheimer’s disease, but how genetic risk factors influence their composition and turnover remains poorly understood. *APOE* is the strongest genetic modulator of late-onset Alzheimer’s disease and exists in common variants that confer decreased, neutral, or increased risk. Here we show that *APOE* genotype shapes the lipid droplet proteome, lipidome, and degradation dynamics in human induced pluripotent stem cell-derived astrocytes. By comparing oleic acid-treated astrocytes carrying *APOE2*, *APOE3*, or *APOE4*, we find that each variant is associated with distinct lipid droplet proteins and lipids. These molecular differences correspond to genotype-dependent changes in lipophagy, an autophagy-mediated pathway for lipid droplet clearance. Lipid droplets in *APOE2* astrocytes undergo efficient autophagic turnover, whereas those in *APOE4* astrocytes resist degradation. These findings identify impaired lipid droplet clearance as a potential mechanism linking *APOE4* to Alzheimer’s disease risk.

## Abstract (web summary)

Alzheimer’s-linked *APOE* variants affect the composition and plasticity of astrocytic lipid droplets. These fat storage organelles are rapidly turned over with protective *APOE2*, but clear slowly with Alzheimer's-associated *APOE4*.

## Introduction

Lipid droplets are dynamic and understudied organelles composed of a neutral lipid core surrounded by a phospholipid monolayer functionalized with many proteins^1^. Traditionally associated with lipid storage, lipid droplets have roles in several other cellular processes, including transcriptional regulation, inflammation, and stress responses^2^. These functions are mediated not only by the lipid content of these organelles but also by the proteins decorating their membranes. Importantly, both cell type and cellular environment modulate the lipid droplet proteome and lipidome^3–9^.

Lipid droplets have been studied extensively in the context of hepatic and adipose tissue, however, these organelles also play key roles in brain cell types. Altered lipid droplet biology has been linked to both brain aging and neurodegenerative diseases^10–24^. Notably, in the first case report of Alzheimer’s disease (AD), Dr. Alzheimer described glial lipid accumulation as one of the disease’s five pathological hallmarks^25^.

Microglial lipid droplets have emerged as an area of key interest in the AD field and in many model systems. Recent studies have identified microglia with lipid droplets in aged mice^18^, AD mouse models^26^, and human iPSC-derived microglia with the *APOE4* genotype^10,13,17,24^. Microglia from *APOE4* mouse models, as well as human microglia from post-mortem brain tissue in AD and *APOE4* carriers, exhibit lipid dysregulation and upregulation of neutral lipid storage^17,27–29^. Microglial lipid droplets have been associated with higher levels of immune activation, altered cell non-autonomous signaling, and cell non-autonomous protein aggregate accumulation^10,13,17,18^.

Astrocytic lipid droplets also accumulate in an *APOE* genotype-dependent manner. *APOE4* astrocytes accumulate unsaturated triglycerides in lipid droplets in both mouse^15,16^ and human iPSC-derived astrocytes^24,30^. These lipid droplets impact antigen presentation on astrocytes^30^ as well as cell non-autonomous effects on neuronal oxidative stress^20,21^. Interestingly, lipid droplets have now been associated with AD risk genes beyond *APOE* (*PICALM* and *INPP5D* in microglia and *ABCA7* in neurons), suggesting convergent biology across risk genes^11,31,32^.

Genetics also underscores the connection between lipid metabolism and AD. Several lipid metabolism genes have been identified as modifiers of AD risk and progression (such as *APOE, PICALM, ABCA7, INPP5D, CLU, TREM2, ABCA1, MS4A4A* and *PLCG2*)^33,34^. Among these, coding variants of the *APOE* gene stand out as the strongest genetic risk and resilience factors for late-onset AD. The *APOE3* allele is the most common and regarded as neutral with respect to AD risk, while single amino acid coding variants result in the *APOE4* allele (C112R), which increases AD risk, and the *APOE2* allele (R158C), which decreases AD risk^12^. Astrocytes produce the most APOE in the brain^35^, and studies in various model systems have linked *APOE4* with increased astrocytic lipid droplets^15,16,20,24^. Microglia, especially those in an immune-reactive state, increase their expression of *APOE*. These microglia have been associated with AD pathology in mouse models^29,36^ and human systems^10,27^.

With the rising importance of lipid droplets in AD, we aimed to systematically characterize the proteome and lipidome of lipid droplets in astrocytes harboring different *APOE* genotypes. Given differences in lipid metabolism between *APOE* mouse and human models^37^, we chose to investigate this question in human iPSC-derived astrocytes. We applied sucrose gradient ultracentrifugation to isolate lipid droplets^3^ from iPSC-derived astrocytes treated with oleic acid and subjected these lipid droplets to mass-spectrometry based proteomic and lipidomic analyses. These analyses identified proteins that associate with oleic acid-induced lipid droplets in human astrocytes. While many of these proteins had been found to associate with lipid droplets in other cell types, some were unique to astrocytes and suggested astrocyte-specific lipid droplet functions. Moreover, we found that the *APOE* genotype influenced both the lipid and protein composition of lipid droplets, indicating that AD-associated genetic variation can reshape astrocytic cell biology via remodeling of the lipid droplet landscape. Specifically, we identified that lipid droplets in *APOE2* and *APOE4* astrocytes, which both accumulate to high levels, have different propensities for lipophagy, which impact their dynamics. Together, we present a resource for the lipid droplet and neurodegenerative disease communities, offering new insight into how the most impactful genetic modifier of AD risk, *APOE*, can alter the composition and dynamics of an organelle increasingly implicated in brain health and disease.

## Results

### Sucrose gradient ultracentrifugation isolates lipid droplets from human iPSC-derived astrocytes

**Fig. 1:**
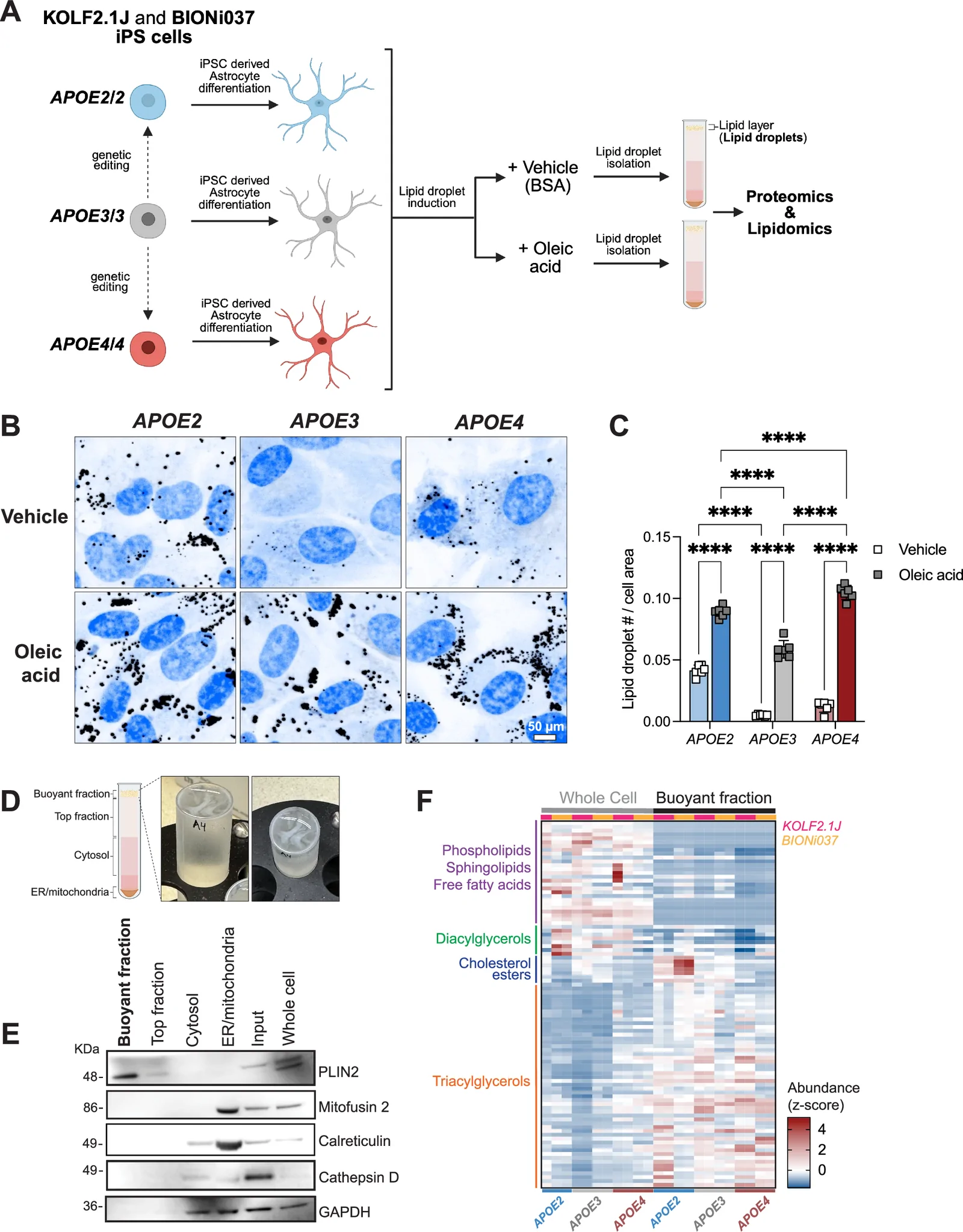
Lipid droplets can be isolated from human iPSC-derived astrocytes. **A** Schematic of lipid droplet isolation and analysis workflow using two isogenic iPSC-derived astrocyte sets homozygous for *APOE2*, *APOE3*, or *APOE4*, generated from KOLF2.1 J and BIONi037A parental lines. Created in BioRender. Narayan, P. (2026) https://BioRender.com/ym8bxun**B** Neutral lipid immunofluorescence in KOLF2.1 J iPSC-derived astrocytes homozygous for the three common *APOE* genotypes and treated with vehicle (BSA) or 80 μM oleic acid. LipidSpot is shown in black, and Hoechst 33258-stained nuclei in blue. Scale bar, 50 μm. **C** Quantification of lipid droplets per cell area across *APOE* genotypes and treatments in KOLF2.1 J astrocytes. Each point represents *n* = 6 independent treatments; data are mean ± SD. ****p ≤ 10⁻¹¹ by two-way ANOVA with post-hoc Šídák test. **D** Images of the buoyant fraction after sucrose gradient ultracentrifugation. Created in BioRender. Narayan, P. (2026) https://BioRender.com/iad9z5u. **E** Western blot of the buoyant fraction for organelle and compartment markers: PLIN2 for lipid droplets, Mitofusin 2 for mitochondria, Calreticulin for ER, Cathepsin D for lysosomes, and GAPDH for cytosol. **F** Heatmap comparing mass spectrometry lipidomics measurements of unfractionated whole-cell and buoyant-fraction samples after fractionation. Values represent z-scored abundance normalized to total lipid content per sample. *N* = 11–12 independent iPSC-derived astrocyte samples and fractionations are shown, with multiple lipid classes represented.

Astrocytes produce the majority of APOE in the brain and accumulate different numbers of lipid droplets depending on their *APOE* genotype^14,24^. To ensure rigor and reproducibility, we chose to isolate lipid droplets from human iPSC-derived astrocytes derived from two iPSC sets (KOLF2.1 J and BIONi037)^38,39^(Fig. 1A). Each cell set contains three cell lines that are isogenic at all loci except for *APOE*, where they are homozygous for each of the common variants: *APOE2, APOE3*, or *APOE4*. We differentiated these cells into iPSC-derived astrocytes using established protocols^30,40^. These cells express canonical astrocytic markers (Supplementary Fig. S1A, B) and accumulate lipid droplets at different levels. We have previously reported findings of increased lipid droplets in *APOE4* astrocytes compared to *APOE3* astrocytes^24^. We recapitulate this finding and observe that *APOE4, APOE2*, and *APOE-KO* iPSC-derived astrocytes also accumulate lipid droplets relative to *APOE3* (Supplementary Fig. S1C, D). Given the recent research showing that lipid droplets influence disease-associated processes in AD^10–14,17,22,24^, we hypothesized that the molecular composition of the lipid droplets in *APOE2* astrocytes and *APOE4* astrocytes was different and could relate to the alleles’ divergent effects on AD risk.

To maximize the number of lipid droplets in our iPSC-derived astrocytes, we stimulated lipid droplet biogenesis by treatment with oleic acid and a vehicle control (bovine serum albumin, BSA). Under vehicle-treated conditions, lipid droplet accumulation varied across the two isogenic iPSC-derived astrocyte sets, with genotype-dependent differences more apparent in the KOLF2.1J-derived astrocytes (Fig. 1B, C) than in the BIONi037-derived astrocytes (Supplementary Fig. S1E, F). These differences may reflect parental genetic background, differentiation method, or culture conditions. Oleic acid treatment robustly increased lipid droplet accumulation across all *APOE* genotypes and both iPSC-derived astrocyte sets (Fig. 1B, C and Supplementary Fig. S1E–G). We used oleic acid treatment as a controlled lipid-loading paradigm to increase lipid droplet biogenesis and obtain sufficient lipid droplet material for organelle-level molecular analyses. Although this approach does not model amyloid exposure or Alzheimer’s disease directly, it represents a lipid-enriched cellular state that facilitates detailed analysis of lipid droplet composition.

To fully characterize our model system, we measured the intracellular and extracellular levels of APOE protein in the presence and absence of oleic acid treatment. We observed that oleic acid treatment decreases both intracellular (Supplementary Fig. S2A, B) and secreted APOE levels (Supplementary Fig. S2D–G) compared to vehicle control treatment. Moreover, we detected APOE protein on buoyant fractions after oleic acid treatment, and the levels were similar across genotypes (Supplementary Fig. S2C). We also imaged the subcellular localization of APOE with and without oleic acid and observed that across all genotypes, APOE does not localize primarily with lipid droplets but rather exists throughout the secretory pathway (Supplementary Fig. S2H).

We then applied sucrose gradient density ultracentrifugation protocols to isolate lipid-rich “buoyant” fractions from iPSC-derived astrocytes treated with either oleic acid or vehicle (Fig. 1D). This buoyant fraction was lipid droplet-rich and showed no detectable contamination from other organelles, including lysosomes, mitochondria, and ER (Fig. 1E). When compared to the whole cell by lipidomics, the buoyant fraction was enriched for lipids canonically present in lipid droplets like triacylglycerols and cholesterol esters (Fig. 1F and Supplementary Data 15–20). We next looked at the distribution of oleic acid-containing lipid classes and observed that oleic acid supplementation primarily impacts the lipid composition of the buoyant fraction compared to the whole cell (Supplementary Fig. S3A and Supplementary Data 21). The strongest differences were on lipid droplet storage lipids, including triacylglycerols and cholesterol esters, as well as their precursors—diacylglycerols and free oleic acid (Supplementary Fig. S3A). Far more subtle differences were detected in phospholipid classes (Supplementary Fig. S3A). It is important to note that the cellular lipid composition of iPSC-derived astrocytes is impacted by culturing conditions, as our whole cell lipidomics findings differed from a previous study^30^ (Supplementary Fig. S3B). A side-by-side comparison between our protocol (48 h treatment with 0.7% BSA in FBS-free media) and that used in Feringa et al. (FBS-free media for 24 h) revealed changes in the abundance of lipid classes, particularly neutral lipids, in an *APOE* genotype-specific manner (Supplementary Fig. S3C and Supplementary Data 22).

Together, these findings confirmed that our buoyant fractions were enriched in lipid droplets and could be further analyzed to reveal molecular features of these understudied organelles in iPSC-derived astrocytes. We applied mass spectrometry-based proteomics to multiple cellular fractions (buoyant and cytosolic) from each astrocyte line. When comparing proteins in the oleic acid- and vehicle-treated samples, we did not find proteins that were differentially present in the buoyant fraction (Supplementary Fig. S4A and Supplementary Data 2). This finding gave us confidence that oleic acid treatment enables us to isolate more lipid droplets from the same amount of cell material, without substantially altering the proteomic composition of the lipid droplets. Given that the amount and identity of buoyant fraction proteins detected was largely similar between oleic acid- and vehicle-treated conditions, we performed all subsequent analyses on oleic-acid-treated fractions to maximize coverage of lipid droplet-associated proteins.

### The human iPSC-derived astrocyte lipid droplet-associated proteome contains known and new proteins

Our first goal after successfully isolating lipid droplet-enriched fractions was to establish a cell type-specific lipid droplet-associated proteome for human iPSC-derived astrocytes. Most past studies of lipid droplet proteomes were performed in non-human organisms or cancer cell lines^3,6,41^. Given the emerging role of lipid droplets in brain diseases and the central role of astrocytes in brain lipid homeostasis^12,14^, we aimed to define a human iPSC-derived astrocyte lipid droplet-associated proteome.

**Fig. 2:**
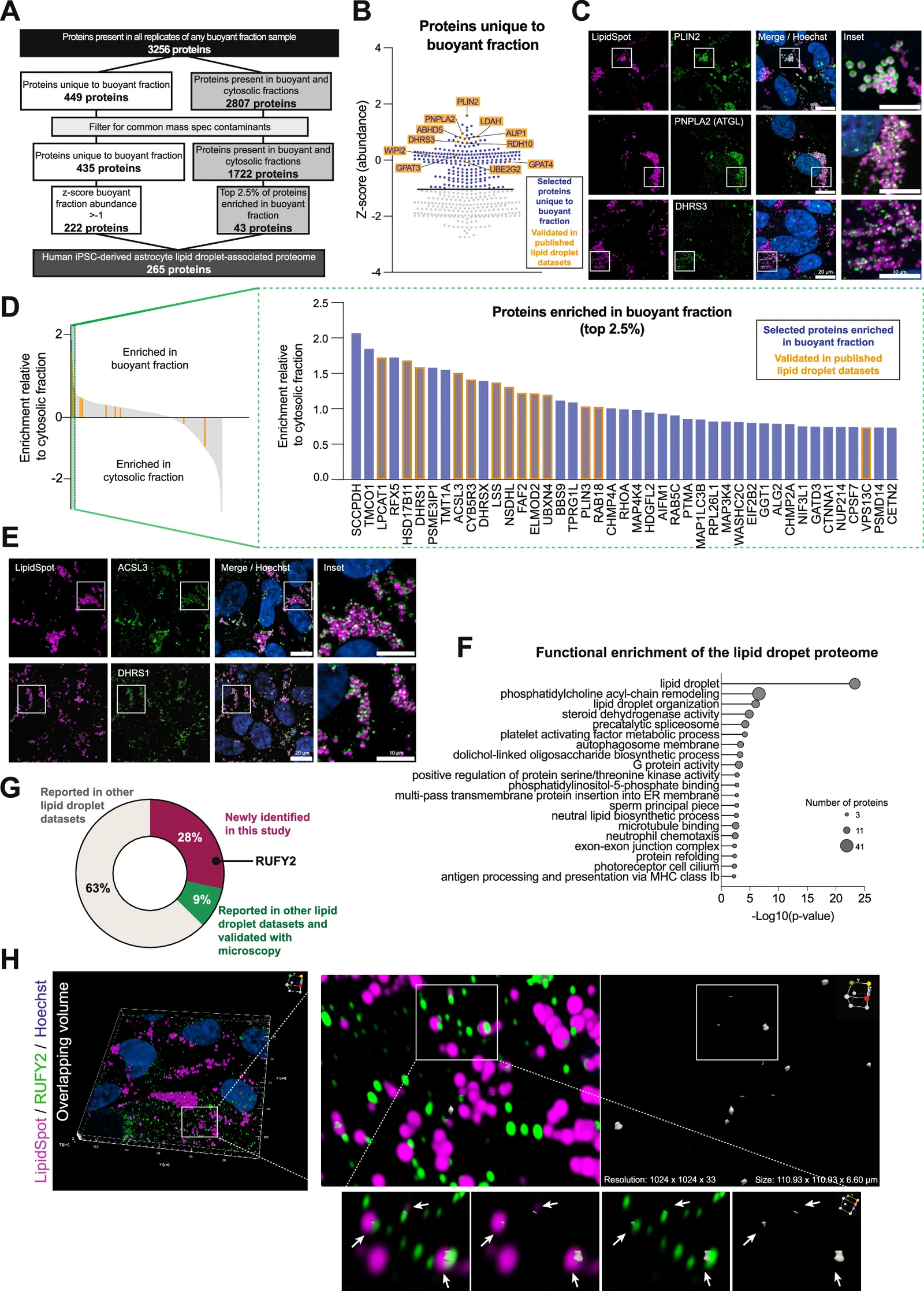
Defining the human astrocytic lipid droplet-associated proteome. **A** Flowchart of proteomic analysis identifying the human iPSC-derived astrocyte lipid droplet-associated proteome across *APOE2*, *APOE3*, and *APOE4* astrocytes from two isogenic iPSC lines, 3256 proteins were detected in buoyant fractions. Compared with the cytosol, 499 proteins were unique, and 2807 were enriched. After removing common mass spectrometry contaminants and applying stringent cutoffs, 222 unique and 43 enriched proteins remained, defining a 265-protein astrocytic lipid droplet-associated proteome. **B** Mean z-score across oleic acid-treated samples for proteins detected in both technical replicates of at least one buoyant fraction sample and absent from the cytosol. Blue dots indicate proteins included in the proteome (mean z-score > − 1); orange outlines indicate canonical lipid droplet proteins validated by immunofluorescence here or elsewhere. **C** Immunofluorescence of canonical lipid droplet proteins PLIN2, PNPLA2/ATGL, and DHRS3 in green localized to LipidSpot-positive lipid droplets in magenta in KOLF2.1 J astrocytes. Scale bars: overview, 20 μm; inset, 10 μm. Representative images from 3-4 experiments. **D** Waterfall plot showing enrichment scores relative to cytosol for proteins present in both buoyant and cytosolic fractions. The inset shows the top 2.5% of enriched proteins included in the proteome (blue bars); excluded contaminants are gray. Orange outlines indicate selected canonical lipid droplet proteins. **E** Immunofluorescence of ACSL3 and DHRS1 in green localized to LipidSpot-positive lipid droplets in magenta in KOLF2.1 J astrocytes. Scale bars: overview, 20 μm; inset, 10 μm. Representative images from 3 experiments. **F** Overrepresentation analysis of the 265-protein proteome using a standard accumulative hypergeometric test without multiple-comparison adjustment. Benjamini–Hochberg-adjusted p-values are in supplementary data files. **G** Pie chart showing the percentage of lipid droplet-associated proteins uniquely identified in this study. **H** Immunofluorescence of RUFY2 in green, contacting LipidSpot-positive lipid droplets in magenta in BIONi037A astrocytes. Arrows indicate lipid droplets interacting with RUFY2; overlap volume is white. Scale: 111 × 111 × 6.60 μm³. Representative images from 5 experiments.

We started with the 3256 proteins detected in all technical replicates of any of our oleic acid-treated buoyant fraction samples (Supplementary Data 3). Of these 3256 proteins detected, 449 were unique to the buoyant fraction and absent in the cytosolic fraction, and 2807 proteins were present in both fractions (Fig. 2A and Supplementary Data 4, 5). To determine the iPSC-derived astrocyte lipid droplet-associated proteome, we considered these two sets (unique to buoyant fraction and shared between buoyant and cytosolic fraction) separately. To account for mass spectrometry contaminants commonly detected in subcellular fractionation experiments^42^, we first removed them from our initial protein lists, resulting in 435 proteins unique to the buoyant fraction and 1722 proteins common to both buoyant and cytosolic fractions (Supplementary Data 1). We first used a mean z-score > −1 filter on the 435 proteins unique to the buoyant fraction samples to select the most abundant 222 proteins (Fig. 2A, B and Supplementary Data 6). These selected proteins included many canonical and well-established lipid droplet proteins, such as PLIN2, PNPLA2 (ATGL), and DHRS3, which we were able to confirm localized to lipid droplets using immunofluorescence (Fig. 2C). For the 1722 proteins in the buoyant fraction that were also detected in cytosolic fractions, we calculated the relative enrichment of these proteins between the buoyant and cytosolic fractions and included only 43 proteins (those with the top 2.5% of enrichment scores, approximately two standard deviations from the mean) in our iPSC-derived astrocyte lipid droplet-associated proteome (Fig. 2A, D and Supplementary Data 5). We chose a stringent threshold to avoid false positives. Among these 43 proteins were multiple known lipid droplet proteins that had been previously identified and validated in other studies, like ACSL3 and DHRS1^43,44^. These proteins are known to traffic to the lipid droplet from other cellular compartments. We confirmed the lipid droplet localization of ACSL3 and DHRS1 in iPSC-derived astrocytes by immunofluorescence (Fig. 2E).

We combined these two categories of proteins, those unique to the buoyant fraction (222) and those strongly enriched in the buoyant over the cytosolic fraction (43), to compile an initial human iPSC-derived astrocyte lipid droplet-associated proteome consisting of 265 proteins (Supplementary Fig. S4B and Supplementary Data 7). We used overrepresentation analysis^45^ to determine the functional enrichment of these proteins and reassuringly found that they primarily were associated with lipid droplets and lipid metabolism (Fig. 2F and Supplementary Data 8). In addition to the canonical functions in lipid storage, the iPSC-derived astrocyte lipid droplet-associated proteome was enriched in non-canonical functions. These include functions related to the spliceosome, which has been previously linked to lipid droplet biology^46^, as well as antigen presentation. Interestingly, antigen presentation has recently been linked to astrocyte lipid state, but not directly through the astrocytic lipid droplet proteome^30^ and major histocompatibility complex II (MHCII) molecules have been found to co-localize to lipid droplets in iPSC-derived astrocytes^47^.

Of our iPSC-derived astrocyte lipid droplet-associated proteome (265 proteins, Supplementary Data 7), 72% of proteins had been previously identified in other datasets. The remaining 28% of proteins were newly identified in this study (Fig. 2G). One such protein is RUFY2, which we confirmed co-localized with lipid droplets in iPSC-derived astrocytes (Fig. 2H), but also associates with other organelles, including early endosomes and the Golgi (Supplementary Fig. S4C). Together, our proteomics analysis revealed a high-confidence lipid droplet-associated proteome in  human iPSC-derived astrocytes and identified proteins that distinguish glial cell lipid droplets from other cell types.

### *APOE* genotype shapes the lipid droplet-associated proteome

**Fig. 3:**
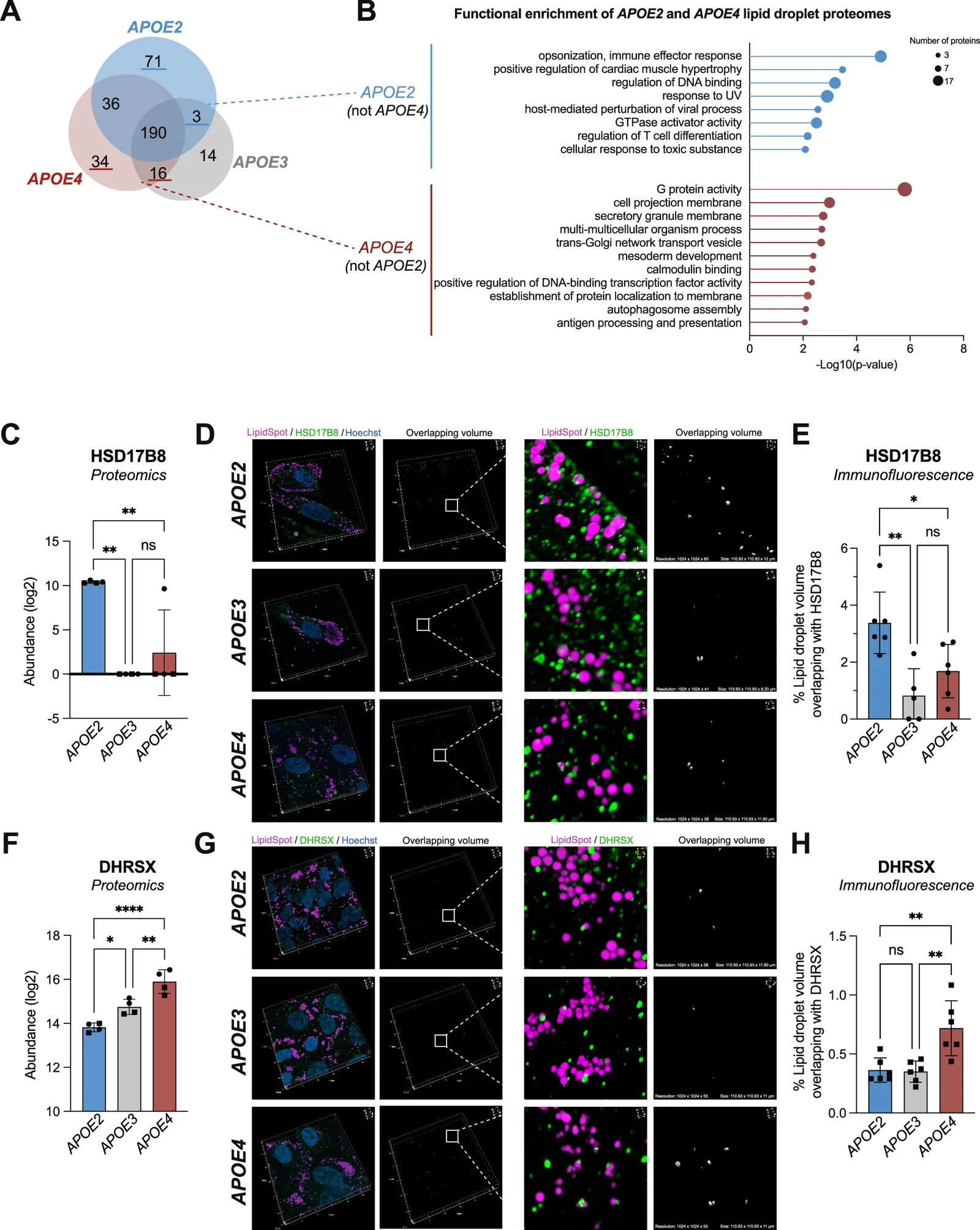
*APOE* genotype changes the lipid droplet-associated proteome. **A** Venn diagram showing buoyant-fraction proteins shared across or specific to iPSC-derived astrocytes homozygous for *APOE2*, *APOE3*, or *APOE4*. Underlined protein groups were used for overrepresentation analysis in (**B**). **B** Overrepresentation analysis of proteins present in *APOE2* lipid droplet-associated proteomes, including 71 *APOE2*-exclusive and 3 *APOE2*/*APOE3*-shared proteins, or *APOE4* proteomes, including 34 *APOE4*-exclusive and 16 *APOE4*/*APOE3*-shared proteins. A standard accumulative hypergeometric test was used without multiple-comparison adjustment; Benjamini–Hochberg-adjusted p-values are in supplementary data. **C** HSD17B8 abundance in buoyant fractions across *APOE* genotypes by mass spectrometry. Data are *n* = 4 independent replicates across two isogenic human iPSC-derived astrocyte sets; mean ± SD. **p ≤ 0.01 by one-way ANOVA with Tukey’s test; *APOE2*–*APOE4*, *p* = 0.0073; *APOE2*–*APOE3*, *p* = 0.0013. **D** Immunofluorescence of HSD17B8 in green and LipidSpot-positive lipid droplets in magenta in BIONi037A astrocytes. Overlap volume is white. Representative high-magnification fields; 3D projection: 111 × 111 × 8.20–12 μm³. Representative images from 6 experiments. **E** Percent lipid droplet volume overlapping HSD17B8 signal by 3D confocal imaging. Data represent 400–700 lipid droplets per frame and *n* = 5–6 frames per *APOE* genotype; mean ± SD. *p = 0.0267, **p = 0.0022 by one-way ANOVA with Tukey’s test. **F** DHRSX abundance in buoyant fractions across *APOE* genotypes by mass spectrometry. Data are *n* = 4 independent replicates across two isogenic astrocyte sets; mean ± SD. *p = 0.0187, **p = 0.0057, ****p = 0.000085. **G** Immunofluorescence of DHRSX in green, contacting LipidSpot-positive lipid droplets in magenta in KOLF2.1 J astrocytes. Overlap volume is white. Representative high-magnification fields; 3D projection: 111 × 111 × 11–11.6 μm³. Representative images from 6 experiments. **H**. Percent lipid droplet volume overlapping DHRSX signal by 3D confocal imaging. Data represent 700–1000 lipid droplets per frame and *n *= 6 frames per *APOE* genotype; mean ± SD. **p ≤ 0.01 by one-way ANOVA with Tukey’s test; *APOE2*–*APOE4*, *p* = 0.0035; *APOE3*–*APOE4*,* p* = 0.0026.

Given that the *APOE* genotype influences lipid droplet abundance in astrocytes, we sought to determine how it alters the lipid droplet-associated proteome. We observed that the genetic background of the parental iPSCs lines partially drove the variability observed across *APOE*-specific lipid droplet proteomes (Supplementary Fig. S5A). In order to ensure that our findings were independent of genetic background (and sex) differences, we focused exclusively on proteins and abundance patterns consistently observed across both iPSC lines sharing the same *APOE* genotype from two different isogenic sets. We applied the same approach as previously described to the proteins found in the buoyant fraction of each genotype to define *APOE* genotype-specific astrocytic lipid droplet proteomes (Supplementary Data 9,10). When comparing proteins within the lipid droplet buoyant fractions across genotypes, we found that although the majority of proteins were shared, each genotype exhibited a distinct subset of proteins, with *APOE2* containing the highest number of genotype-specific proteins, followed by *APOE4* and *APOE3* (Fig. 3A and Supplementary Data 11). As expected, functional enrichment analysis of the shared proteome reflected the pathways present in the iPSC-derived astrocyte lipid droplet-associated proteome (Supplementary Fig. S5B). We then focused on comparing the genotype-specific proteomic signatures of *APOE2* and *APOE4* buoyant fractions. Proteins detected on *APOE2* lipid droplets, and not found in *APOE4*, were enriched in immune and cellular response pathways. In contrast, proteins present on *APOE4* lipid droplets but not found in *APOE2* showed functional enrichment in protein secretion and autophagosome assembly pathways (Fig. 3B and Supplementary Data 12, 13). These findings suggest *APOE* genotype-specific roles for lipid droplets beyond that of lipid storage. Studies in other models have identified lipid droplets as immune hubs that dock immune proteins^4^. Lipid droplets have also been observed to participate in cellular trafficking and ER biology^48^.

We also identified proteins that were associated with lipid droplets in all three *APOE* genotypes, but to different extents across the genotypes (Supplementary Fig. S5C and Supplementary Data 9). For example, we observed that HSD17B8, a protein involved in steroid and fatty acid metabolism^49^, was mainly detected in our *APOE2* astrocytes (Fig. 3C). Using immunofluorescence, we observed that HSD17B8 showed greater co-localization with lipid droplets in *APOE2* astrocytes than in *APOE3* or *APOE4* astrocytes (Fig. 3D, E). DHRSX, a protein involved in multiple steps of dolichol biosynthesis^50^, was more abundantly associated with *APOE4* lipid droplets than in the other two *APOE* genotypes (Fig. 3F), and we were able to confirm this with immunofluorescence (Fig. 3G, H).

We analyzed human astrocyte transcriptomes taken from four published single-nuclei transcriptomic datasets to determine whether genotype-specific lipid droplet proteome changes are reflected in post-mortem brain samples^51–54^. Many lipid droplet protein transcripts were reliably detected in astrocytes. Stratifying samples by *APOE* genotype (*APOE2*-carriers *n* = 39*, APOE3*-homozygous *n* = 172*, APOE4*-carriers *n* = 87) revealed differential expression patterns consistent with our proteomics data (Supplementary Fig. S5D and Supplementary Data 14). For instance, the *APOE2* lipid droplet-associated protein ADK (linked to toxin response) was elevated in *APOE2*-carriers, while the *APOE4* lipid droplet-associated proteins RAB8A and MYO1E (involved in intracellular trafficking) were enriched in *APOE4*-carriers (Supplementary Data 14). In addition, transcripts for the lipid droplet proteins HSD17B8 and DHRSX varied by genotype, mirroring our proteomics results (Supplementary Fig. S5E). The autophagosome marker *MAP1LC3B* (LC3B) was also more abundant in *APOE4* compared to *APOE2* carriers (Supplementary Fig. S5E).

Together, these findings cement that iPSC-derived astrocytes harboring different *APOE* genotypes have distinct lipid droplet-associated proteomes that align with transcriptomic patterns in human post-mortem brains. These genotype-specific proteins may underlie functional differences in lipid droplet biology between astrocytes.

### *APOE* genotype shapes the lipid droplet lipidome

**Fig. 4:**
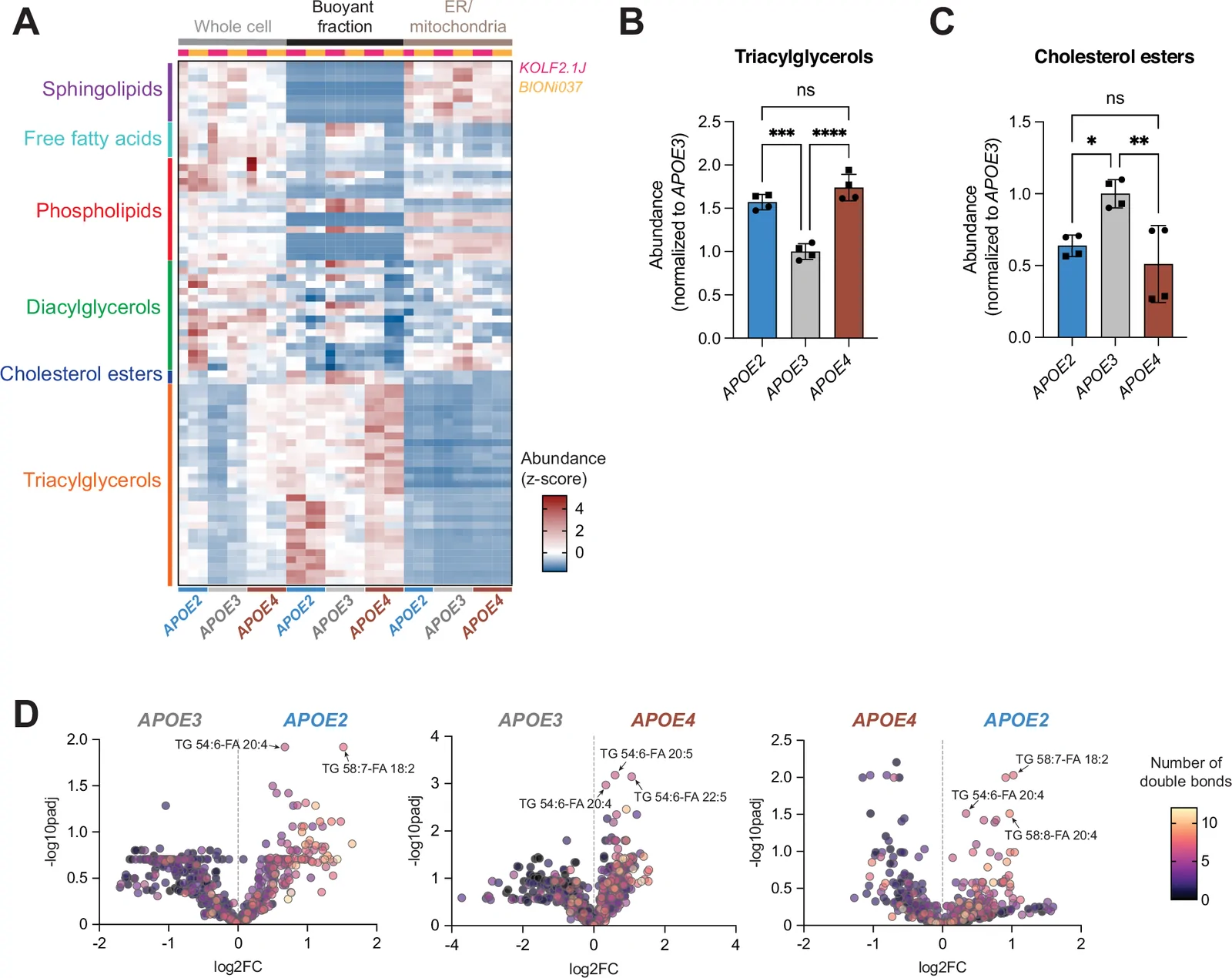
*APOE* genotype changes the lipid droplet lipidome. **A** Heatmap of mass spectrometry lipidomics measurements from unfractionated whole cells, buoyant lipid droplet fractions, and ER/mitochondria fractions from oleic acid-treated iPSC-derived astrocytes across two isogenic sets. Values represent z-scored abundance normalized to total lipid content. *N* = 11–12 independent samples and fractionations are shown, with multiple lipid classes represented. **B** Triacylglycerol abundance in buoyant fractions across *APOE* genotypes from oleic acid-treated astrocytes. Data represent mean ± SD of *n* = 4 independent replicates across two isogenic human iPSC-derived astrocyte sets. ***p ≤ 0.001, ****p ≤ 0.0001 by one-way ANOVA with Tukey’s test; *APOE2*–*APOE3*, *p* = 0.00016; *APOE3*–*APOE4*, *p* = 0.00002. **C** Cholesterol ester abundance in buoyant fractions across *APOE* genotypes from oleic acid-treated astrocytes. Data represent mean ± SD of *n* = 4 independent replicates across two isogenic human iPSC-derived astrocyte sets. *p ≤ 0.05, **p ≤ 0.01 by one-way ANOVA with Tukey’s test; *APOE2*–*APOE3*, *p* = 0.036; *APOE3*–*APOE4*, *p* = 0.0073. **D** Volcano plots comparing buoyant-fraction lipids across *APOE* genotypes in oleic acid-treated astrocytes. Color indicates fatty acid saturation, shown as the number of double bonds. *P*-values were determined by *t* test and adjusted using the Benjamini–Hochberg correction.

We hypothesized that the effect of *APOE* genotype on the astrocytic lipid droplet-associated proteome may arise from changes to lipid droplet lipid composition. To investigate this, we analyzed the lipid composition of lipid droplets from oleic acid- and vehicle-treated iPSC-derived astrocytes of various *APOE* genotypes using LC-MS-based lipidomics. When we compared the overall lipid profile of the buoyant fraction with that of the whole cell and the ER/mitochondria fraction, we observed that each cellular compartment had a unique lipid profile (Fig. 4A). In addition, we noticed that some *APOE* genotype-dependent lipid changes at the organellar level were not captured in the whole cell data (Supplementary Data 15–20, 23, 24). This finding reveals that *APOE* genotype modulates lipid composition at the subcellular level and highlights the importance of studying lipid biology from an organellar perspective.

When comparing the levels of triacylglycerols, the main lipid components of lipid droplets after oleic acid treatment in the buoyant fraction, we observed that these correlated well to the quantity of lipid droplets in the iPSC-derived astrocytes of various *APOE* genotypes (Fig. 4B). Lipid droplets from *APOE3* iPSC-derived astrocytes showed high cholesterol esters, diacylglycerols and fatty acids but reduced triacylglycerols, suggesting a lower capacity to efficiently store fatty acids as triacylglycerols when stimulated with oleic acid (Fig. 4B, C and Supplementary Fig. S6A, B). On the other hand, lipid droplets from *APOE2* and *APOE4* iPSC-derived astrocytes had similar quantities of cholesterol esters and triacylglycerols (Fig. 4B, C). Because the lipid droplet-associated proteomes of these genotypes were distinct, we reasoned that changes in relative lipid class were not the driver of lipid droplet-associated proteome changes.

Previous work has identified that the *APOE* genotype correlates with changes in fatty acid saturation in whole cell lipidomics measurements^16,24,30^. When examining the saturation of fatty acids within lipid droplet lipids, we observed that *APOE2* contains far more unsaturated lipids than *APOE3* and *APOE4* (Fig. 4D and Supplementary Data 25). This change was not reflected in our whole cell lipidomics data (Supplementary Fig. S6C and Supplementary Data 26), reinforcing the importance of cellular compartment-specific analyses. Given that our experimental paradigm involved treating our iPSC-derived astrocytes with the monounsaturated fatty acid oleic acid to increase lipid droplet content, we confirmed that the trend we observed of increased unsaturation in *APOE2* lipid droplets held true under basal conditions (treated with BSA vehicle) (Supplementary Fig. S6D and Supplementary Data 25). Differences in saturation between genotypes were mainly driven by triacylglycerol containing polyunsaturated fatty acid (PUFA) species, such as TG 54:6-FA 20:4 and TG 58:7-FA 18:2 (Fig. 4D, Supplementary Fig. S6D and Supplementary Data 25). Several biophysical studies have reported that lipid droplets with higher levels of unsaturation exhibit packing defects and increased protein binding^55,56^. Moreover, the sequestration of membrane PUFA lipids into lipid droplets could be protective, as it prevents peroxidation and subsequent lipotoxicity^57^. Given that lipid droplets from *APOE2* astrocytes had both the highest unsaturation of lipids and the greatest number of proteins bound, we concluded that the *APOE* genotype could be driving changes in lipid droplet-associated proteomes by modulating lipid saturation.

### Lipid droplet-associated proteomes modulate lipid droplet dynamics

**Fig. 5:**
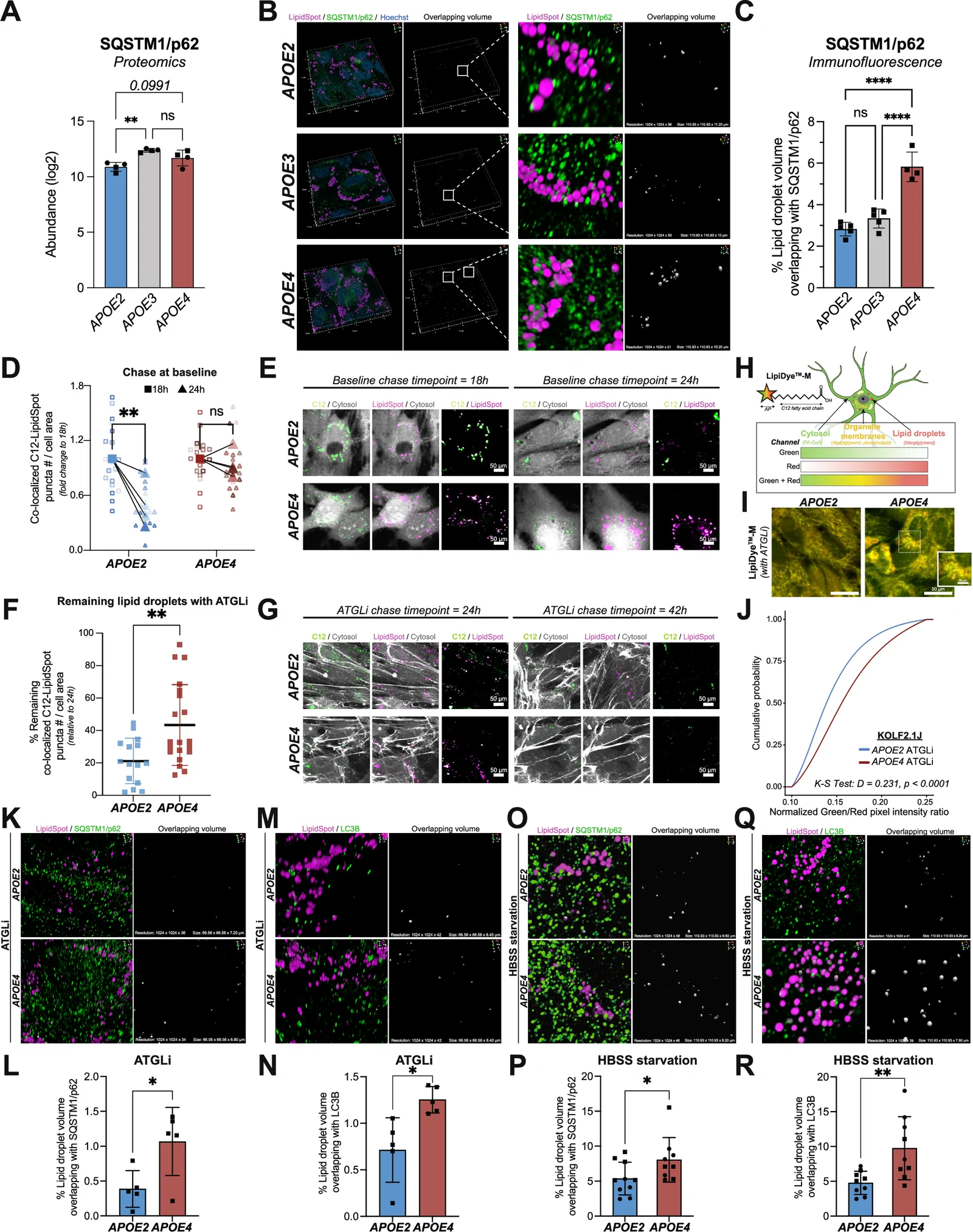
*APOE* genotype-specific lipid droplet-associated proteins change lipophagy. **A** SQSTM1/p62 abundance in buoyant fractions by MS. *N* = 4 independent replicates across two isogenic iPSC-derived astrocyte sets; mean ± SD. **p = 0.0046 by one-way ANOVA with Tukey’s test. **B** SQSTM1/p62 in green contacting LipidSpot-positive lipid droplets in magenta in KOLF2.1 J astrocytes; overlap volume is white. 3D projection: 110.93 × 110.93 × 10–11.20 μm³. **C** Percent lipid droplet volume overlapping SQSTM1/p62. Approximately 700 lipid droplets across 10–20 cells per replicate; *n* = 4–5 frames per *APOE* genotype; mean ± SD. ****p ≤ 0.0001 by one-way ANOVA with Tukey’s test; *APOE2*–*APOE4*, *p* = 0.000006; *APOE3*–*APOE4*, *p* = 0.000038. **D** Number of BODIPY-C12/LipidSpot-positive lipid droplets during an 18–24 h chase in KOLF2.1J astrocytes. Large symbols, *n* = 4 independent experiments; small symbols, technical replicates. **p = 0.0059 by repeated-measures two-way ANOVA with Šidák’s test. **E** Representative BODIPY-C12/LipidSpot-positive lipid droplets during chase. Scale bars, 50 μm. **F** Remaining BODIPY-C12/LipidSpot-positive lipid droplets after 42 h chase with 10 μM ATGLi in KOLF2.1J *APOE2* and *APOE4* astrocytes, normalized to 24 h. *N* = 15–20 replicates; mean ± SD. **p = 0.004 by two-sided unpaired *t* test. **G** Representative 24 h and 42 h ATGLi pulse-chase images. Scale bars, 50 μm. **H**,** I** LipiDye™-M schematic and representative images of KOLF2.1J *APOE2* and *APOE4* astrocytes treated with ATGLi for 96 h. Scale bars, 50 μm. Created in BioRender. Cuni-Lopez, C. (2026) https://BioRender.com/zlt6nvf**J** Cumulative distribution of LipiDye™-M green/red pixel intensity ratio after ATGLi. At least 180 cells per condition; *p* < 0.0001 by two-sided Kolmogorov–Smirnov test. **K**–**N** SQSTM1/p62 or LC3B overlap with lipid droplets after 96 h ATGLi treatment in KOLF2.1J astrocytes. Representative 3D projections and quantification show percent lipid droplet volume colocalizing with each marker; n = 5 frames per genotype; mean ± SD. SQSTM1/p62, *p = 0.025; LC3B, *p = 0.012 by two-sided unpaired *t* test. **O**–**R** SQSTM1/p62 or LC3B overlap with lipid droplets after 1 h HBSS starvation across two isogenic astrocyte sets. Representative 3D projections and quantification show percent lipid droplet volume colocalizing with each marker; *n* = 9–10 frames per genotype; mean ± SD. SQSTM1/p62, *p = 0.050; LC3B, **p = 0.0069 by two-sided unpaired *t* test.

Since *APOE* genotype modulates both the lipid droplet-associated proteome and lipidome in iPSC-derived astrocytes, we asked whether these changes have functional consequences. One of the enriched functional categories in the *APOE4* lipid droplet-associated proteome was autophagosome assembly (Fig. 3B and Supplementary Data 13). Our dataset revealed that in *APOE4* astrocytes, lipid droplets contained higher levels of autophagosome assembly proteins than *APOE2* astrocytes, even though both *APOE2* and *APOE4* astrocytes exhibit higher numbers of lipid droplets than *APOE3* astrocytes. We focused on SQSTM1 (p62), a selective autophagy receptor protein that has been shown to encapsulate lipid droplets during autophagy^58^. SQSTM1/p62 binds ubiquitinated proteins and organelles and recruits them to autophagosomes for degradation^59^. Our proteomics data showed a trend that SQSTM1/p62 was more abundant on *APOE4* lipid droplets than *APOE2* lipid droplets (Fig. 5A). We observed by immunofluorescence that the levels of co-localized SQSTM1/p62 with lipid droplets were indeed higher in *APOE4* than *APOE2* astrocytes (Fig. 5B, C and Supplementary Fig. S7A, B).

One route for lipid droplet turnover is via lipophagy–the selective autophagy of lipid droplets–which involves ubiquitination and binding to autophagy adapter proteins like SQSTM1/p62^1^. Given the differences in lipid droplet-associated proteome composition, we hypothesized that *APOE4* astrocytes may display an impairment in lipophagy, leading to an accumulation of lipid droplets bound to SQSTM1/p62^60^. To test this hypothesis, we first measured the overall turnover rate of fatty acids within lipid droplets in *APOE2* and *APOE4* astrocytes by using an established pulse-chase experimental paradigm with fluorescent BODIPY-C_12_ fatty acids^61,62^. C_12_ fatty acids within lipid droplets persisted longer in *APOE4* than in *APOE2* astrocytes following a 24 h chase period under baseline conditions (Fig. 5D, E and Supplementary Fig. S7C), suggesting that lipid droplet turnover is slower in *APOE4*.

In hepatocytes, lipid droplet catabolism has been reported to occur as a sequential, two-step process^63^. First, triglyceride-rich lipid droplets are degraded via lipolysis mediated by the rate-limiting lipase adipose triglyceride lipase (ATGL). Then the remaining lipid droplets are tagged with autophagic adapters, including SQSTM1/p62 and LC3B, and undergo lipophagic degradation in acidic vesicles. To isolate lipophagy-mediated turnover in *APOE2* and *APOE4* astrocytes, we first inhibited lipolysis using the chemical inhibitor of ATGL, atglistatin (ATGLi). We observed that ATGLi showed a measurable and significant change in lipid droplet degradation in astrocytes after a 72 h treatment, that was maintained up to 96 h without re-adding the drug (Supplementary Fig. S7D, E). Next, we monitored the turnover of C_12_-positive lipid droplets by repeating the pulse-chase assay under lipolysis inhibition (with ATGLi) conditions. We observed a lack of lipid droplet turnover within the initial 24 h chase period compared to vehicle (DMSO), implying that breakdown is initially mediated by lipolysis (Supplementary Figure S7F, G). At the 42 h chase period, when lipolysis is still inhibited (equivalent to ATGLi treatment for 96 h), a remaining ~ 20% of lipid droplets from the initial pool persisted in *APOE2* compared to ~ 40% in *APOE4* (Fig. 5F, G and Supplementary Fig. S7H), suggesting a faster lipophagy rate in *APOE2* astrocytes.

To measure lipophagy directly, we used LipiDye^TM^-M^64^, a fluorescent C_12_ fatty acid probe appended to an azapyrene (AP) dye that enables the visualization of the intracellular distribution of fatty acid-derived metabolites and has been used to quantify lipophagy^65^. LipiDye^TM^-M emits multicolor fluorescence depending on its surrounding environment: it is green in aqueous cell compartments like the cytosol, yellow when it is incorporated into medium-polarity organellar membranes, and red when is taken up by non-polar environments like lipid droplets (Fig. 5H). In order to confirm that LipidDye^TM^-M could faithfully show stalled lipophagy in human iPSC-derived astrocytes, we incubated LipiDye^TM^-M in the presence of the autophagy inhibitor bafilomycin A1. We observed that bafilomycin A1 treatment in astrocytes induced the accumulation of large vesicles with strong red and green co-localized fluorescence signals (yellow), reminiscent of autophagosomes (Supplementary Fig. S8A–D). The green-to-red pixel intensity ratio was higher (right shift) under impaired autophagy conditions compared to vehicle across all *APOE* genotypes (Supplementary Fig. S8B, D). This led us to quantify the LipiDye^TM^-M signal in *APOE2* and *APOE4* iPSC-derived astrocytes under lipolysis inhibition with ATGLi after 96 h of treatment, a timepoint where we showed by pulse-chase assay that lipid degradation occurs via lipophagy. The LipiDye^TM^-M ratiometric analysis revealed a higher green-to-red pixel intensity ratio in *APOE4* than *APOE2* (Fig. 5I, J and Supplementary Fig. S8E, F), similar to the positive bafilomycin A1-treated control, suggesting impaired lipophagy in *APOE4* astrocytes.

The incubation with the LipiDye^TM^-M probe under ATGLi conditions revealed autophagosome-like structures that were more abundant in *APOE4* than *APOE2* astrocytes (Fig. 5I and Supplementary Fig. S8E). Therefore, we next sought to confirm the identity of these vesicles by immunofluorescence for autophagy markers. Using the autophagy inhibitors bafilomycin A1 and chloroquine as positive controls, we observed increased co-localization of lipid droplets with SQSTM1/p62 and the autophagosome marker LC3B when autophagy flux was blocked (Supplementary Fig. S8G–N). Upon lipolysis inhibition with ATGLi, *APOE4* lipid droplets contained more SQSTM1/p62 and LC3B than *APOE2* lipid droplets (Fig. 5K–N and Supplementary Fig. S8O, P), confirming impaired autophagy upon lipolysis inhibition.

Lastly, to address whether *APOE* genotype differentially impacts lipophagy under conditions of autophagy induction, we analyzed the co-localization of lipid droplets and SQSTM1/p62 or LC3B following acute HBSS-induced starvation. We observed that *APOE4* lipid droplets showed greater co-localization with both SQSTM1/p62 and LC3B than *APOE2* lipid droplets (Fig. 5O–R). This finding suggests that lipophagy in *APOE4* astrocytes is impaired and cannot be activated by starvation-induced autophagy. Given that *APOE2* and *APOE4* astrocytes both accumulate lipid droplets under basal conditions, we hypothesized that prolonged starvation would decrease the number of *APOE2* lipid droplets but not impact *APOE4* lipid droplets (as they are resistant to lipophagy). We observed that *APOE2* astrocytes exhibited a trend towards reduced lipid droplets while *APOE4* astrocytes did not (Supplementary Fig. S8Q). Together, these findings suggest that although both genotypes accumulate significant numbers of lipid droplets, *APOE4* lipid droplets are resistant to lipophagy, unlike *APOE2*. Taken together, we demonstrate that characterizing the *APOE* genotype-specific lipid droplet-associated proteome provides insights on the dynamics of these organelles that can be further validated with cell-based assays.

## Discussion

Lipid droplets are dynamic organelles that impact neurodegenerative disease biology. Lipid droplet proteins are key to specifying their many functions. We defined the proteins and lipids associated with lipid droplets isolated from human iPSC-derived astrocytes homozygous for the *APOE2* (protective), *APOE3* (neutral), and *APOE4 (*risk) genotypes.

Our analyses reveal that human astrocytic lipid droplets exhibit molecular features shared with many cell types and others that are specific to astrocytes. We confirmed the presence of canonical lipid droplet proteins (including PLIN2, PNPLA2(ATGL), ACSL3 and DHRS1) and observed that astrocytic lipid droplet-associated proteins have non-canonical functions like antigen presentation, spliceosome biology, and transcription. Interestingly, a few of these non-canonical functions have been associated with lipid droplets in other organisms like *Drosophila melanogaster*^46,66^ and in other cell systems^3,9^. These findings suggest that human astrocytic lipid droplets have expanded functions beyond simple lipid storage and release. We identified new lipid droplet-associated proteins like RUFY2, a protein involved in endosomal trafficking. RUFY2 contains a PI3P-binding FYVE domain, linking it to lipid droplet metabolism, a connection previously established for other FYVE-containing proteins like DFCP1^67,68^. Genetic knockdown studies show RUFY2 influences lipid droplet morphology and distribution^9,41^, hinting at a potential link between vesicle trafficking defects^69^ and altered lipid biology in astrocytes, particularly in neurodegenerative contexts.

When examining the effects of *APOE* genotype on the composition of lipid droplets, we discovered that the accumulation of lipid droplets is not inherently pathological; rather, their impaired dynamics and turnover distinguishes disease risk from protection. Although both *APOE4* and *APOE2* astrocytes accumulate more lipid droplets than *APOE3*, the molecular features of lipid droplets are different. *APOE2*-associated droplets harbor immune proteins, pointing to potential immunomodulatory roles that may underlie protective biology. This finding mirrors observations in other systems where lipid droplets regulate immune responses^4,70^. *APOE4* lipid droplets accumulate stalled lipophagy adapter proteins and display impaired lipophagy compared to *APOE2* lipid droplets, suggesting that the autophagy-mediated turnover of lipid droplets differentiates risk and protective *APOE* genotypes. Our findings align with a recent study on human data that identified lipophagy genes as dysregulated in AD patients and AD risk^71^. Previous work has linked disruptions to autophagy and lysosomal biology with AD^72–74^. Studies in other model systems have observed impaired autophagy in *APOE4* astrocytes and other glia^16,75,76^. Similarly, reduced lipophagy has been shown to contribute to the progression of AD in disease mouse models^77^. A few studies have suggested potential mechanisms for *APOE4*-mediated autophagy dysregulation, including transcriptional downregulation of autophagy genes like *SQSTM1* and *MAP1LC3B* through direct binding of the promoter regions of these genes^78,79^. Future work will help clarify the mechanisms that link *APOE4* and dysfunctional autophagy in astrocytes. Together with our results, these findings reveal that *APOE* genotype alters lipophagy via changes to the lipid droplet-associated proteome and that lipophagy disruptions could contribute to *APOE4-* associated risk.

*APOE* genotype also impacts lipid saturation within lipid droplets. *APOE2* lipid droplets exhibit higher levels of PUFA-enriched triacylglycerols compared to *APOE4* and *APOE3* lipid droplets. While PUFAs in membrane phospholipids can promote ferroptosis through lipid peroxidation, their sequestration in the triacylglycerol-rich lipid droplet cores mitigates ferroptosis and lipotoxicity^57^. In addition, PUFAs within lipid droplets can be metabolized into lipid signaling mediators in the inflammatory response^57^. We speculate that *APOE2*-mediated protection in astrocytes could arise from their ability to dynamically mobilize lipid droplet lipids to respond to inflammatory stressors implicated in neurodegenerative diseases. The less labile *APOE4* lipid droplets are less able to respond and buffer cellular stress and therefore present a liability to cell health when faced with disease-associated stresses.

Since different *APOE* isoforms were not identified as differentially abundant on lipid droplets, we wondered how these variants were able to alter lipid droplet composition. One possible explanation relates to the localization of APOE to the secretory pathway. Lipid droplets bud from the ER membrane^1,16^. Therefore, perturbations to protein or lipid biology within the ER can be reflected in the contents of lipid droplets. Because APOE isoforms are present in the secretory pathway and are lipid-binding proteins, they could modulate the lipid droplet protein and lipid content via interactions with proteins and lipids in the ER.

A limitation of this study is that oleic acid treatment is an artificial lipid-loading condition and does not correspond to a single defined physiological or pathological stimulus. Oleic acid treatment also reduced intracellular and secreted APOE levels, indicating that lipid loading can alter APOE biology itself. Therefore, our findings should be interpreted in the context of lipid-loaded astrocytes, and future studies will be needed to determine how *APOE* genotype influences lipid droplet composition and dynamics under additional disease-relevant stresses, including inflammatory, oxidative, and amyloid-associated conditions.

There are many other natural extensions of this work for future studies. We have uncovered genotype and cell type-driven diversity in lipid droplet composition. However, our study could not capture heterogeneity within lipid droplets in a single cell type or genotype, which may provide greater insights into organellar function^80,81^. Our study focuses on the cell-autonomous regulation of lipid droplets. Various studies have shown that lipid droplets influence intercellular communication^13,17,61^. In future work, we hope to understand the cell non-autonomous consequences of lipid droplet turnover and in this context, explore whether modulating lipid droplet plasticity can modulate disease risk.

Given the centrality of lipid and glial cell dysregulations contributing to neurodegenerative disease, our study sheds light on the diverse and understudied roles of lipid droplets in human astrocytes. Moreover, understanding *APOE* genotype-driven differences can illuminate the molecular mechanisms underlying the biology of genetic risk and protection for AD.

## Methods

Research in this study conforms with all relevant ethical regulations and institutional guidelines at the NIH Intramural Research Program.

### Human iPSC lines and culture

Isogenic BIONi037-A (APOE3/3), BIONi037-A-2 (APOE2/2) and BIONi037-A-4 (APOE4/4) human iPSC lines (77 year-old female donor) were obtained from the European Bank for induced pluripotent Stem Cells (EBiSC). Isogenic KOLF2.1 J *APOE* derivatives JIPSC001000 (APOE3/3), JIPSC001154 (APOE2/2), JIPSC001150 (APOE4/4) and JIPSC001654 (APOE-KO/KO) (57 year-old male donor) were obtained from The Jackson Lab/iPSC Neurodegenerative Disease Initiative human iPSC collection. All iPSCs were maintained in either mTeSR Plus (STEMCELL Technologies 100-0276, KOLF2.1 J) or Essential 8 Medium (E8; Thermo Fisher Scientific A1517001, BIONi037-A) and plated and expanded on either hESC-Qualified Matrigel Matrix (Corning 354277, KOLF2.1 J) or GelTrex (Thermo Fisher Scientific A1413302, BIONi037-A). Cells were passaged at 90% confluency with either ReLeSR (STEMCELL Technologies 100-0483, KOLF2.1 J) or 1 mM EDTA (Thermo Fisher Scientific 15575-038, BIONi037) in 1X PBS (VWR, 392-0434). Genomic stability of all iPSC lines was characterized by karyotyping or SNP array testing, and all samples were tested to ensure no mycoplasma contamination.

### iPSC differentiation to iPSC-derived astrocytes

Astrocytes were differentiated from iPSCs using small-molecule protocols. For KOLF2.1J-derived astrocytes, neural progenitor cells (NPCs) were generated using STEMdiff SMADi Neural Induction Kit (STEMCELL Technologies 08581) using the kit monolayer protocol. NPCs were then differentiated into astrocytes using human recombinant BMP-4 (Thermo Fisher Scientific 120-05ET) and human recombinant FGF-2 (Thermo Fisher Scientific 100-18B), as previously described^40^. Cells were sorted twice using ACSA-1 APC-conjugated antibodies (Miltenyi Biotec 130-123-555) and frozen at 1 × 10^6^ cells/mL in CryoStor CS10 (STEMCELL Technologies 07930) in cryovials (Azenta Life Sciences 68-1001-11). BIONi037 iPSC lines were differentiated into astrocytes using previously described protocols^30^. Maintenance of all iPSC-derived astrocytes was performed in complete Astrocyte Media (AM; ScienCell Research Laboratories 1801), and cells were passaged at 80–90% confluence using TrypLE Express (Thermo Fisher Scientific 12604039). iPSC-derived astrocytes were cultured in AM media without fetal bovine serum (FBS; ScienCell Research Laboratories 1801) for 4 days before experiments.

### Lipid droplet isolation

iPSC-derived astrocytes were grown in 4 × 150 mm dishes (Corning 353025) until confluent and treated with oleic acid (80 μM, 48 h; MilliporeSigma O3008) or bovine serum albumin (BSA, 0.7%, 48 h; MilliporeSigma A3311) in AM media without FBS. Lipid droplets were isolated by density gradient centrifugation using an adapted protocol^82,83^. Briefly, cells were washed and scraped using a Cell Lifter (Corning 3008) in ice-cold 1X DPBS (Quality Biological 114-057-101), and incubated for 10 min on ice in ice-cold hypotonic lysis medium (HLM; 20 mM Tris-HCl, pH 7.4 and 1 mM EDTA) containing 1X Halt Protease and Phosphatase Inhibitor Cocktail (Thermo Fisher Scientific 78440). Cell suspensions were Dounce homogenized (Wheaton 357542), and lysates were centrifuged at 1000 x *g* for 10 min (Eppendorf 5424 R). The supernatant was diluted to a final concentration of 20% sucrose (MilliporeSigma S0389) in HLM and transferred to the bottom of a 14 mL Ultra-Clear round-bottom tube (Beckman Colter Life Sciences 344060). To create a sucrose gradient, 5 mL of 5% sucrose in HLM were gently layered on top, followed by 4.5–6.5 mL of HLM. Overlaid sucrose gradient samples were centrifuged at 28,000 x *g* for 1 h at 4 °C using an SW-40 Ti swinging bucket rotor (Beckman Coulter Life Sciences 331301) in an ultracentrifuge. Buoyant fractions, which contain isolated lipid droplets, were pipetted from the top 200-500 μL of the resultant gradient. The remaining fractions (cytosolic and ER/mitochondria) were also collected. All fractions were stored at − 80 °C for immunoblotting and mass spectrometry-based proteomics and lipidomics analyses.

### Immunoblotting

The purity of buoyant fractions was analyzed by immunoblotting. Whole cell samples were lysed with 1X RIPA Lysis and Extraction buffer (MilliporeSigma 20-188), and protein concentration was estimated using the bicinchoninic acid (BCA) protein assay (Thermo Fisher Scientific 23227). For SDS-PAGE separation, whole cell protein lysates, and the buoyant, cytosolic and ER/mitochondria fractions were denatured with 1X NuPAGE LDS Sample Buffer (Thermo Fisher Scientific NP0007) and 1X NuPAGE Sample Reducing Agent (Thermo Fisher Scientific NP0009), separated on NuPAGE 4–12% Bis-Tris gels (Thermo Fisher Scientific NP0323BOX) and transferred onto iBlot2 nitrocellulose transfer stacks (Thermo Fisher Scientific IB23002) using the iBlot2 Dry Blotting System (Thermo Fisher Scientific IB21001). Membranes were blocked with Intercept TBS blocking buffer (LICORbio 927-60001) for 1 h at room temperature, and probed overnight with primary antibodies (Supplementary Data 27) at 4 °C. After washing with 1X TBS (KD Medical RGF-3385) containing 0.1% (v/v) Tween 20 (MilliporeSigma P9416), membranes were incubated with horseradish peroxidase (HRP)-conjugated secondary antibodies (1:10,000; Supplementary Data 27) for 1 h at room temperature. Immunoblots were developed with SuperSignal West Pico Chemiluminescent Substrate (Thermo Fisher Scientific 34580) and imaged on an Amersham Imager 680 (Cytiva).

To measure total secreted proteins in astrocyte-conditioned culture media after vehicle or oleic acid treatments, proteins from raw FBS-free media samples were separated by SDS-PAGE and transferred onto nitrocellulose membranes as described above. Membranes were stained with Revert 700 Total Protein Stain (LI-COR Biosciences 926-11015) for 5 min at room temperature and rinsed twice with ultrapure water before imaging.

### APOE ELISA assay

iPSC-derived astrocytes were grown on 96-well μ-Plates (Ibidi 89626) and cultured with oleic acid (80 μM) or vehicle control (0.7% BSA) diluted in AM media without FBS for 48 h. Conditioned media was collected, and APOE content was measured using a Human Apolipoprotein E ELISA kit (Thermo Fisher Scientific EHAPOE) as per the manufacturer’s instructions. An EnSpire plate reader (PerkinElmer) was used to read absorbance. APOE secreted levels were normalized to total secreted protein levels quantified by immunoblotting in the same conditioned media samples.

### Proteomics sample preparation

Sample preparation for proteomics was conducted on a fully automated workflow as previously described^84^. Briefly, 200 μL lysis buffer (50 mM Tris-HCI (Thermo Fisher Scientific 15568025), 50 mM NaCl (ThermoFisher 24740011), 1% SDS (Thermo Fisher Scientific 15553027), 1% Triton X-100 (MilliporeSigma T8787), 1% NP-40 (Thermo Fisher Scientific 85124), 1% Tween-20 (MilliporeSigma P9416), 1% glycerol (MP Biomedicals 800687), 1% sodium deoxycholate (wt/vol; MilliporeSigma D6750), 5 mM EDTA (Thermo Fisher Scientific 15575020), 5 mM dithiothreitol (DTT; Thermo Fisher Scientific 20290), 5KU benzonase (MilliporeSigma E8263), and 1 × complete protease inhibitor (MilliporeSigma 5892970001)) was added to the samples. The lysates were incubated at 65 °C for 30 min at 1200 rpm for protein denaturation. Samples were then alkylated with 10 mM iodoacetamide (Thermo Fisher Scientific A39271) for 30 min at room temperature. The protein concentration was determined using DC Protein Assay (Bio-Rad 5000111). The protein enrichment and on-bead (MilliporeSigma GE45152105050250, GE665152105050250) tryptic/Lys-C (Promega X100) digestion were conducted using a KingFisher APEX robotic system. Proteins were digested with Trypsin/Lys-C mix (Promega V5073) in 50 mM ammonium bicarbonate (MilliporeSigma A6141) at 37 °C for 18 h. Following digestion, peptides were vacuum-dried and reconstituted in 2% acetonitrile (ACN, Thermo Fisher Scientific TS-51101) with 0.1% formic acid (Thermo Scientific 28905). 1 μg of peptides was used for LC-MS/MS proteomic analysis.

### Liquid chromatography and mass spectrometry

For each sample, digested peptides were analyzed using an UltiMate 3000 nano-HPLC system (Thermo Fisher Scientific) coupled with an Orbitrap Eclipse mass spectrometer (Thermo Fisher Scientific). The peptides were loaded at 5 μL/min onto a trap column (75 μm × 2 cm, PepMap nanoViper C18 column, 3μm, 100 Å, Thermo Fisher Scientific) equilibrated in 2% ACN with 0.1% trifluoroacetic acid (TFA, Thermo Scientific 85183). The samples were loaded to the trap column for 5 min and switched in-line with ES903A nano C18 column (75 μm × 500 mm, 2 μm, 100 Å,Thermo Scientific) using an 80-minute linear gradient of 2–40% phase B (5% DMSO in 0.1% formic acid, in ACN). The column temperature was maintained at 60 °C, and the flow rate was set to 300 nL/min. Data were acquired in data-independent acquisition (DIA) mode. The MS1 scan was set at a resolution of 120,000, with a standard AGC target, and the maximum injection time was set to auto. The MS2 scans covered a precursor mass range of 400–1000 m/z, using an isolation window of 8 m/z with 1 m/z overlap, resulting in a total of 75 windows per scan cycle. Fragmentation was performed using high-energy collisional dissociation (HCD) with a normalized collision energy of 30%. MS2 spectra were acquired at a resolution of 30,000, with a scan range of 145–1450 m/z, and the loop control was set to 3 s.

### Proteomics data analysis

We performed DIA database searches using Spectronaut (version 19, Biognosys) with the directDIA search strategy. We used the UniProt human proteome reference with reviewed genes (20,384 entries) as the library FASTA file. directDIA false discovery rate set to 1%, protein N-terminal acetylation and methionine oxidation were set as variable modifications and carbamidomethylation of cysteine residues was selected as a fixed modification. We used MS2 peak areas of each peptide for quantification. Medium normalization was applied to all samples.

### Bioinformatic analysis of the iPSC-derived astrocyte lipid droplet-associated proteome

For all samples, we determined how many peptides were detected for each protein. Proteins only identified with a single peptide were excluded from analysis. Spectronaut outputs of each fraction (cytosolic and buoyant) were subjected to filtering, normalization, and imputation using the DEP2 package in R^85^. Proteins present in each replicate of any genotype-treatment group across both lines were retained, then values were normalized using variance stabilizing normalization^86^. Imputation was carried out using a mixed imputation model where imputation for proteins with randomly missing values were imputed using kNN, and proteins not missing at random (defined as proteins missing in every replicate of a genotype-treatment group) were imputed as 1.

To determine differential abundance, statistical analysis was conducted using the DEP2 package^85^ which utilizes limma and fdrtools. To identify proteins differentially abundant between oleic acid and vehicle (BSA) loading control treated cells, comparisons of all oleic acid samples to all vehicle samples from the buoyant fraction across both lines were assessed. Proteins were identified as differentially abundant if they met a p-value cutoff of < 0.1 (Supplementary Data 2). To determine proteins that were differentially abundant between genotypes, buoyant fraction oleic acid samples were compared, with proteins identified as differentially abundant with an adjusted *p*-value threshold of < 0.1. This higher p-value allows for the exclusion of false negatives and accounts for the variability due to cell lines from different humans. It also allows us to comprehensively account for changes between oleic acid and vehicle (BSA) treatments.

To identify proteins that were enriched in the lipid droplet fraction compared to the cytosolic fraction, all proteome lists were filtered for common mass spectrometry contaminants using a published database^42^. Proteins that appeared in over half of the mass spectrometry studies in the database (Supplementary Data 1) were excluded from our analyses. Z-score transformations were applied to the cytosolic and buoyant fraction datasets, and a mean z-score of oleic acid-treated samples was calculated for each protein identified in the fractions. A linear model was fitted to the mean z-scores of the buoyant and cytosolic fractions. Only complete cases were considered. Residuals were calculated for each protein detected in the buoyant fraction. Residual scores were then ranked to determine proteins enriched in the buoyant fraction. The top 2.5% of residual scores over 0 were chosen for each comparison (Supplementary Data 5). To identify proteins that were only present in the buoyant fraction samples, we ranked the proteins by mean z-score from greatest to least and selected proteins with a z-score of >−1 (Supplementary Data 6). This exact process was repeated to identify proteins enriched in a single *APOE* genotype by calculating a mean z-score and ranked residual value considering samples from each genotype separately (Supplementary Data 10).

### Lipidomics sample preparation and analysis

Comprehensive targeted lipidomics was accomplished using a flow-injection assay based on lipid class separation by differential mobility spectroscopy and selective multiple reaction monitoring (MRM) targeting individual lipid species (Lipidyzer platform). A detailed description of lipid extraction, employed software, and the quantitative nature of the approach can be found elsewhere^87–89^. Briefly, after the addition of > 60 deuterated internal standards, lipids were extracted using methyl tert-butyl ether. Organic extracts were combined and subsequently dried under a gentle stream of nitrogen and reconstituted in running buffer. Lipids were then analyzed using flow-injection in MRM mode employing a Shimadzu Nexera series HPLC and a Sciex QTrap 6500+ mass spectrometer. For further analysis, lipidomic data normalized to cell number or sample volume were used. Specifically, 25 μL of lipid droplet isolations or cell pellets were used. SODA light software was used for data visualization^30^.

### Human transcriptome data analysis

We downloaded four published single-nuclei RNA-seq datasets (Mathys et al. 2023; [syn52293432], Morabito et al. 2021; syn22079621], Hawrylycz et al. 2024; [syn26273710 and syn51792375], and Wang et al. 2024; [syn51753326]), reporting frontal cortex Alzheimer’s disease (AD) and control cases^51–54^. We used all samples with *APOE* genotype 2/2, 2/3, 3/3, 3/4, 4/4, 2/4 (Supplementary Data 14). Cases were selected based on clinical and neuropathological diagnosis as follows: (1) Mathys et al. 2023, Control: basic > cogdx: NCI = 1 and, basic > niareagansc: No AD = 4 or low = 3; AD: basic > cogdx: AD = 4 and basic > niareagansc: intermediate = 2 or high = 1. (2) Morabito et al. 2021, Control: diagnosis = Control and, Neuropath.Dx.1 = Normal; AD: Diagnosis = AD and Neuropath.Dx.1 = AD. (3) Hawrylycz et al. 2024, Consensus Clinical dx = Control and, ADNC = Not AD or low; AD: Consensus Clinical dx = AD and ADNC = intermediate or high. (4) Wang et al. 2024, Control: = Control = Yes and, NIA-AA = Not AD or Low ADNC; AD: AD = Yes and NIA-AA = Intermediate or High.

Samples were iIntegrated based on SampleID using mnc correct (SnapATAC2)^90^ and astrocytes were identified and extracted from each dataset using astrocytic marker genes (*SLC1A2*, *AQP4*, *ALDH1L1*) as described before^91^. Calculated log-normalized pseudobulk counts for each genotype (*APOE2*-carrier = 2/2 and 2/3, *APOE3*-homozygous = 3/3, *APOE4*-carriers = 3/4 and 4/4) were used for violin plots, and centered values were used for heatmaps.

### Immunofluorescence microscopy and 3D co-localization analysis

To quantify the number of lipid droplets in iPSC-derived astrocytes under lipid droplet formation conditions, cells were plated on 96-well μ-Plates (Ibidi 89626) and cultured with oleic acid (80 μM) or vehicle control (0.7% BSA) diluted in AM media without FBS. After 48 h, cells were rinsed with DPBS and fixed with 4% paraformaldehyde (PFA; Electron Microscopy Sciences 15710-S). Lipid droplets were labeled with LipidSpot 610 stain (1:1,000; Biotium 70069) as per manufacturer’s instructions. Cell membranes and nuclei were counterstained with MemBrite Fix 568/580 cell surface stain (1:5000; Biotium 30095) and Hoechst 33258 (1:10,000; Thermo Fisher Scientific H3570), respectively. Images were acquired using a Nikon CSU-W1 Spinning Disk microscope with a 40X water immersion objective lens (NA 1.25). Z-stacks with a stack height of 0.5 μm were acquired, and images were analyzed using the Nikon NIS-Elements General Analysis (GA3) suite.

To determine lipid droplet co-localization with canonical and new lipid droplet-associated proteins, iPSC-derived astrocytes were treated with oleic acid (80 μM, 48 h) in AM media without FBS and fixed with 4% PFA. After fixation, cells were permeabilized with 0.01% saponin (MilliporeSigma SAE0073) in DPBS for 10 min, or with 0.3% Triton X-100 in DPBS for 5 min. Following permeabilization, cells were blocked for 1 h at room temperature with 5% BSA, 1% normal donkey serum (MilliporeSigma S30-M), 0.01% saponin in DPBS (for samples permeabilized with saponin) or 5% BSA, 1% normal donkey serum in DPBS (for samples permeabilized with Triton X-100). Primary antibodies (Supplementary Data 27) diluted in the respective blocking buffers were incubated overnight at 4 °C. Cells were washed with DPBS, and secondary antibodies (1:2,000; Supplementary Data 27) were diluted in the respective blocking buffers and incubated for 1 h at room temperature. After DPBS washes, cell nuclei were counterstained with Hoechst 33258 (1:10,000) and lipid droplets labeled with LipidSpot 610 (1:1,000). Cells were imaged immediately after immunofluorescence staining using a Nikon CSU-W1 Spinning Disk microscope with a Nikon SR HR Plan Apo Lambda S 100X silicone immersion objective lens (NA 1.35). For co-localization analyses in 3D, Z-stacks with 0.2 μm-thick slices were acquired in 1024 × 1024 mode in the Nikon software NIS-Elements. The 3D Volume View tool on Nikon’s GA3 in NIS-Elements was used for 3D rendering and segmentation of lipid droplets and proteins of interest (*i.e*., RUFY2, HSD17B8, DHRSX, SQSTM1/p62 and LC3B). The lipid droplet volume interacting with the protein volume was extracted and divided over the total lipid droplet volume quantified in each image.

### BODIPY-C_12_ pulse-chase assay at baseline

iPSC-derived astrocytes plated on 96-well μ-Plates (Ibidi 89626) were labeled for 2 h with 0.5 μM BODIPY FL C_12_ lipid (Thermo Fisher Scientific D3822) diluted in AM media without FBS. Cells were then washed three times and incubated 2 h in AM media without FBS to allow for the fluorescent lipids to incorporate into lipid droplets. Following this incubation, cells were chased in AM media without FBS and live imaged at 18 h and 24 h timepoints. Lipid droplets were labeled with LipidSpot (1:1000; Biotium 70069), and cytosol was labeled with ViaFluor dye (1:5000; Biotium 30068). Both dyes were present across the chase period. Z-stack images were acquired using a Nikon CSU-W1 Spinning Disk microscope with a 40X water immersion objective lens (NA 1.25).

### Treatment with lipolysis and lipophagy inhibitors

To isolate lipid droplet degradation by lipophagy in our lipid droplet turnover experiments, we chemically inhibited lipolysis. We used the ATGL (PNPLA2) lipase inhibitor atglistatin (ATGLi; Sigma-Aldrich SML1075) at 10 μM for 72 h and 96 h. To inhibit lipophagy, we used bafilomycin A1 (STEMCELL Technologies 74242) at 10 nM for 24 h, and chloroquine (Thermo Fisher Scientific P36241) at 10 μM for 24 h. All inhibitors were diluted in AM media without FBS. Vehicle control treatments (DMSO for ATGLi and bafilomycin A1; ultrapure H_2_0 for chloroquine) were included for comparison. For analysis of lipid droplet co-localization with autophagy adapter proteins under lipolysis inhibition conditions, cells were treated with ATGLi 10 μM for 96 h and fixed for immunofluorescence analysis as described above (Supplementary Data 27).

### BODIPY-C_12_ pulse-chase assay with ATGLi

iPSC-derived astrocytes were pre-treated with ATGLi 10 μM or vehicle control (DMSO) for 48 h. Cells were then pulsed with 0.5 μM BODIPY FL C_12_ lipid (Thermo Fisher Scientific D3822) diluted in AM media without FBS for 2 h and washed three times with fresh media. Lipid droplets and cytosol were labeled with LipidSpot (1:1000; Biotium 70069) and ViaFluor dye (1:5000; Biotium 30068), respectively. Conditioned media containing ATGLi or vehicle were added back to cells to start the chase and kept throughout the chase period. Cells were live imaged at 18 h, 24 h and 42 h chase timepoints, which corresponded to 72 h (for 18 h and 24 h of chase) and 96 h (for 42 h of chase) of drug treatment. Z-stack images were acquired using a Nikon CSU-W1 Spinning Disk microscope with a 40X water immersion objective lens (NA 1.25).

### LipiDye^TM^-M staining and analysis

Following the chemical inhibition of lipid droplet degradation pathways, the lipid metabolism state of iPSC-derived astrocytes was measured using LipiDye^TM^-M (Funakoshi FDV-0028), a C_12_-fatty acid-based metabolic tracer. After removing conditioned media containing drugs, LipiDye^TM^-M was added to cells at 5 μM in Live Cell Imaging Solution (Thermo Fisher Scientific A59688DJ) and incubated for 3 h at 37 °C. Cells were then live imaged in the green and red channels using a Nikon CSU-W1 Spinning Disk microscope with a 40X water immersion objective lens (NA 1.25) in 1024 × 1024 mode. Z-stacks with a height of 0.5 μm were acquired. For ratiometric analysis of pixel intensities, we used NIS-Elements to calculate the Green/Red intensity ratio of pixels contained within the cytosolic areas of each individual slice of the Z-stack. Ratio values were max-normalized and plotted as cumulative distributions. A Kolmogorov-Smirnov (K-S) test was used to determine the statistical difference between conditions. The *K-S* statistic and *P* value are reported for each comparison.

### Starvation experiments

To assess lipid droplet dynamics under autophagy-inducing conditions (starvation), astrocytes were starved by applying HBSS (Thermo Fisher Scientific 14025082) as cell culture medium for 1 h. Cells were then fixed for immunofluorescence analysis (Supplementary Data 27). To measure lipid droplet accumulation in the context of prolonged starvation, we cultured astrocytes in AM media containing FBS without media changes for 5 days (Supplementary Fig. S8Q).

### Statistics and reproducibility

Details of analyses can be found in the figure legends. All statistical analysis for image quantification and functional assays was performed using GraphPad Prism, Python and R Studio. No statistical method was used to predetermine sample size, but sample sizes were based on previously published research on iPSC-derived astrocytes. No data were excluded from analysis. Investigators preparing samples were different from those running molecular characterization and those analyzing data for all omics data. No data was excluded from analysis.

### Reporting summary

Further information on research design is available in the Nature Portfolio Reporting Summary linked to this article.

## Supplementary information

Supplementary Information

Transparent Peer Review file

Reporting Summary

Description of Additional Supplementary Files

Supplementary Data 1-27

## Source data

Source Data

## Data Availability

The mass spectrometry proteomics data generated in this study have been deposited to the ProteomeXchange Consortium via the PRIDE^92^ partner repository (https://www.ebi.ac.uk/pride/archive/projects/PXD067161) with the dataset identifier PXD067161. Processed proteomics are available in supplementary data files. Processed lipid droplet lipidomics data are available via the Neurolipid Atlas website (https://neurolipidatlas.com/), and full data tables are included in supplementary data files. Source data for other plots are provided as a Source Data file. Source data are provided in this paper.
