# Supplementary material for: APOE genotypes differentially remodel the astrocytic lipid droplet proteome to shape lipid droplet dynamics
Source: Nat Commun. 2026 Aug 19;17:9900. doi: 10.1038/s41467-026-76565-6 (PMC13582944; doi:10.1038/s41467-026-76565-6)
Supplement: Supplementary file 1 [file 41467_2026_76565_MOESM1_ESM.pdf]

Supplementary Figures and Legends for
"*APOE* genotypes differentially remodel the astrocytic lipid droplet proteome to shape lipid droplet dynamics"

Cuní-López et al.

Figure S1 - Cuní-López et. al

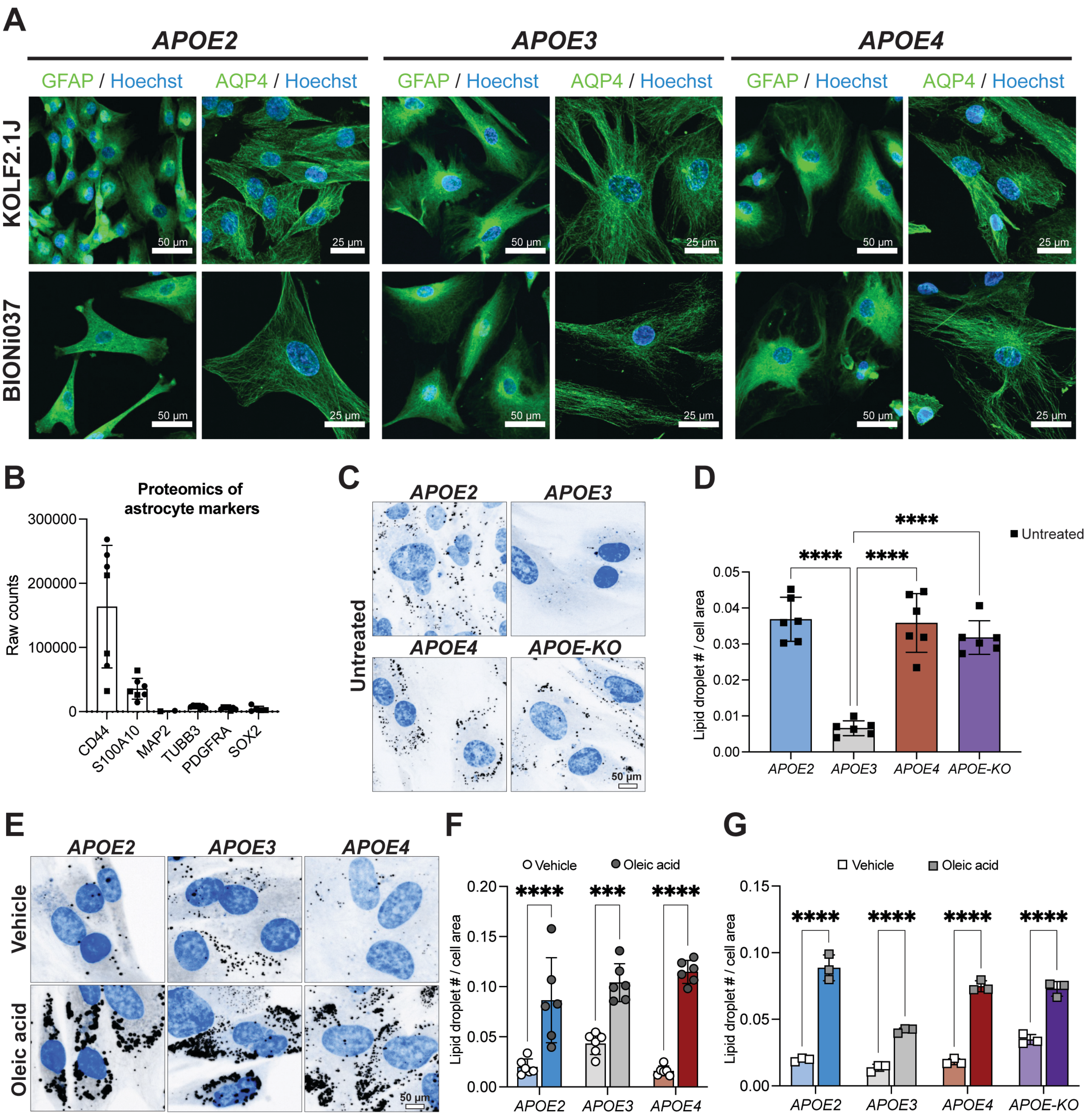

Figure S1. Characterization of lipid droplets in iPSC-derived astrocytes

A. iPSC-derived astrocytes from KOLF2.1J and BIONi037A isogenic series stain for canonical astrocytic markers GFAP and AQP4. Scale bars: AQP4, 25 µm; GFAP, 50 µm. **B.** Raw abundance counts of astrocyte markers CD44 and S100A10 and non-astrocytic markers for neurons (MAP2, TUBB3), oligodendrocytes (PDGFRA), and iPSCs (SOX2), measured by whole-cell proteomics. Squares represent KOLF2.1J-derived lines and circles represent BIONi037A-derived lines. Each point represents an independent line and derivation, n = 3–4. **C.** Neutral lipid immunofluorescence in untreated KOLF2.1J iPSC-derived astrocytes homozygous for *APOE2*, *APOE3*, *APOE4*, or *APOE*-KO. LipidSpot is shown in black and Hoechst 33258-stained nuclei in blue. Scale bar, 50 µm. **D.** Quantification of lipid droplets per cell area in untreated *APOE2, APOE3, APOE4*, and *APOE-KO* KOLF2.1J astrocytes. Each point represents n= 6 imaging frames; data are mean ± SD. ****p ≤ 0.0001 by one-way ANOVA with Tukey's test. **E.** Neutral lipid immunofluorescence in BIONi037A astrocytes homozygous for *APOE2*, *APOE3*, or *APOE4* and treated with vehicle (BSA) or 80 µM oleic acid. LipidSpot is shown in black and Hoechst 33258-stained nuclei in blue. Scale bar, 50 µm. **F.** Quantification of lipid droplets per cell area in BIONi037A astrocytes across *APOE* genotypes and treatments. Data represent n = 6 independent treatments; mean ± SD. ***p ≤ 0.001, ****p ≤ 0.0001 by two-way ANOVA with Šídák's test. **G.** Quantification of lipid droplets per cell area in KOLF2.1J *APOE2*, *APOE3*, *APOE4*, and *APOE*-KO astrocytes treated with vehicle or 80 µM oleic acid. Each point represents n = 6 independent treatments; mean ± SD. ****p ≤ 0.0001 by two-way ANOVA with Šídák's test.

Figure S2 - Cuní-López et. al

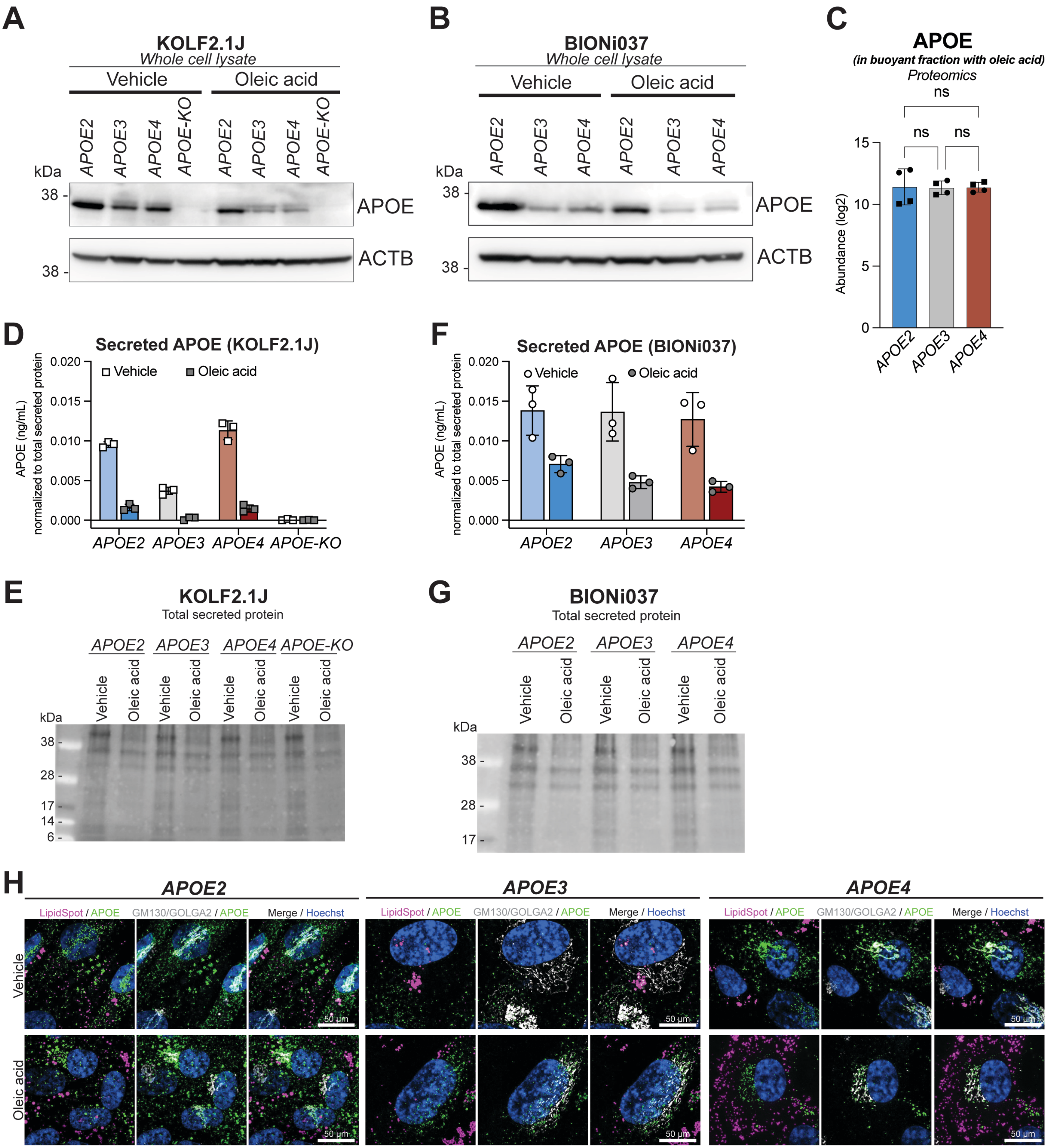

Figure S2. Characterization of APOE protein levels and localization in iPSC-derived astrocytes

A. Western blot of APOE in whole-cell lysates from KOLF2.1J iPSC-derived astrocytes harboring *APOE2*, *APOE3*, *APOE4*, or *APOE*-KO genotypes and treated with vehicle (BSA) or 80 μM oleic acid. β-actin (ACTB) was used as a loading control. **B.** Western blot of APOE in whole-cell lysates from BIONi037A astrocytes harboring *APOE2*, *APOE3*, or *APOE4* and treated with vehicle or oleic acid. ACTB was used as a loading control. **C.** APOE abundance in buoyant fractions across common *APOE* genotypes by mass spectrometry. Data represent n = 4 independent replicates across two isogenic human iPSC-derived astrocyte sets; mean ± SD. **D.** APOE secretion into conditioned media after vehicle or oleic acid treatment in KOLF2.1J *APOE2*, *APOE3*, *APOE4*, and *APOE*-KO astrocytes. APOE concentration was normalized to total secreted protein shown in E. Data represent n = 3 independent treatments; mean ± SD. **E.** Total protein stain of conditioned media from KOLF2.1J *APOE2*, *APOE3*, *APOE4*, and *APOE*-KO astrocytes treated with vehicle or oleic acid. **F.** APOE secretion into conditioned media after vehicle or oleic acid treatment in BIONi037A *APOE2*, *APOE3*, and *APOE4* astrocytes. APOE concentration was normalized to total secreted protein shown in G. Data represent n = 3 independent treatments; mean ± SD. **G.** Total protein stain of conditioned media from BIONi037A *APOE2*, *APOE3*, and *APOE4* astrocytes treated with vehicle or oleic acid. **H.** Immunofluorescence of APOE in green, LipidSpot-positive lipid droplets in magenta, Golgi marker GM130/GOLGA2 in gray, and Hoechst-stained nuclei in blue in KOLF2.1J *APOE2*, *APOE3*, and *APOE4* astrocytes. Scale bars, 50 μm.

Figure S3 - Cuní-López et. al

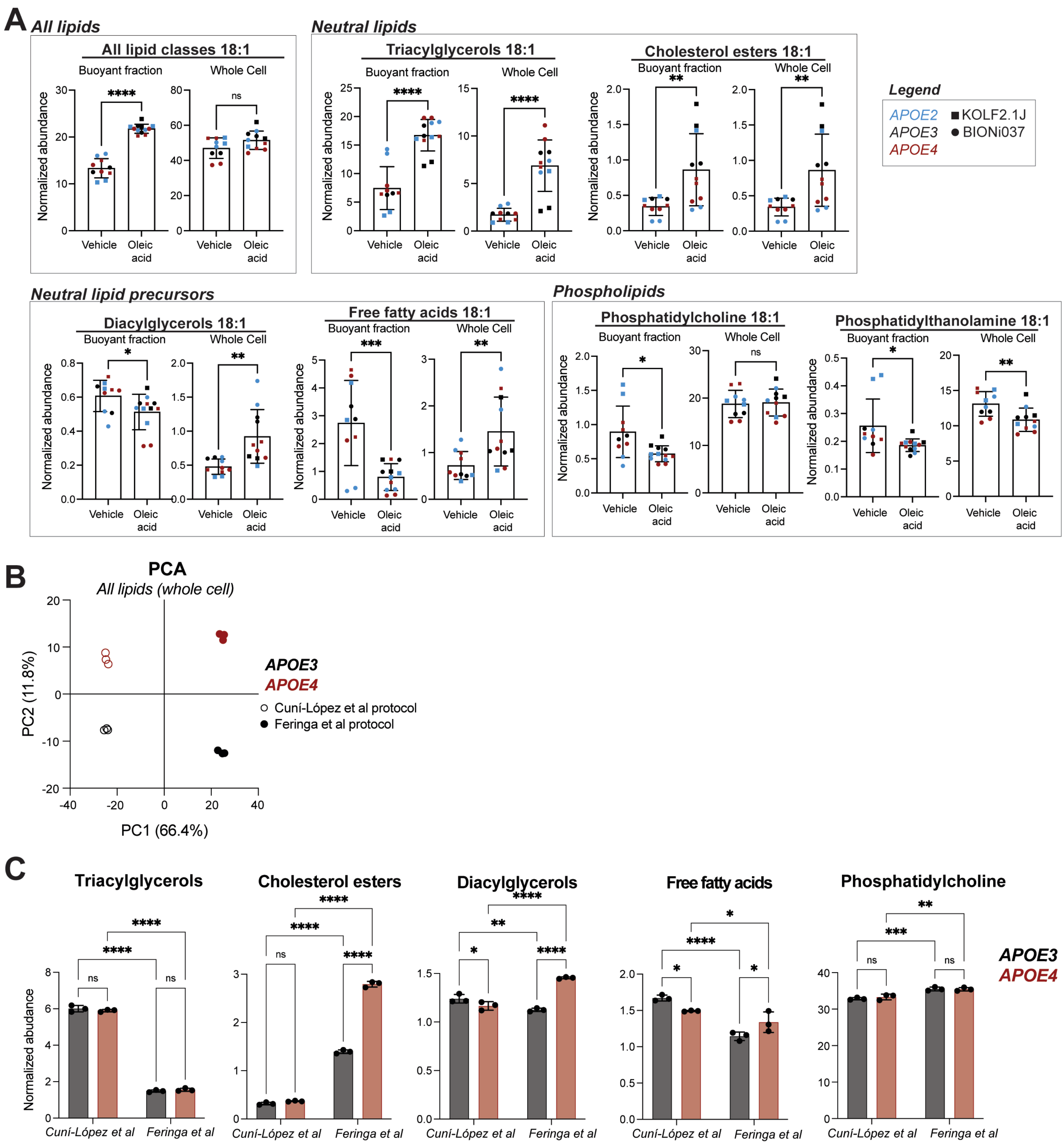

Figure S3. Characterization of cellular lipid state upon oleic acid treatment and under different cell culture conditions

A. Abundance of oleic acid (18:1)-containing lipid classes in buoyant fractions and unfractionated whole-cell samples from iPSC-derived astrocytes of different *APOE* genotypes treated with vehicle (BSA) or 80 μM oleic acid. Lipid classes include neutral lipids (triacylglycerols and cholesterol esters), neutral lipid precursors (diacylglycerols and free oleic acid), and phospholipids (phosphatidylcholine and phosphatidylethanolamine). Data represent n = 10–12 buoyant-fraction and n = 10–11 whole-cell independent replicates across two isogenic human iPSC-derived astrocyte sets. *$p \leq 0.05$, **$p \leq 0.01$, ***$p \leq 0.001$, ****$p \leq 0.0001$ by unpaired t-test. **B.** PCA plot of lipidomics signatures from *APOE3* and *APOE4* BIONi037A iPSC-derived astrocytes cultured with BSA using either a 4-day FBS withdrawal period, following the Cuní-López et al. protocol, or a 1-day FBS withdrawal period, following the Feringa et al. protocol. Data represent n = 3 independent experiments. **C.** Abundance of triacylglycerols, cholesterol esters, diacylglycerols, free fatty acids, and phosphatidylcholine in *APOE3* and *APOE4* BIONi037A astrocytes cultured using the Cuní-López et al. or Feringa et al. protocol. Data represent n = 3 independent experiments. *$p \leq 0.05$, **$p \leq 0.01$, ***$p \leq 0.001$, ****$p \leq 0.0001$ by two-way ANOVA with uncorrected Fisher's LSD.

Figure S4 - Cuní-López et. al

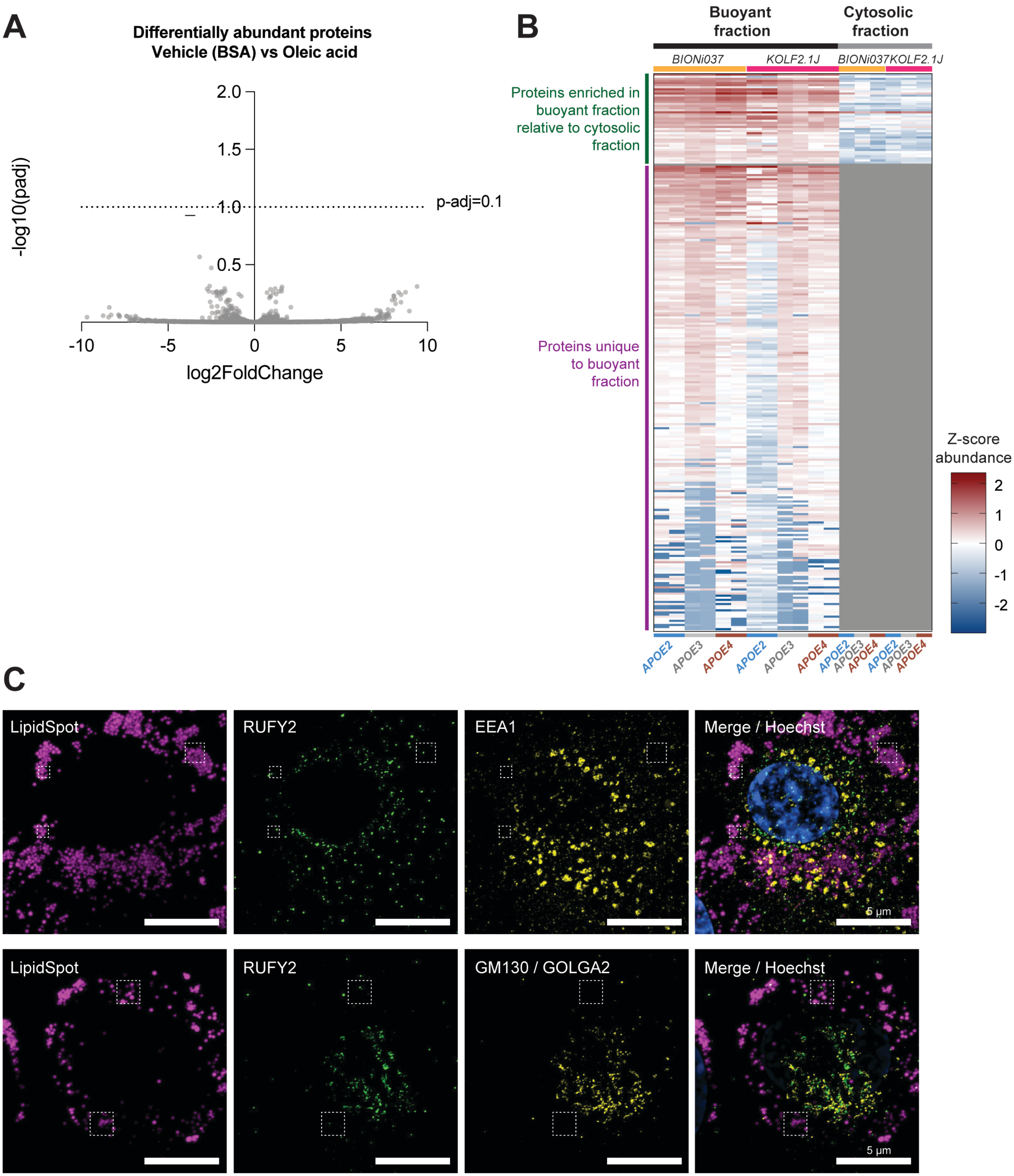

Figure S4. Characterization of the astrocyte lipid droplet proteome
A. Volcano plot of differentially abundant proteins in buoyant fractions from vehicle (BSA)- and oleic acid-treated iPSC-derived astrocytes. Dashed line indicates -log10(p-adj) = 0.1. **B.** Heatmap of mass spectrometry proteomics data for proteins included in the human iPSC-derived astrocyte lipid droplet-associated proteome across buoyant fractions (n = 10) and cytosolic fractions (n = 6). Values represent z-scored abundance. **C.** Immunofluorescence of RUFY2 in green, LipidSpot-positive lipid droplets in magenta, early endosomes marked by EEA1 in yellow, Golgi marked by GM130/GOLGA2 in yellow, and Hoechst-stained nuclei in blue. Dashed squares indicate regions where RUFY2 localizes to lipid droplets but not early endosomes or Golgi. Scale bars, 5 µm.

Figure S5 - Cuní-López et. al

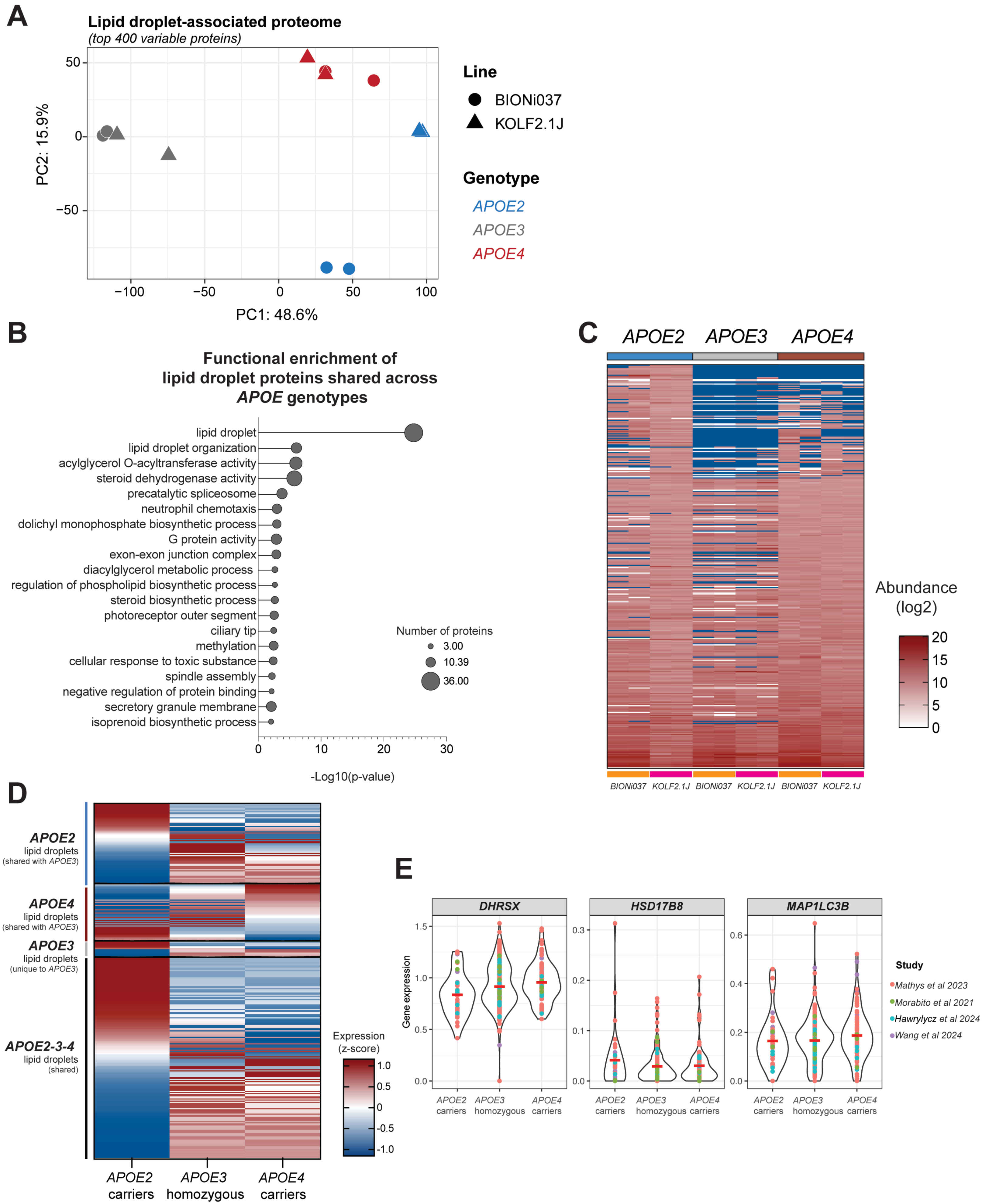

Figure S5. Characterization of the effects of *APOE* genotype on lipid droplet proteins

A. Principal component analysis of lipid droplet-associated proteomes identified in *APOE2*, *APOE3*, and *APOE4* iPSC-derived astrocytes from BIONi037A and KOLF2.1J parental lines. **B.** Overrepresentation analysis of lipid droplet-associated proteins common to all three *APOE* genotypes. **C.** Heatmap of mass spectrometry proteomics data for proteins significantly differentially abundant in lipid droplets from iPSC-derived astrocytes across *APOE* genotypes. Values represent z-scored abundance. **D.** Heatmap of astrocyte gene expression across four human single-nucleus RNA-seq datasets. Values represent z-scores of centered, log-normalized pseudobulked counts for transcripts corresponding to lipid droplet-associated proteins identified by iPSC-derived astrocyte lipid droplet proteomics across *APOE* genotypes. **E.** Violin plots showing expression of selected lipid droplet protein-encoding genes that display differential abundance in iPSC-derived astrocytes across *APOE* genotypes. Each point represents transcript expression values from *APOE* carrier individuals selected in each study.

Figure S6 - Cuní-López et. al

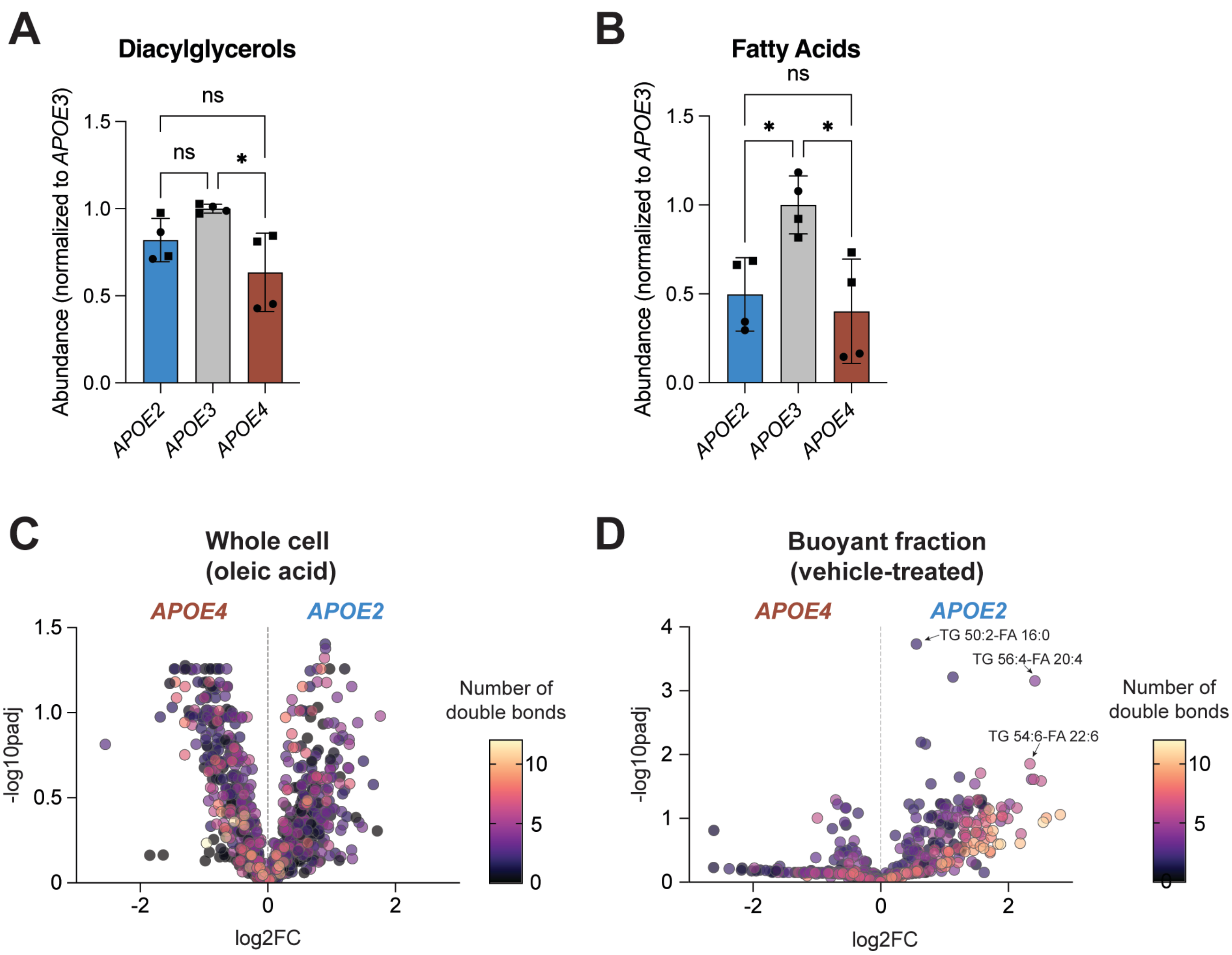

Figure S6. Characterization of the effects of *APOE* genotype on lipid droplet lipids
A. Diacylglycerol abundance in buoyant fractions across *APOE* genotypes from oleic acid-treated iPSC-derived astrocytes. Data represent n = 4 independent replicates across two isogenic human iPSC-derived astrocyte sets. *$p \leq 0.05$ by one-way ANOVA with Tukey's test. **B.** Free fatty acid abundance in buoyant fractions across *APOE* genotypes from oleic acid-treated astrocytes. Data represent n = 4 independent replicates across two isogenic human iPSC-derived astrocyte sets. *$p \leq 0.05$ by one-way ANOVA with Tukey's test. **C.** Volcano plots comparing whole-cell lipids between *APOE4* and *APOE2* astrocytes after oleic acid treatment. Each point represents an individual lipid species from n = 4 independent replicates across two isogenic astrocyte sets. Color indicates fatty acid saturation, shown as number of double bonds. **D.** Volcano plots comparing buoyant-fraction lipids between *APOE4* and *APOE2* astrocytes after vehicle (BSA) treatment. Each point represents an individual lipid species from n = 4 independent replicates across two isogenic astrocyte sets. Color indicates fatty acid saturation, shown as number of double bonds.

Figure S7 - Cuní-López et. al

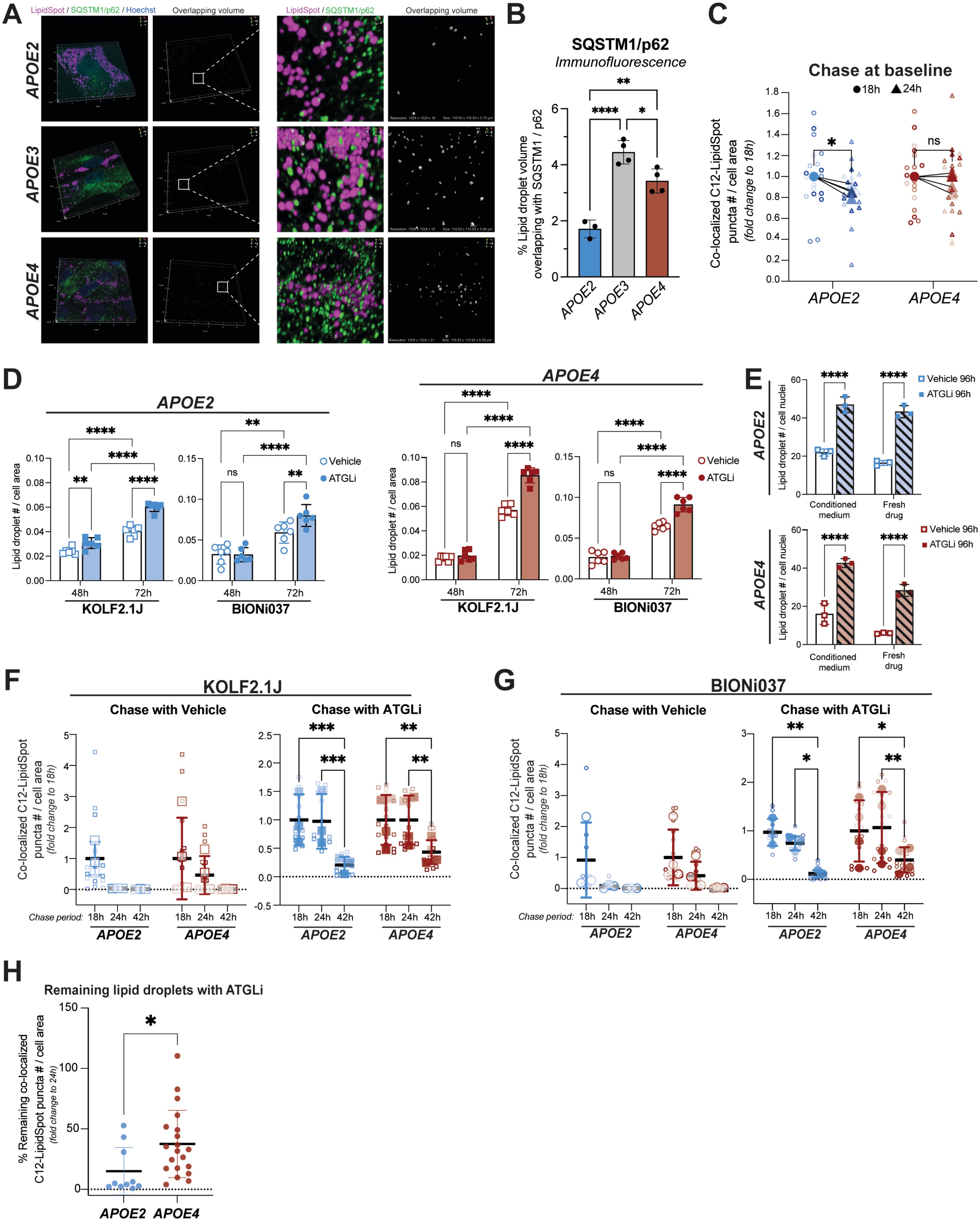

Figure S7. Characterization of lipid droplet dynamics

A. Immunofluorescence of SQSTM1/p62 in green contacting LipidSpot-positive lipid droplets in magenta in BIONi037A iPSC-derived astrocytes across *APOE* genotypes. Overlap volume is white. Representative high-magnification fields do not reflect normalized lipid droplet numbers across genotypes. 3D projection: 111 × 111 × 5.40–6.30 μm^3. **B.** Percent lipid droplet volume overlapping SQSTM1/p62 signal in BIONi037A astrocytes by 3D confocal imaging. Data represent approximately 700 lipid droplets per frame from 10–20 cells per biological replicate; n = 3–4 frames per *APOE* genotype; mean ± SD. ****$p \leq 0.0001$, **$p \leq 0.01$, *$p \leq 0.05$ by one-way ANOVA with Tukey's test. **C.** Change in BODIPY-C12/LipidSpot-positive lipid droplets during an 18–24 h chase under baseline conditions in BIONi037A *APOE2* and *APOE4* astrocytes. Large symbols show n = 4 independent pulse-chase experiments normalized to 18 h; small symbols show individual replicates. *$p \leq 0.05$ by repeated-measures two-way ANOVA with Šídák's test. **D.** Lipid droplet number per cell area after ATGLi or vehicle treatment for 48 h and 72 h in KOLF2.1J and BIONi037A *APOE2* and *APOE4* astrocytes. Data represent n = 6 independent treatments. ****$p \leq 0.0001$, **$p \leq 0.01$ by repeated-measures two-way ANOVA with uncorrected Fisher's LSD. **E.** Lipid droplet number per cell area after 96 h ATGLi treatment in KOLF2.1J *APOE2* and *APOE4* astrocytes, with ATGLi maintained throughout or freshly re-added 24 h before endpoint. Data represent n = 3 independent treatments. ****$p \leq 0.0001$ by two-way ANOVA with Šídák's test. **F–G.** BODIPY-C12/LipidSpot-positive lipid droplets per cell area at 18 h, 24 h, and 42 h chase with vehicle or ATGLi in KOLF2.1J and BIONi037A *APOE2* and *APOE4* astrocytes. Large symbols show n = 3–4 independent experiments normalized to 18 h; small symbols show individual replicates. Significance was tested by two-way ANOVA with Tukey's test. **H.** Remaining BODIPY-C12/LipidSpot-positive lipid droplets after 42 h chase with ATGLi in BIONi037A *APOE2* and *APOE4* astrocytes, normalized to 24 h. Data represent n = 10–20 replicates from independent pulse-chase experiments. *$p \leq 0.05$ by unpaired t-test.

Figure S8 - Cuní-López et. al

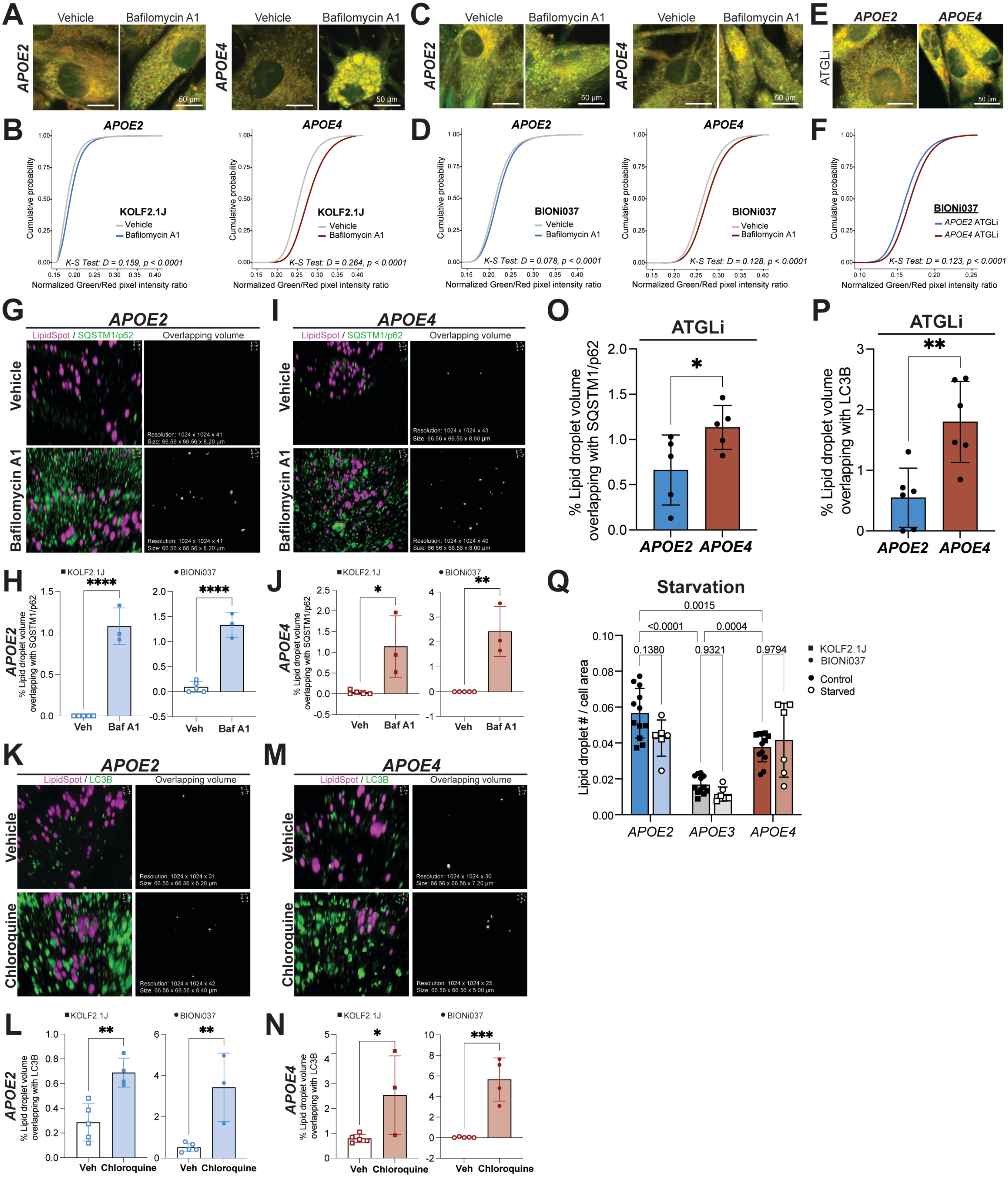

Figure S8. Characterization of lipophagy in *APOE2* and *APOE4* astrocytes
A–D. LipiDye™-M fluorescence images and cumulative green/red pixel intensity distributions from *APOE2* and *APOE4* iPSC-derived astrocytes treated with bafilomycin A1 (10 nM) or vehicle for 24 h in KOLF2.1J and BIONi037A lines. Scale bars, 50 µm. At least 30 cells were quantified per treatment; $p < 0.0001$ by Kolmogorov–Smirnov test. **E–F.** LipiDye™-M fluorescence images and cumulative green/red pixel intensity distribution from BIONi037A *APOE2* and *APOE4* astrocytes treated with ATGLi (10 µM) for 96 h. Scale bars, 50 µm. At least 180 cells were quantified per condition; $p < 0.0001$ by Kolmogorov–Smirnov test. **G–J.** SQSTM1/p62 immunofluorescence in green contacting LipidSpot-positive lipid droplets in magenta in *APOE2* or *APOE4* astrocytes after bafilomycin A1 or vehicle treatment for 24 h. Overlap volume is white. Representative KOLF2.1J 3D projections are shown; scale, 67 × 67 × 8–8.60 µm^3. Quantification shows percent lipid droplet volume colocalizing with SQSTM1/p62 across KOLF2.1J and BIONi037A lines. Data represent approximately 100–200 lipid droplets per condition and $n = 3$–5 frames per genotype; mean ± SD. Significance was tested by unpaired t-test. **K–N.** LC3B immunofluorescence in green contacting LipidSpot-positive lipid droplets in magenta in *APOE2* or *APOE4* astrocytes after chloroquine (10 µM) or vehicle treatment for 24 h. Overlap volume is white. Representative KOLF2.1J 3D projections are shown; scale, 67 × 67 × 5–8.40 µm^3. Quantification shows percent lipid droplet volume colocalizing with LC3B across KOLF2.1J and BIONi037A lines. Data represent approximately 20–500 lipid droplets per condition and $n = 3$–5 frames per genotype; mean ± SD. Significance was tested by unpaired t-test. **O–P.** Percent lipid droplet volume colocalizing with SQSTM1/p62 or LC3B after ATGLi for 96 h in BIONi037A astrocytes. Data represent approximately 350–360 lipid droplets per condition and $n = 5$ frames per genotype; mean ± SD. Significance was tested by unpaired t-test. **Q.** Lipid droplets per cell area across two isogenic astrocyte sets under control conditions or after 5 days starvation. Each point represents an independent treatment; mean ± SD. P-values were determined by two-way ANOVA with Tukey's test.
