# Supplementary material for: APOE genotypes differentially remodel the astrocytic lipid droplet proteome to shape lipid droplet dynamics
Source: Nat Commun. 2026 Aug 19;17:9900. doi: 10.1038/s41467-026-76565-6 (PMC13582944; doi:10.1038/s41467-026-76565-6)
Supplement: Supplementary file 2 [file 41467_2026_76565_MOESM2_ESM.pdf]

nature portfolio

Peer Review File

APOE genotypes differentially remodel the astrocytic lipid droplet proteome to shape lipid droplet dynamics

Corresponding Author: Dr Priyanka Narayan

This file contains all reviewer reports in order by version, followed by all author rebuttals in order by version.

Version 0:

Reviewer comments:

Reviewer #1

(Remarks to the Author)
This work by Cuní-López and colleagues is an excellent resource providing useful information regarding the astrocyte lipid droplet (LD) proteome and lipidome. While the composition of LDs in peripheral cell types such as hepatocytes has been well characterized, the composition of LDs in neural cell types has been understudied. With the emerging importance of LDs in neurodegenerative disease, this is a timely contribution. The authors provide extremely high-quality proteomics and lipidomics data sets of LDs isolated from human iPSC-derived astrocytes (iAstros). This work expands on previous reports of iPSC-derived glia cell lipidomes by focusing on LD isolation and characterization rather than using whole cell lysate. Interesting differences were observed at the LD vs. whole-cell level, highlighting the need for this type of analysis. Several of the candidate LD proteins identified by mass spec were validated by other methods, including a novel LD protein, RUFY2. Furthermore, the authors tested how APOE genotype remodels the LD proteome/lipidome to influence cellular function. The authors identified differences in the abundance of LD-associated proteins and LD lipids between APOE genotypes that could contribute to Alzheimer's Disease by influencing immune signaling, metabolic signaling, and autophagy. The data sets are presented in a clear and rigorous manner and are likely to be very useful to the field. The proteomics data implied a difference in the recruitment of autophagy-related proteins to LDs in the APOE4 genotype. This is an intriguing observation. In Figure 5, the authors present data they claim shows that autophagy of LDs (lipophagy) is impaired in APOE4 astrocytes. While the data indicate that LD turnover is reduced in APOE4 cells, the experiments do not actually distinguish whether the difference is in lipophagy or turnover by cytoplasmic lipases. Therefore, we suggest the authors either tone down these claims or expand their experiments in Figure 5 to further clarify whether lipophagy is indeed the mechanism responsible for impaired lipid droplet turnover in APOE4-expressing cells. If this major and several minor concerns can be addressed, then we recommend publication in Nature Communications.

Major concerns:

Figure 5D-E: Cells utilize two distinct mechanisms to turn over LDs: lipophagy, which is a LD-specific form of autophagy, and lipolysis, which involves recruitment of cytosolic lipases to the LDs. The fluorescent fatty acid pulse-chase data provided does not differentiate between these two mechanisms. In the original use of this assay in mouse embryonic fibroblasts, the authors showed that in that cell type the majority of LD-to-mitochondria fatty acid trafficking under HBSS starvation was due to ATGL-dependent lipolysis, and not lipophagy (PMID: 25752962). We suggest that the authors repeat the chase experiments in Figure 5D,E while including pharmacological inhibitors of lipolysis (ATGListatin) vs. lipophagy (global autophagy inhibitor, such as 3-methyladenine, bafilomycin A1, or chloroquine) to discriminate if the impaired lipid turnover is due to lipolysis or lipophagy.

Figure 5F-I: The authors demonstrate that APOE4-expressing astrocytes recruit more SQSTM1/p62 and LC3B to LDs and use % LD volume overlap as a metric. However, it is not clear how increased recruitment of an autophagy adaptor relates to reduced lipophagy. The authors hypothesize that the increased accumulation of SQSTM1/p62 on LDs is due to decreased autophagic flux, but this was not directly tested. The authors should perform an assay that actually measures autophagic flux of LDs or an LD protein. One possibility is to perform a microscopy-based lipophagic flux assay similar to that in Figure 4 of Chung et al. 2023 (PMID 37443287). Another possibility is to measure the degradation of a LD protein +/- autophagy inhibitors.

Minor concerns:

Figure 2D: The protein names are too small to be legible and should be presented in a different way.

Figure 4D: I am confused about the TG annotation. Why is only one FA specified? If chain-level resolution is available, it would be helpful to annotate all the chains.

Discussion: Can the authors speculate about the mechanism by which APOE4 affects the LD proteome/lipidome? APOE has been shown to traffic to LDs in immortalized mouse astrocytes. It was also detected in the buoyant fraction in this data set. Although it wasn't enriched enough to be classified as an LD protein (likely due to its dual localization to the secretory pathway), it could still play a role at the LD e.g., recruiting interacting partners that then affect the LD lipid composition.

Reviewer #2

(Remarks to the Author)
I co-reviewed this manuscript with one of the reviewers who provided the listed reports. This is part of the Nature Communications initiative to facilitate training in peer review and to provide appropriate recognition for Early Career Researchers who co-review manuscripts.

Reviewer #3

(Remarks to the Author)
This manuscript by Narayan and colleagues investigate lipid droplets from iPSC-derived astrocytes and the role of the main ApoE isoforms in modulating this lipid droplet biology. The authors profiled lipid droplet-associated proteins and lipids using proteomics and lipidomics, respectively. They found that each ApoE isoform is associated with distinct molecular signatures. One of the pathways they focus on is lipophagy, based on proteomics data showing is is enriched in the ApoE4 genotype. Cuni-Lopez et al showed that lipid droplets from ApoE2 astrocytes were efficiently degraded, while those in ApoE4 resisted turnover. These findings suggest that impaired clearance, rather than accumulation, underlies APOE4-related lipid phenotypes.

Overall, this is potentially an interesting study that may clarify why glial cell lipid droplets are linked to Alzheimer's disease (AD). The author show clear ApoE isoform-specific proteomes and lipidomes and potential differences in how lipids are cleared, with ApoE4-associated lipid droplets potentially resisting lipophagy-mediated lipid clearance. However, the functional/mechanistic studies are kept at a very high level, significantly limiting the impact of the work. The authors should address the following key points to maximize the impact of the manuscript.

Major comments:

A. Treating cells with oleic acid typically causes fundamental differences in lipid composition, particularly in glycerolipids, phospholipids and sterol esters even at the level of total homogenates. In fact, oleic acid (OA) should be enriched in many of these lipids (even outside lipid droplets). Rather than showing a non-informative heat map in S1E, the authors should actually show quantifications of relevant lipid species, including OA-containing lipid species. The same applies to panel 1F.

B. Some basic characterization of ApoE protein in the 3 genotypes is missing. Minimally, the authors should report cellular and secreted levels of ApoE proteins under baseline conditions and with OA treatment; additionally, because it pertains to mechanism, the authors should assess subcellular distribution of ApoE isoforms with lipid droplets, based on the work of co-author Cohen et al (PMID 38334983). This may become particularly relevant when assessing the effect of ApoE4 on lipophagy (see also below).

C. The outcome of Figure 2 and 3 is interesting in that it reveals a lipid droplet proteome in iPSC-derived astrocytes and novel proteins (and as such, an interesting resource) as well as ApoE isoform-specific proteomes. However, the impact of this part of the study is rather limited, because it is not mechanistically related to ApoE isoform biology. While analyses of lipid droplet turnover in the different cell lines suggest lipophagy defects in the ApoE4 line in Figure 5, there is no mechanism directly explaining this potential phenotype. For instance, does ApoE4 recruit autophagy adaptors to lipid droplets but prevents them from triggering lipophagy? Additionally, the functional consequences of impaired lipophagy in astrocytes are not described.

D. Generally, more work is required to demonstrate lipophagy occurs in iPSC derived astrocytes. For instance, is there direct evidence lipids are delivered to lysosomes via lipophagy? Can this be visualized with lysosomal blockers? The canonical pathway for TG hydrolysis is still very much via ATGL (neutral lipase), so lipophagy may still be a minor pathway, at least in the absence of starvation.

E. Regarding the lipidome, there are significant discrepancies observed between this study and other studies, including a recent one from van der Kant et al (also a co-author of this study) (PMID: 39005258). What could be the reasons behind these discrepancies?

F. It would be really helpful to compare the ApoE isoforms with an ApoE KO to understand how the complete ApoE loss of function model looks like in relation to the ApoE isoforms.

Minor comments:

G. LN 60: when referring to the three main APOE isoforms, it's more common to use terms like "coding variants" than actual "mutations".

H. LN47-54, Introduction: When reviewing the lipid droplet phenotypes in relevant brain cell types, the authors should probably break down the literature evidence into microglia vs astrocytes, given the vastly different roles of these two cell types in disease, and AD in particular. Additionally, being specific about mouse vs human (eg iPSC) data is also recommended, given the species specific differences.

I. LN 56-57: It would be helpful to actually mention some of the AD genes involved in lipid metabolism besides APOE

J. LN 62-64: it is also important to mention that reactive microglia express high levels of APOE; here again, it is recommended to mention whether the evidence comes from mouse vs human datasets.

K. LN64: There is also evidence APOE4/E4 human iPSC-derived microglia build up lipid droplets in response to beta-amyloid amyloid treatment (see eg PMID)

L. LN73: Generally journal policies discourage use of expressions such as "for the first time"

M. LN89: Are there just differences in lipid droplet "numbers" or sizes or cellular distributions are also affected?

N. LN 113-114: There are always some levels of organelle contaminants in subcellular fractionation experiments so the words "did not contain other contaminating organelles" should be toned down.

O. LN 121-132: In Figure S2A, the authors make the point that the lipid droplet proteome is largely overlapping between OA-treated cells and baseline. In a way, this is not surprising if equivalent protein amounts were used for proteomics. The key point is that lipid droplets may look similar, but obviously the OA treatment produces more lipid droplets. This point should be made clearer in the manuscript.

Reviewer #4

(Remarks to the Author)
This study aims to elucidate the fundamental principles behind the protective effect of APOE2 and the risk-associated effect of APOE4 in Alzheimer's disease (AD) by performing lipid droplet-specific proteomics and lipidomics on astrocytes derived from hiPSCs carrying these APOE isoforms. The research has a significant purpose, and its results will serve as an important resource. However, this study has shortcomings in the fundamental experimental design.

Major Points

The researchers stimulated lipid droplet formation by treating cells with oleic acid. They claim that "the amount and identity of buoyant fraction proteins detected was largely similar between oleic acid- and vehicle-treated conditions" and subsequently based their analyses on the oleic acid-treated group. (To strengthen the claim that the overall lipid composition is largely similar, the authors should present a quantitative comparison of lipid class proportions between the vehicle- and oleic acid-treated groups, including statistical analysis to show that any differences are not significant. This also applies to the data presented in Figure S1E.) This approach would be appropriate if the intention were to model an environment that can be simulated by 'oleic acid' treatment. However, if the goal was to investigate differences under basal conditions, wouldn't it be more appropriate to use the results from the vehicle-treated group, especially if there is little difference between the two conditions? Alternatively, the differences between the two conditions could have been used to compare basal versus specific conditions. Furthermore, it is questionable whether BSA treatment can be considered a 'basal condition,' as BSA itself can induce changes in the lipid droplet profile. For a study in the context of Alzheimer's disease, wouldn't it be more informative to perform these analyses under pathological or stress conditions, such as media (w/o FBS) +/- Aβ, or co-culture with AD neurons or other cell types, as the authors mention in the discussion?

The number of lipid droplets in APOE2 astrocytes is higher than in APOE4 under the vehicle conditions, but lower in oleic acid conditions. How do the authors interpret these data? This also suggests that the lipid droplet profiles under oleic acid and vehicle treatments may differ.

The omics analyses were performed by combining two isogenic lines. However, crucial information is missing, making the data very confusing. For each column in the heatmaps and plots (e.g., Figure 1F, Figure 4A, Figure S1E, Figure 2B, Figure S3), the origin of the sample (which cell line) should be provided. The number of samples per isoform varies, and some isoforms are absent in certain representations. This is particularly concerning given that the cell lines have different sexes; it is necessary to check for line-specific differences, even if there is only one line per sex. Was the data from the two other lines normalized separately before comparison? Was each column generated by pooling multiple cultures or wells?

What is the criterion for using the KOLF2.1J line in some experiments and the BIONi037 line in others? Are the results from both lines shown alternately? A direct comparison of the results between the lines may be needed, as they are from different sexes, also, given that the two cell lines seem to be maintained and differentiated using different methods (e.g., Matrigel vs.

Geltrex for culture, and distinct astrocyte differentiation protocols).

Minor Points

The manuscript contains several strong claims that lack sufficient experimental support (e.g., Line 218, Line 262). For example, the interpretation in Line 333 regarding the role of immune proteins is also oversimplified, as inflammation can be beneficial or detrimental. Line 344: The observed lipophagy impairment could result from a general defect in the autophagy machinery in APOE4 astrocytes, which has been reported in multiple studies. The authors claim to provide a "mechanistic basis" (Line 306), but no precise mechanisms are elucidated.

Can these observations be confirmed in human AD brains? At a minimum, demonstrating the distinct expression of lipid-associated proteins between APOE isoforms would be valuable.

In the abstract, the phrase "impaired lipid droplet clearance, rather than accumulation" is confusing. Isn't accumulation the net result of synthesis and degradation? This phrasing seems paradoxical. Furthermore, as mentioned earlier, lipid droplet accumulation in APOE4 astrocytes is more pronounced with oleic acid treatment than other isoforms. Do the authors attribute this enhanced increase solely to impaired clearance?

Figure 1E: Is the GAPDH signal observed in the ER/mitochondria fraction comparable to the cytosol fraction? In addition, Coomassie blue staining is needed to confirm loading consistency.

Line 163: The study identifies 403 lipid droplet-specific proteins. (The criteria for defining astrocytic lipid droplet-associated proteins are unclear. Why were the top 2.5% of proteins selected?) Shouldn't the subsequent comparative analysis (Line 194) be restricted to this set of proteins?

For Figures 2H, 3D, 3G, and S2C, quantification of the co-localization ratio (i.e., the proportion of the protein co-localizing with lipid droplets) should be provided to be more informative.

Figure 5B, S5A: Are these analyses based on only 3-5 cell images per condition? If so, the sample size is too small to draw robust conclusions.

The images in Figure 3G and Figure S5A do not appear to support the finding from Figure 1 that APOE4 astrocytes have more lipid droplets than other isoforms.

As mentioned, it is well established that APOE4 astrocytes accumulate more lipids compared to APOE3 astrocytes. To support this, statistical information under vehicle conditions should be provided in Figure 1C. However, in Figure S1D, this trend is not observed in the BIONi037 line.

Line 300: The authors state that APOE2 shows a trend toward reduced lipid droplet accumulation under starvation conditions. However, it is not statistically significant, and the description should be adjusted to avoid overinterpretation.

Reviewer #5

(Remarks to the Author)
I co-reviewed this manuscript with one of the reviewers who provided the listed reports. This is part of the Nature Communications initiative to facilitate training in peer review and to provide appropriate recognition for Early Career Researchers who co-review manuscripts.

Reviewer #6

(Remarks to the Author)
Review of the manuscript entitled APOE genotypes differentially remodel the astrocytic lipid droplet-associated proteome to shape lipid droplet dynamics.
The manuscript is well written, and the results could be of interest to the broader readership of Nature Communicaiton. However, based on the proteomics data presented here I have several concerns that has to be addressed to consider the manuscript for publication.

Major concerns:
The authors write that they are maximizing the number of lipid droplets in the astrocytes by stimulation with Oleic acid. Could this treatment influence the findings later?
The authors claim that the treatment with Oleic acid is not “substantially altering the overall lipid composition of the cell”. The figure S1E contains quite a lot of changes, so what is this statement based on? Also, why does the Figure not show APOE3 without the treatment? The figure should be reorganized so that the Vehicle and the Oleic acid treatment for each cell line is next to each other to allow visual evaluation of the changes associated with the treatment.
The authors do not include how many replicates they are using for the analysis. From the supplementary tables it looks like they are using very few replicates, and maybe even only from technical replicates. This must be explained better. If it is only technical replicates, The study is not publishable as proteomics and especially DIA proteomics require at least 3 biological

replicates. This reviewer has downloaded some of the data raw files and search them in Spectronaut. Here it is evident that some of the replicates are very far from each other in the number of proteins/peptides they identify. For example, the result from APOE3 droplet 1 give 2255 proteins whereas APOE droplet 2 give 4845 proteins using the same settings, indicating a very poor reproducibility in the enrichment and whole analysis. Therefore at least 3 biological replicates MUST be used for this analysis, and more replicates if the raw data files give so large difference in number of proteins.
The authors used DIA on an Eclipse Mass spectrometer, that can run 14 Hz at 30.000 Resolution. The loop control which I believe is the time between each MS is set to 3 seconds. The authors are not including important details on how the data are processed in Spectronaut. Was the quantitation performed on the MS1 or the MS2 level? If the last strategy was used, then how many peaks over the elution profile were obtained? Enough to make a solid quantification? What are the CVs? The authors write that they have 75 windows per scan cycle with 8 Da windows and at a scan rate of 14 Hz the completion of the 75 windows will take more than 5 seconds. This will not give enough points over the elution profile to use MS2 based quantitation unless very bad chromatography. Such details need to be present in the manuscript and the setting for the DIA must be justified.
The authors write that they are using p-values of <0.1? Why such a high p-value? Normally in proteomics 0.05 is used and there needs to be a very good explanation for using 0.1 such as tissue samples or samples from individuals. Here the authors are using iPSC lines which is not enough to justify p<0.1.
Out of the 18 Oleic acid specific proteins there are 3 transcription factors. Can the authors explain this? Why would transcription factors be present in lipid droplets? Has this been observed before? Also, the Supplementary Table 1 I assume is excel sheet 1. BF_OAvsVeh? This should be labeled better.
There needs to be a dedicated Table with all identified proteins from the Spectronaut DIA analysis, including how many peptides that are identified per protein and abundances in all individual replicates for all the cell lines and lipid droplet enrichments and standard deviations including p-values and adjusted p-values must be included. Proteins that are only identified and quantified using 1 single peptide should be eliminated from the analysis. From the Spectronout searches of APOE2 droplet 1 and 2 and APOE3 droplet 1 and 2 there are significant amounts of the proteins that are identified with only one peptide (+12% or more). Having only one peptide in DIA is not good enough for a solid identification and normally in proteomics one would use 2 or more peptides for quantitation. At least 2-3 peptides in DIA should be present to make a valid quantitation. Furthermore, the number of missing values between the samples to compare is very high which is indicative of either very different samples or poor analysis in LC-MSMS. Imputation is not a valid method with very few replicates.
In Figure 2A the authors describe their workflow to identify “the human iPSC-derived astrocyte lipid droplet-associated proteome”. But from the lists in the supplementary tables, it is a bit difficult to see how the authors came to these numbers? Please make a thorough explanation here.
In line 125-131 the authors write that the 18 proteins specific in the Oleic acid treated fraction is probably because there are more lipid droplets in these cells. However, in figure 1C there are very large differences between the treated and non-treaded samples with respect to number of lipid droplets, and it is a bit strange that such a large difference only results in a difference of 18 proteins? Can the authors comment on this? How many of the 18 proteins were identified by only 1-2 peptides in the DIA analysis?
Line 172-175: The authors say that RNA biology has not been linked to lipid droplets before? This is not entirely true, as the spliceosome has previously been linked to lipid droplets (e.g. PMID: 24333419).
Line 196-205: The data used for generating the lists of protein in lipid droplets from APOE2-4 are highly questionable as it seems that there have only been analyzed technical replicates. This is not enough for statistical analysis and is normally not valid within the field. At least 3 biological replicates must be included in the study, and a proper statistical test must be applied. Single peptide proteins (one-hit wonders) are also not used in the field, especially not for quantitative DIA analyses.

Version 1:

Reviewer comments:

Reviewer #1

(Remarks to the Author)
The authors have thoughtfully addressed our concerns and significantly strengthened the study. They are to be commended on a comprehensive and rigorous body of work. We recommend publication in Nature Communications.

Reviewer #2

(Remarks to the Author)
I co-reviewed this manuscript with one of the reviewers who provided the listed reports. This is part of the Nature Communications initiative to facilitate training in peer review and to provide appropriate recognition for Early Career Researchers who co-review manuscripts.

Reviewer #3

(Remarks to the Author)
The authors have addressed all my questions and concerns

Reviewer #4

(Remarks to the Author)
I would like to thank the authors for their significant efforts to address my previous concerns. While the manuscript has

improved, there are still several critical issues that need to be resolved:

- The authors state in the main text that, ‘With vehicle control treatment, all APOE genotypes of astrocytes still accumulated more lipid droplets than APOE3 astrocytes.’ However, this interpretation is heavily biased. In the BIONi037 line, this trend is completely absent (as shown in Figure S1F). I previously pointed out that differences in media and differentiation methods between the cell lines could account for these variations, but the authors largely dismissed this as parental line variation. While it is acceptable to focus on common phenotypes across models, the authors must carefully avoid making biased or overly general statements (such as the one regarding baseline APOE3 lipid droplet accumulation) that are explicitly contradicted by their own data.

- The authors acknowledged in the rebuttal that Amyloid-beta treatment did not alter lipid droplet accumulation, whereas OA treatment did. Even though the authors claim there are no significant differences between the vehicle and OA-treated groups, quantitative differences are evident, and the authors’ own data demonstrate that OA treatment alters APOE levels. This raises a fundamental question: what physiological or pathological condition does this OA treatment mimic? Even if OA treatment is primarily a strategy of ‘experimental convenience’ to increase lipid droplet yield, the authors must rigorously discuss what real-world environment this model represents. Furthermore, the reliance on this specific, artificial context must be explicitly stated in both the title and the main text.

- During the revision process, the authors generated and added two new proteomics samples exclusively to the APOE3 group and re-analyzed the entire dataset. This reanalysis led to different results and interpretations than those in the original manuscript. Batch effects are an inevitable reality when repeating experiments at different times. Therefore, any additional runs must include samples from all experimental groups simultaneously. Adding new samples to only one specific group and merging them with old data is methodologically unacceptable. Given that this post-hoc addition altered the overall conclusions of the study, these results must be viewed with extreme caution, and this fundamental flaw in the experimental design must be addressed.

Reviewer #5

(Remarks to the Author)
I co-reviewed this manuscript with one of the reviewers who provided the listed reports. This is part of the Nature Communications initiative to facilitate training in peer review and to provide appropriate recognition for Early Career Researchers who co-review manuscripts.

Reviewer #6

(Remarks to the Author)
The authors have answered all my concerns and performed new experiments to clarify and make their proteomics data stronger.

Open Access This Peer Review File is licensed under a Creative Commons Attribution 4.0 International License, which permits use, sharing, adaptation, distribution and reproduction in any medium or format, as long as you give appropriate credit to the original author(s) and the source, provide a link to the Creative Commons license, and indicate if changes were

made.
In cases where reviewers are anonymous, credit should be given to 'Anonymous Referee' and the source.
The images or other third party material in this Peer Review File are included in the article's Creative Commons license, unless indicated otherwise in a credit line to the material. If material is not included in the article's Creative Commons license and your intended use is not permitted by statutory regulation or exceeds the permitted use, you will need to obtain permission directly from the copyright holder.
To view a copy of this license, visit https://creativecommons.org/licenses/by/4.0/

We thank the reviewers for their constructive feedback on our manuscript. We have made a concerted effort to address the points raised by the reviewers. We believe that these revisions substantiate our data more thoroughly and greatly improve the paper. A point-by-point response to the reviewers' comments is below. We have also demarcated added or changed text in the manuscript file in red.

REVIEWER COMMENTS

Reviewer #1 (Remarks to the Author):

This work by Cuní-López and colleagues is an excellent resource providing useful information regarding the astrocyte lipid droplet (LD) proteome and lipidome. While the composition of LDs in peripheral cell types such as hepatocytes has been well characterized, the composition of LDs in neural cell types has been understudied. With the emerging importance of LDs in neurodegenerative disease, this is a timely contribution. The authors provide extremely high-quality proteomics and lipidomics data sets of LDs isolated from human iPSC-derived astrocytes (iAstros). This work expands on previous reports of iPSC-derived glia cell lipidomes by focusing on LD isolation and characterization rather than using whole cell lysate. Interesting differences were observed at the LD vs. whole-cell level, highlighting the need for this type of analysis. Several of the candidate LD proteins identified by mass spec were validated by other methods, including a novel LD protein, RUFY2. Furthermore, the authors tested how APOE genotype remodels the LD proteome/lipidome to influence cellular function. The authors identified differences in the abundance of LD-associated proteins and LD lipids between APOE genotypes that could contribute to Alzheimer's Disease by influencing immune signaling, metabolic signaling, and autophagy. The data sets are presented in a clear and rigorous manner and are likely to be very useful to the field. The proteomics data implied a difference in the recruitment of autophagy-related proteins to LDs in the APOE4 genotype. This is an intriguing observation. In Figure 5, the authors present data they claim shows that autophagy of LDs (lipophagy) is impaired in APOE4 astrocytes. While the data indicate that LD turnover is reduced in APOE4 cells, the experiments do not actually distinguish whether the difference is in lipophagy or turnover by cytoplasmic lipases. Therefore, we suggest the authors either tone down these claims or expand their experiments in Figure 5 to further clarify whether lipophagy is indeed the mechanism responsible for impaired lipid droplet turnover in APOE4-expressing cells. If this major and several minor concerns can be addressed, then we recommend publication in Nature Communications.

Major concerns:

Figure 5D-E: Cells utilize two distinct mechanisms to turn over LDs: lipophagy, which is a LD-specific form of autophagy, and lipolysis, which involves recruitment of cytosolic lipases to the LDs. The fluorescent fatty acid pulse-chase data provided does not differentiate between these two mechanisms. In the original use of this assay in mouse

embryonic fibroblasts, the authors showed that in that cell type the majority of LD-to-mitochondria fatty acid trafficking under HBSS starvation was due to ATGL-dependent lipolysis, and not lipophagy (PMID: 25752962). We suggest that the authors repeat the chase experiments in Figure 5D,E while including pharmacological inhibitors of lipolysis (ATGListatin) vs. lipophagy (global autophagy inhibitor, such as 3-methyladenine, bafilomycin A1, or chloroquine) to discriminate if the impaired lipid turnover is due to lipolysis or lipophagy.

We thank the reviewer for this experimental suggestion. As noted by the reviewer, our initial pulse-chase experiments showed slower lipid droplet turnover in *APOE4* astrocytes compared to *APOE2* within 24 h (**Figure 5D,E** and **S7C**). However, this assay did not distinguish between the two main mechanisms of lipid droplet degradation—lipolysis and lipophagy. To specifically measure degradation mediated by lipophagy, we inhibited lipolysis with the chemical inhibitor atglistatin (ATGLi), which inhibits the rate-limiting lipase ATGL. We confirmed that ATGLi treatment increased lipid droplet levels in *APOE2* and *APOE4* astrocytes (**Figure S7D,E**), suggesting efficient inhibition of lipid droplet breakdown in astrocytes. We then repeated a pulse-chase assay using BODIPY-C_{12} in the presence of ATGLi. Over the course of the experiment, we observed degradation of lipid droplets in both *APOE4* and *APOE2* astrocytes (**Figure S7F,G**). However, at the end of the timecourse the quantity of lipid droplets remaining (which had not been degraded via lipophagy) was higher in *APOE4* than *APOE2* (**Figure 5F** and **S7H**), indicating that *APOE2* lipid droplets are more easily turned over by lipophagy than *APOE4* lipid droplets. We have incorporated these findings in the manuscript with a revised version of **Figure 5** and two new supplementary figures (**S7** and **S8**), and as additional text (lines 368-413).

Figure 5F-I: The authors demonstrate that APOE4-expressing astrocytes recruit more SQSTM1/p62 and LC3B to LDs and use % LD volume overlap as a metric. However, it is not clear how increased recruitment of an autophagy adaptor relates to reduced lipophagy. The authors hypothesize that the increased accumulation of SQSTM1/p62 on LDs is due to decreased autophagic flux, but this was not directly tested. The authors should perform an assay that actually measures autophagic flux of LDs or an LD protein. One possibility is to perform a microscopy-based lipophagic flux assay similar to that in Figure 4 of Chung et al. 2023 (PMID 37443287). Another possibility is to measure the degradation of a LD protein +/- autophagy inhibitors.

We appreciate this suggestion. We were able to trial the plasmid-based Keima-LiveDrop system reported by Chung et al 2023. Even though we successfully transfected the astrocytes with this construct, we did not see the expected shift in Keima fluorescence when using positive controls. This was likely due to the quick lysosomal cleavage of the Keima reporter (we confirmed through email correspondence that Dr. Chung saw this same effect in her 2023 study, making the reporter difficult to use for experiments lasting more than a few hours). Therefore, we chose another reporter system to address the question of our study.

We identified LipiDye™-M, a solvatochromic dye-labeled C_{12} fatty acid that has been previously used to monitor lipid metabolism under lipolysis and autophagy inhibition conditions [1,2]. Chloroquine and bafilomycin A1 treatment of the astrocytes (positive controls) confirmed that stalling autophagy increases the green-to-red pixel ratio of LipiDye™-M compared to vehicle control (**Figure S8A-D**). We then inhibited lipolysis (ATGLi treatment) in our *APOE2* and *APOE4* astrocytes and observed an increase in green-to-red fluorescent ratio in *APOE4* cells compared to *APOE2* (**Figure 5I,J** and **S8E,F**). This suggests that the LipiDye™-M signal in *APOE4* when lipolysis is inhibited resembles stalled lipophagy.

We have included additional immunofluorescence analysis of autophagy adaptors (SQSTM1/p62 and LC3B) co-localized with lipid droplets after chemically inhibiting lipophagy and lipolysis. We observed a higher overlap between adaptor proteins and lipid droplets in the context of reduced lipophagy flux resulting from bafilomycin A (**Figure S8G-J**) and chloroquine treatments (**Figure S8K-N**). Similarly, *APOE4* lipid droplets were more co-localized with SQSTM1/p62 and LC3B than *APOE2* under lipolysis inhibition with ATGLi (**Figure 5K-N** and **S8O,P**), suggesting impaired lipophagy in *APOE4*.

Together with our pulse-chase assay, these two additional experimental approaches (lines 386-413) lead us to conclude that lipophagy is reduced in *APOE4* astrocytes compared to *APOE2* and provide a functional validation of our proteomics observations.

Minor concerns:

Figure 2D: The protein names are too small to be legible and should be presented in a different way.

Thank you for noting this. We have enlarged **Figure 2D**. These protein names are also included in **Supplementary Table 7**.

Figure 4D: I am confused about the TG annotation. Why is only one FA specified? If chain-level resolution is available, it would be helpful to annotate all the chains.

Thank you for this comment. For the Lipidyzer platform used in this study, triacylglycerols are not fully resolved to the sn-position level, and the complete composition of all three fatty acyl chains cannot be assigned unambiguously. Instead, reported triacylglycerol species are primarily annotated by the summed composition of the three acyl chains, expressed as the total number of carbon atoms and double bonds. Importantly, the platform can identify one constituent fatty acid chain based on characteristic fragmentation, while the remaining two chains are reported as part of the summed composition. This partial fatty acyl information is indicated by specifying the identified acyl

chain. For example, a triacylglycerol containing 54 total carbons and one double bond, in which an oleic acid chain is identified, would be annotated as TG 54:1-FA18:1.

Discussion: Can the authors speculate about the mechanism by which APOE4 affects the LD proteome/lipidome? APOE has been shown to traffic to LDs in immortalized mouse astrocytes. It was also detected in the buoyant fraction in this data set. Although it wasn't enriched enough to be classified as an LD protein (likely due to its dual localization to the secretory pathway), it could still play a role at the LD e.g., recruiting interacting partners that then affect the LD lipid composition.

The reviewers are correct. We did detect APOE in our oleic acid-treated buoyant fractions of all *APOE* genotypes by mass spectrometry. Across two isogenic sets there was no significant difference in the abundance of APOE on lipid droplets (data below and added to **Figure S2C**).

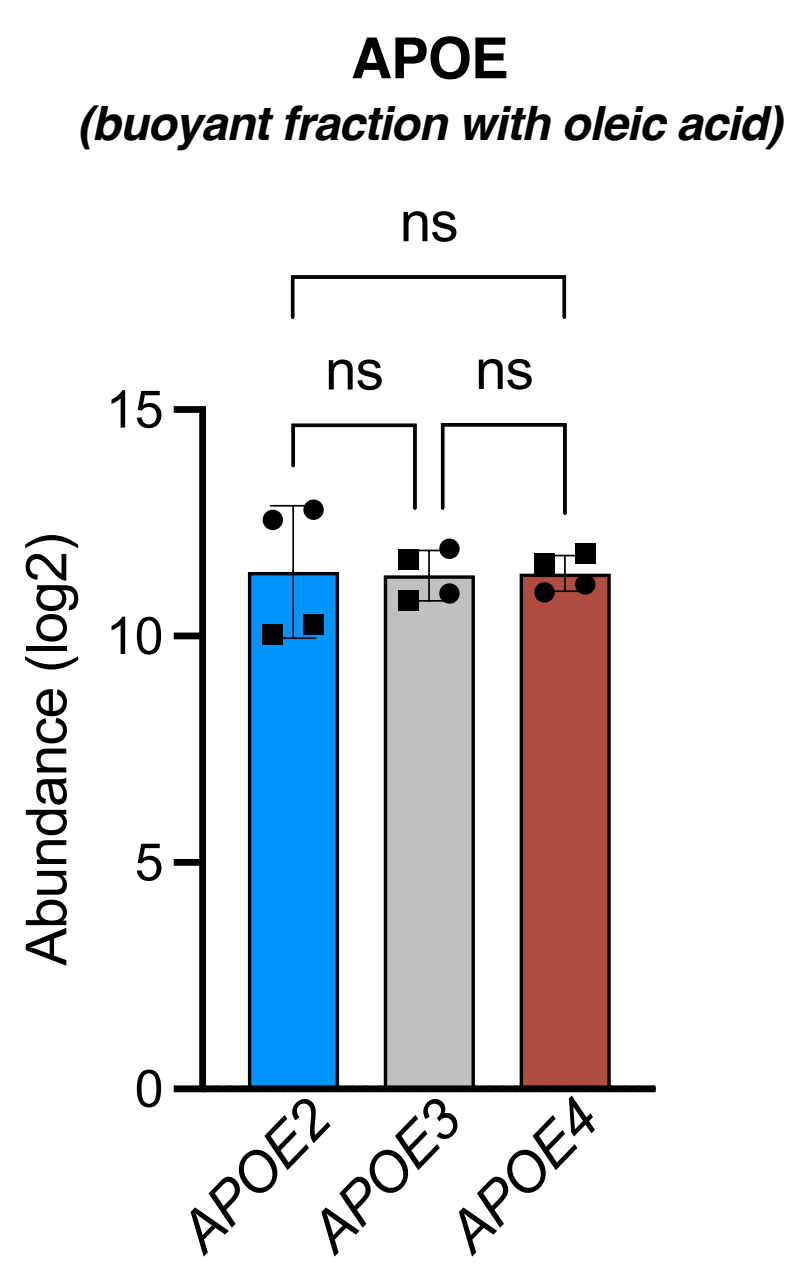

Lipid droplets bud from the ER membrane and therefore can reflect the contents and perturbations in ER biology. Since the APOE isoforms are present in the secretory pathway, we hypothesize that the APOE isoforms can modulate the lipid droplet protein and lipid content via interactions with proteins and lipids in the ER. We suspect that *APOE* genotype modulates protein and lipid interactions in the ER, which then results in different genotype-specific LD proteomes. We have included a discussion of APOE and its role in this process in the manuscript **Discussion** section (lines 490-497).

Reviewer #2 (Remarks to the Author):

I co-reviewed this manuscript with one of the reviewers who provided the listed reports. This is part of the Nature Communications initiative to facilitate training in peer review and

to provide appropriate recognition for Early Career Researchers who co-review manuscripts.

Reviewer #3 (Remarks to the Author):

This manuscript by Narayan and colleagues investigate lipid droplets from iPSC-derived astrocytes and the role of the main ApoE isoforms in modulating this lipid droplet biology. The authors profiled lipid droplet-associated proteins and lipids using proteomics and lipidomics, respectively. They found that each ApoE isoform is associated with distinct molecular signatures. One of the pathways they focus on is lipophagy, based on proteomics data showing is is enriched in the ApoE4 genotype. Cuni-Lopez et al showed that lipid droplets from ApoE2 astrocytes were efficiently degraded, while those in ApoE4 resisted turnover. These findings suggest that impaired clearance, rather than accumulation, underlies APOE4-related lipid phenotypes.

Overall, this is potentially an interesting study that may clarify why glial cell lipid droplets are linked to Alzheimer's disease (AD). The author show clear ApoE isoform-specific proteomes and lipidomes and potential differences in how lipids are cleared, with ApoE4-associated lipid droplets potentially resisting lipophagy-mediated lipid clearance. However, the functional/mechanistic studies are kept at a very high level, significantly limiting the impact of the work. The authors should address the following key points to maximize the impact of the manuscript.

Major comments:

A. Treating cells with oleic acid typically causes fundamental differences in lipid composition, particularly in glycerolipids, phospholipids and sterol esters even at the level of total homogenates. In fact, oleic acid (OA) should be enriched in many of these lipids (even outside lipid droplets). Rather than showing a non-informative heat map in S1E, the authors should actually show quantifications of relevant lipid species, including OA-containing lipid species. The same applies to panel 1F.

Thank you for this suggestion. We replaced *Figure S1E* by **Figure S3A**, which shows bar plots of several abundant lipid classes containing oleic acid (18:1). These include triacylglycerols, cholesterol esters, diacyglycerols, free fatty acids, phosphatidylcholine and phosphatidylethanolamine from both the buoyant fraction and whole cell samples (**Figure S3A**). These analyses showed that oleic acid treatment induces the strongest changes in quantities of neutral lipids and neutral lipid precursors, which are the primary component of lipid droplets (lines 149-154). We have also included these lipid data in **Supplementary Table 21**.

B. Some basic characterization of ApoE protein in the 3 genotypes is missing. Minimally, the authors should report cellular and secreted levels of ApoE proteins under baseline conditions and with OA treatment; additionally, because it pertains to mechanism,3w the

authors should assess subcellular distribution of ApoE isoforms with lipid droplets, based on the work of co-author Cohen et al (PMID 38334983). This may become particularly relevant when assessing the effect of ApoE4 on lipophagy (see also below).

Thank you for this suggestion. We have included an additional supplementary figure with data on levels of secreted and intracellular APOE measured by ELISA and western blot, respectively, as well as immunofluorescence staining to determine the subcellular localization of APOE protein (**Figure S2** and lines 133-141). We observed *APOE* genotype differences in intracellular and secreted APOE levels that were specific to the parental iPSC line. Moreover, we noticed that oleic acid severely decreased secreted APOE compared to vehicle control (BSA). Immunofluorescence analysis showed that APOE is primarily localized to the secretory pathway rather than on lipid droplets. We have detected APOE in the buoyant fraction of oleic acid-treated iPSC-derived astrocytes by mass spectrometry, however, the abundance of APOE did not differ across genotypes (**Figure S2C**).

C. The outcome of Figure 2 and 3 is interesting in that it reveals a lipid droplet proteome in iPSC-derived astrocytes and novel proteins (and as such, an interesting resource) as well as ApoE isoform-specific proteomes. However, the impact of this part of the study is rather limited, because it is not mechanistically related to ApoE isoform biology. While analyses of lipid droplet turnover in the different cell lines suggest lipophagy defects in the ApoE4 line in Figure 5, there is no mechanism directly explaining this potential phenotype. For instance, does ApoE4 recruit autophagy adaptors to lipid droplets but prevents them from triggering lipophagy? Additionally, the functional consequences of impaired lipophagy in astrocytes are not described.

We thank the reviewer for this suggestion. Past studies have reported potential mechanisms that may link *APOE4* to impaired autophagy. One study used brain tissues from Alzheimer's disease patients to show that APOE4 binds specific DNA motifs in the promoters of several autophagy genes (including *SQSTM1/p62* and *MAP1LC3B*) and blocks their transcription[3]. Further work demonstrated that APOE4 binds these regions with more affinity than APOE2 and APOE3[4], suggesting reduced autophagy in *APOE4* compared to the other isoforms. Another potential explanation is that *APOE* isoforms modulate the lipid composition and, indirectly, the protein composition of lipid droplets. Since APOE, a lipid binding protein, is localized to the secretory pathway, it is possible that *APOE* isoforms differentially impact the lipids that are packaged into lipid droplets thereby impacting the number and identity of proteins recruited to these droplets. We have added notes on these mechanisms to the **Discussion** (lines 469-474, 490-497).

Given the key role of lipid droplets in buffering cellular stress, we speculate that the defective breakdown of these organelles could render *APOE4* astrocytes more susceptible to extrinsic stressors. Similarly, defective astrocytic non-cell-autonomous functions could impact the homeostasis of neighboring cells like neurons. Our past work on lipid droplets in microglia suggest a role for turnover in inflammatory activation[5]. A recent study

positions cholesterol esters as a regulator of antigen presentation in astrocytes[6]. We agree that exploring the functional consequences of altered lipophagy or other *APOE* genotype-specific lipid droplet phenotypes in human astrocytes is an excellent topic for future studies.

D. Generally, more work is required to demonstrate lipophagy occurs in iPSC derived astrocytes. For instance, is there direct evidence lipids are delivered to lysosomes via lipophagy? Can this be visualized with lysosomal blockers? The canonical pathway for TG hydrolysis is still very much via ATGL (neutral lipase), so lipophagy may still be a minor pathway, at least in the absence of starvation.

We thank the reviewer for these questions. To measure lipophagy directly, we have optimized a paradigm where we have successfully inhibited lipolysis with atglistatin (ATGLi), a chemical inhibitor of the lipase ATGL (**Figure S7D,E**). By repeating the pulse-chase assay with ATGLi we observed that lipid droplet breakdown occurs at a later timepoint (42 h) (**Figure S7F,G**) than without lipolysis inhibition (24 h) (**Figure 5D** and **S7C,F,G**), suggesting that lipolysis and lipophagy could happen sequentially in human astrocytes. Next, we attempted to visualize lipid droplet turnover in the context of ATGLi inhibition using the LipiDye™-M reporter. This reporter is a solvatochromic dye-labeled C_{12} fatty acid analog that has been used to track the metabolism of fatty acids in cells[1,2]. We observed that the ratiometric fluorescent signal of LipiDye™-M in *APOE4* astrocytes with ATGLi treatment mimicked that of conditions of stalled autophagy resulting from chemical treatments with the autophagy blockers bafilomycin A1 and chloroquine (**Figure 5H-J** and **S8A-F**). We have included these new data in the revised version of the manuscript (lines 368-413).

We also used immunofluorescence to analyze the co-localization of autophagy adaptors (SQSTM1/p62 and LC3B) with lipid droplets after chemically inhibiting lipophagy and lipolysis. We observed a higher overlap between adaptor proteins and lipid droplets in the context of reduced lipophagy flux resulting from bafilomycin A and chloroquine treatments (**Figure S8G-J** and **S8K-N**). Similarly, *APOE4* lipid droplets were more co-localized with SQSTM1/p62 and LC3B than *APOE2* under lipolysis inhibition with ATGLi for 96 h (**Figure 5K-N** and **S8O,P**), consistent with impaired lipophagy in *APOE4*.

E. Regarding the lipidome, there are significant discrepancies observed between this study and other studies, including a recent one from van der Kant et al (also a co-author of this study) (PMID: 39005258). What could be the reasons behind these discrepancies?

Thank you for noting this. As Dr. van der Kant and Dr. Giera are co-authors on our study, we were curious about this as well. As the Feringa et al (Neurolipid Atlas) study shows[7], media conditions impact the lipid content of cells. In our study, iPSC-derived astrocytes were grown in serum-free media for 4 days prior to treatment with oleic acid or vehicle (BSA), whereas in the Feringa et al study cells were cultured in serum-free media for only 24 h. We

repeated the whole cell lipidomics of *APOE3* and *APOE4* astrocytes in the BIONi037 background treated with our vehicle conditions, or the 24 h serum withdrawal paradigm reported by Feringa et al. This side-by-side comparison revealed that these culture media paradigms change the global cellular lipid composition of astrocytes (**Figure S3B**), and the abundance of certain lipid classes varies in an *APOE* genotype-specific manner (**Figure S3C**). We have noted these findings in a new supplementary figure (**Figure S3**), **Supplementary Table 22**, and as new text (lines 154-161) in the revised version of the manuscript.

F. It would be really helpful to compare the ApoE isoforms with an ApoE KO to understand how the complete ApoE loss of function model looks like in relation to the ApoE isoforms.

Thank you for this suggestion. We have characterized an *APOE-KO* iPSC line in the KOLF2.1J background and observed that its lipid droplet content mirrored that of both *APOE2* and *APOE4* with high numbers of lipid droplets (**Figure S1C,D**). We also observed that oleic acid stimulated lipid droplet biogenesis in the *APOE-KO* line, in agreement with the other *APOE* genotypes tested (**Figure S1G**). We have included these data in a revised version of **Figure S1** and in the text (lines 116-118 and 127-128).

Minor comments:

G. LN 60: when referring to the three main APOE isoforms, it's more common to use terms like "coding variants" than actual "mutations".

Thanks for pointing this out. We have standardized our language (lines 77-78).

H. LN47-54, Introduction: When reviewing the lipid droplet phenotypes in relevant brain cell types, the authors should probably break down the literature evidence into microglia vs astrocytes, given the vastly different roles of these two cell types in disease, and AD in particular. Additionally, being specific about mouse vs human (eg iPSC) data is also recommended, given the species specific differences.

This is a great suggestion. We have expanded our review of the literature with cell type and model specific notes in the introduction (lines 55-70).

I. LN 56-57: It would be helpful to actually mention some of the AD genes involved in lipid metabolism besides APOE

Yes, great suggestion. We have now added this information to the introduction as well (lines 74-75).

J. LN 62-64: it is also important to mention that reactive microglia express high levels of APOE; here again, it is recommended to mention whether the evidence comes from mouse

vs human datasets.

Yes, we have now included this in lines 81-83.

K. LN64: There is also evidence APOE4/E4 human iPSC-derived microglia build up lipid droplets in response to beta-amyloid amyloid treatment (see eg PMID)

Yes, we have in fact replicated this finding in our own lab's work on microglia. Interestingly, astrocytes behaved differently in our hands —amyloid-beta treatment did not appreciably change LD levels in astrocytes independent of *APOE* genotype (data below, *not included in manuscript).*

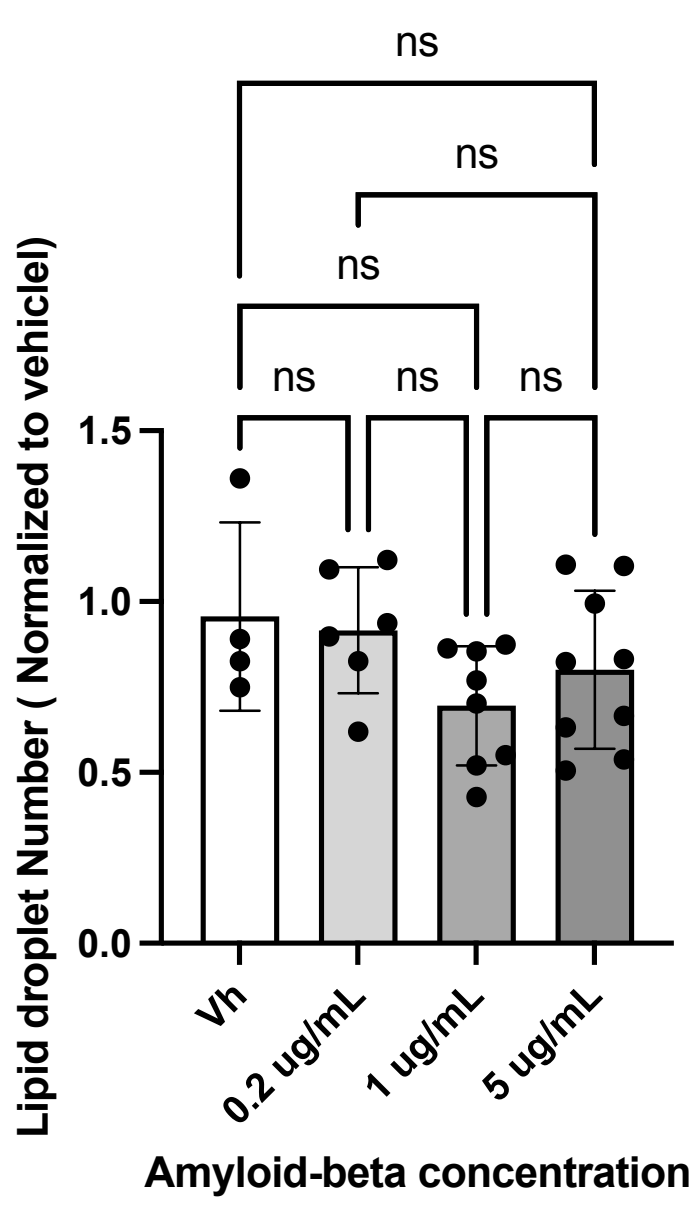

L. LN73: Generally journal policies discourage use of expressions such as "for the first time"

Thanks for noting this—we have removed this phrasing.

M. LN89: Are there just differences in lipid droplet "numbers" or sizes or cellular distributions are also affected?

Thank you for raising this point. We have analyzed our previous experiments showing increased lipid droplet numbers with oleic acid in astrocytes from the KOLF2.1J (**Figure 1B,C**) and BIONi037 (**Figure S1E,F**) backgrounds. To compare lipid droplet size across genotypes we quantified the mean area of lipid droplets. We observed strong iPSC line-specific effects on lipid droplet size (data below, *not included in manuscript*). Given this

variability per line, we could not conclude whether *APOE* genotype impact lipid droplet size beyond number.

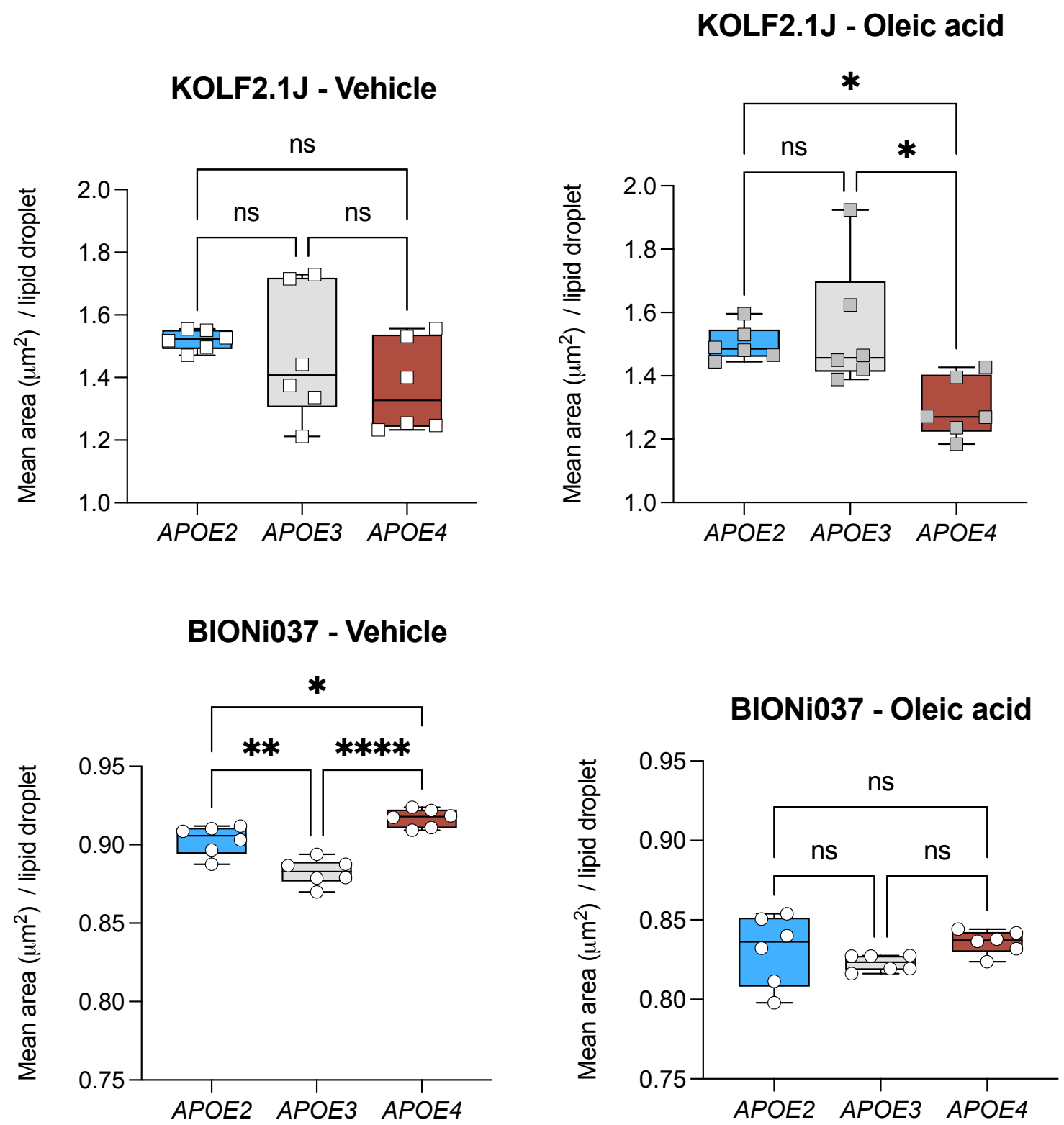

N. LN 113-114: There are always some levels of organelle contaminants in subcellular fractionation experiments so the words "did not contain other contaminating organelles" should be toned down.

Thanks for noting this—we have adjusted our language (lines 145-146).

O. LN 121-132: In Figure S2A, the authors make the point that the lipid droplet proteome is largely overlapping between OA-treated cells and baseline. In a way, this is not surprising if equivalent protein amounts were used for proteomics. The key point is that lipid droplets may look similar, but obviously the OA treatment produces more lipid droplets. This point should be made clearer in the manuscript.

Thanks for noting this. We have added a sentence to this paragraph to clarify this point (lines 169-171).

Reviewer #4 (Remarks to the Author):

This study aims to elucidate the fundamental principles behind the protective effect of APOE2 and the risk-associated effect of APOE4 in Alzheimer's disease (AD) by performing

lipid droplet-specific proteomics and lipidomics on astrocytes derived from hiPSCs carrying these APOE isoforms. The research has a significant purpose, and its results will serve as an important resource. However, this study has shortcomings in the fundamental experimental design.

Major Points

The researchers stimulated lipid droplet formation by treating cells with oleic acid. They claim that "the amount and identity of buoyant fraction proteins detected was largely similar between oleic acid- and vehicle-treated conditions" and subsequently based their analyses on the oleic acid-treated group. (To strengthen the claim that the overall lipid composition is largely similar, the authors should present a quantitative comparison of lipid class proportions between the vehicle- and oleic acid-treated groups, including statistical analysis to show that any differences are not significant. This also applies to the data presented in Figure S1E.) This approach would be appropriate if the intention were to model an environment that can be simulated by 'oleic acid' treatment. However, if the goal was to investigate differences under basal conditions, wouldn't it be more appropriate to use the results from the vehicle-treated group, especially if there is little difference between the two conditions? Alternatively, the differences between the two conditions could have been used to compare basal versus specific conditions. Furthermore, it is questionable whether BSA treatment can be considered a 'basal condition,' as BSA itself can induce changes in the lipid droplet profile. For a study in the context of Alzheimer's disease, wouldn't it be more informative to perform these analyses under pathological or stress conditions, such as media (w/o FBS) +/- Aβ, or co-culture with AD neurons or other cell types, as the authors mention in the discussion?

We thank the reviewer for this suggestion. We have replaced *Figure S1E* by bar plots comparing the abundance of oleic acid (18:1)-containing lipid classes between vehicle and oleic acid (**Figure S3A**). We have also included these data in **Supplementary Table 21**. We indeed see that changes in the overall lipid composition with oleic acid are more prominent in the buoyant fraction than the whole cell. However, specific lipid classes like neutral lipids do change in both the buoyant fraction and whole cell. We have adjusted the text to incorporate these new analyses (lines 149-154).

We also appreciate the point about the use of oleic acid. We have included a full statistical analysis (**Supplementary Table 2**) to show all the details of our analysis rather than the summary. We agree that oleic acid stimulation is not a “basal” condition. Treatment with oleic acid is a standard paradigm adopted for years in the lipid droplet field[8 9]. Oleic acid has been known to have minimal lipotoxicity and ER stress[10]. We agree this is a condition chosen for experimental convenience. Even with oleic acid supplementation, it takes over 40 million astrocytes per biological replicate to achieve efficient lipid droplet extractions. For genotypes with fewer lipid droplets (like *APOE3*), lipid droplet extraction is far less efficient without oleic acid.

We have attempted to use AD-relevant stimuli like amyloid-β to induce lipid droplets in astrocytes. Interestingly, in contrast to our own and others' results with microglia[5,11,12], amyloid-β treatment does not induce a robust lipid droplet accumulation phenotype in astrocytes, suggesting differences in the biology of each cell type (data below, *not included in manuscript*). Co-culture is a great idea to recapitulate a more physiological context. We envision such studies as the subject of future work.

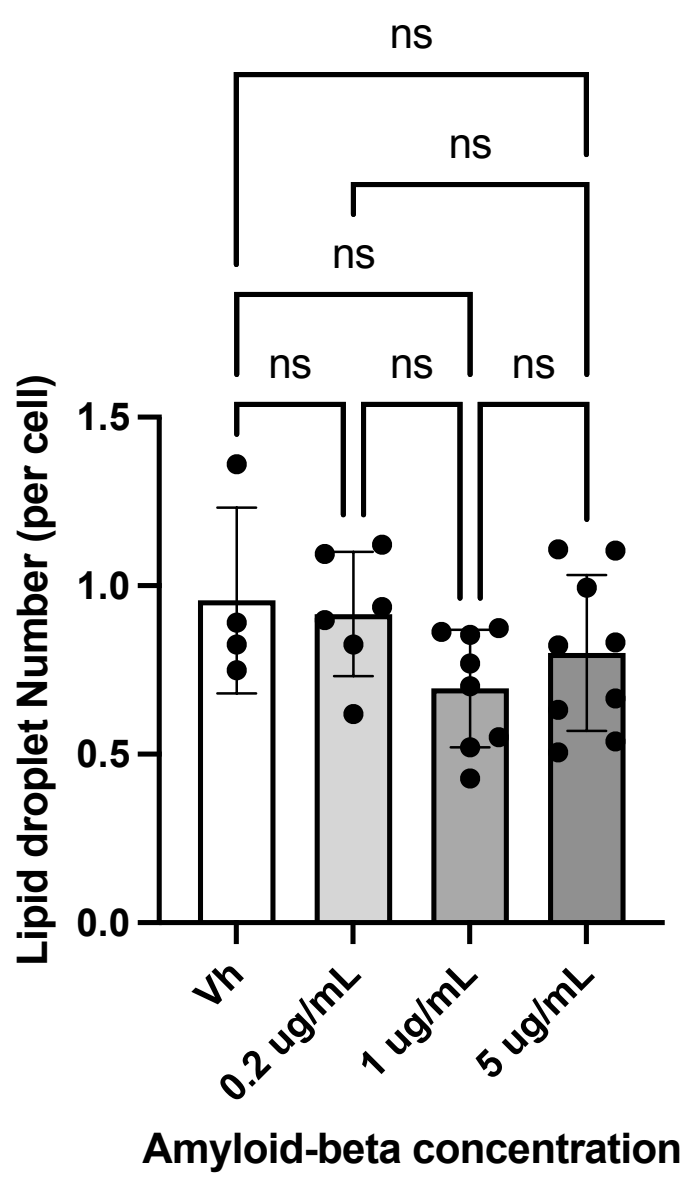

The number of lipid droplets in APOE2 astrocytes is higher than in APOE4 under the vehicle conditions, but lower in oleic acid conditions. How do the authors interpret these data? This also suggests that the lipid droplet profiles under oleic acid and vehicle treatments may differ.

Thank you for noting this. Although we see differing amounts of lipid droplets between *APOE2* and *APOE4* lipid droplets at baseline, the treatment with oleic acid is so strong that it drives high levels of lipid droplet formation in both genotypes. *APOE4* cells may exhibit increased oleic acid uptake to drive lipid droplet formation or increased activity in lipid droplet synthesis when presented with exogenous substrates. Our manuscript now includes a detailed characterization of the lipid changes in the whole cell and buoyant fraction with and without oleic acid treatment across all genotypes (**Figure S3A**). We do not observe genotype-specific differences in lipid composition changes upon oleic acid treatment.

The omics analyses were performed by combining two isogenic lines. However, crucial information is missing, making the data very confusing. For each column in the heatmaps and plots (e.g., Figure 1F, Figure 4A, Figure S1E, Figure 2B, Figure S3), the origin of the sample (which cell line) should be provided. The number of samples per isoform varies, and some isoforms are absent in certain representations. This is particularly concerning

given that the cell lines have different sexes; it is necessary to check for line-specific differences, even if there is only one line per sex. Was the data from the two other lines normalized separately before comparison? Was each column generated by pooling multiple cultures or wells?

We thank the reviewer for bringing this to our attention. We have now labeled all the cell line information in all heatmaps. The aim of our study is to define a lipid droplet proteome and lipidome from human iPSC-derived astrocytes. We acknowledge that the parental lines we used (KOLF2.1J and BIONi037) come from donors with different sex and different genetic backgrounds, and this impacts the proteomic signature at both lipid droplet and whole cell level. We observed, however, that *APOE* genotype was a stronger driver of variance within our buoyant fraction proteomics data than parental line (data below and added to **Figure S5A**).

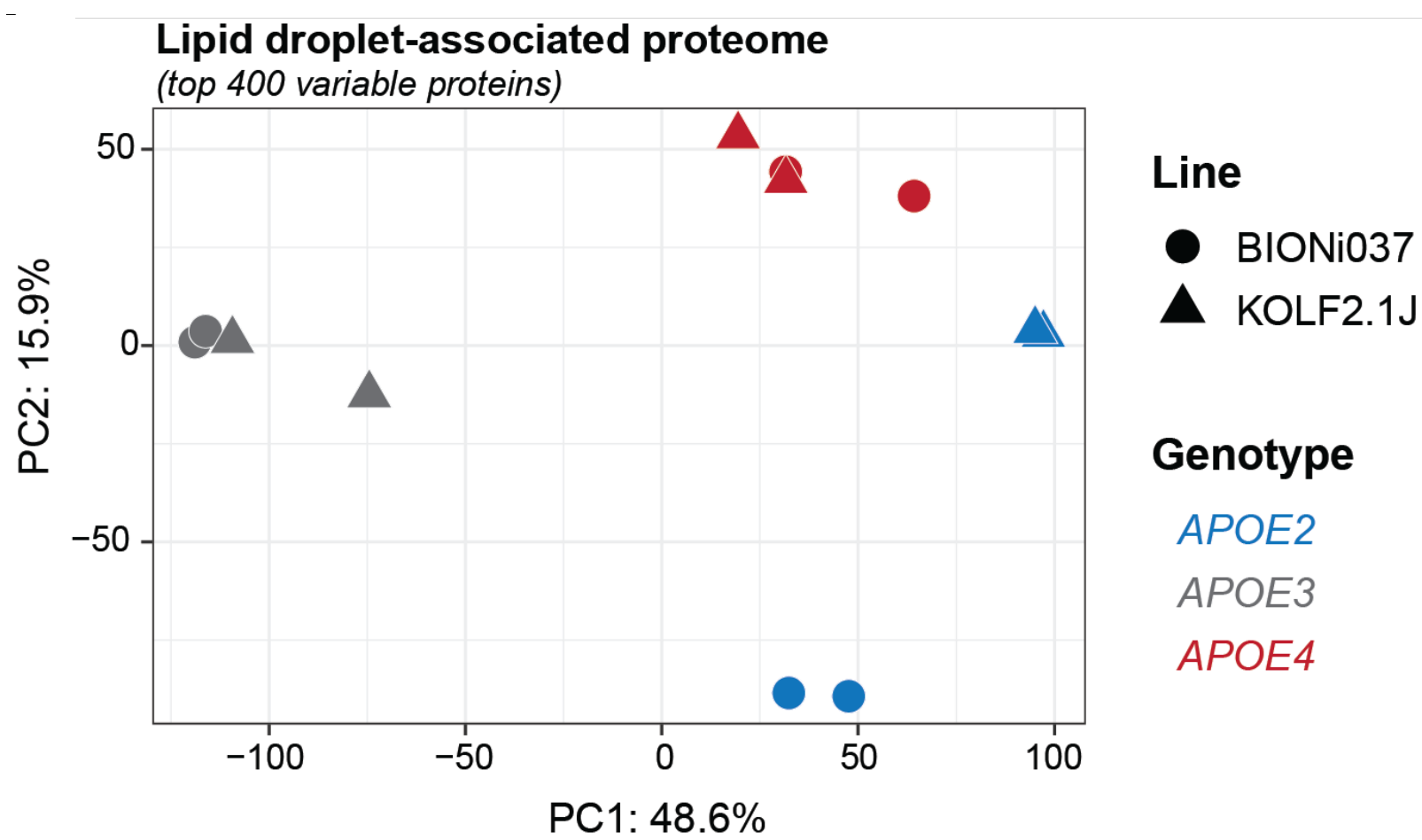

For our analyses of astrocytic lipid droplets and genotype-specific lipid droplets we ensured to balance the number of samples from each line to not bias the data towards one parental line. We then used an extremely stringent analysis paradigm of only including proteins present in both lines of a shared *APOE* genotype to ensure that other sources of variation were not driving any effects we observed. Importantly, this approach of focusing on shared molecular signatures between parental lines has been used before[6].

What is the criterion for using the KOLF2.1J line in some experiments and the BIONi037 line in others? Are the results from both lines shown alternately? A direct comparison of the results between the lines may be needed, as they are from different sexes, also, given that the two cell lines seem to be maintained and differentiated using different methods (e.g., Matrigel vs. Geltrex for culture, and distinct astrocyte differentiation protocols).

We have performed all experiments in both isogenic sets. Across all bar plot representations, we have visualized the data points with a symbol shape code to identify parental origin (square – KOLF2.1J; circle – BIONi037). Due to space constraints, we have

included data from KOLF2.1J in the main text and BIONi037 in the supplement. We have attempted to validate all our findings in astrocytes from both lines. Details of the parental line used in each experiment are provided in the corresponding figure legend.

Minor Points

The manuscript contains several strong claims that lack sufficient experimental support (e.g., Line 218, Line 262). For example, the interpretation in Line 333 regarding the role of immune proteins is also oversimplified, as inflammation can be beneficial or detrimental. Line 344: The observed lipophagy impairment could result from a general defect in the autophagy machinery in APOE4 astrocytes, which has been reported in multiple studies. The authors claim to provide a "mechanistic basis" (Line 306), but no precise mechanisms are elucidated.

We thank the reviewer for this comment. We agree that the reduced lipophagy we observe could arise from general autophagy defects associated with the *APOE4* genotype. Our revised experiments using chemical inhibitors of lipolysis and lipophagy (**Figure 5** and **Figures S7**-**S8**) provide a basis to functionally validate our proteomics observations suggesting arrested lipophagy flux of *APOE4* lipid droplets. Therefore, our proteome characterization of lipid droplets pointed towards potential genotype-specific dynamics of these organelles that we have been able to confirm using additional cell-based assays. However, as the reviewer highlights, our study does not provide a mechanistic understanding of how these *APOE*-dependent changes in lipid droplet dynamics occur. We have adjusted our language to avoid overinterpreting our results (lines 427-430).

Can these observations be confirmed in human AD brains? At a minimum, demonstrating the distinct expression of lipid-associated proteins between APOE isoforms would be valuable.

Thank you for suggesting this. We used single-nuclei transcriptomic data on post-mortem brains from 4 studies to explore whether we could identify genotype-specific expression of transcripts encoding lipid droplet proteins in astrocytes[13-16]. Although these are transcriptional data and characterized at a whole cell level, we were still able to observe subsets of *APOE* genotype-specific lipid droplet proteins that were expressed highly in human samples carrying their respective *APOE* allele (**Figure S5D**). For example, the *APOE2*-specific lipid droplet protein ADK, which is involved in responding to toxic substances, was enriched in *APOE2* carriers; whereas the *APOE4*-specifc proteins RAB8A and MYO1E, involved in intracellular trafficking, were mostly enriched in *APOE4* carriers. This analysis also provided supportive evidence in human brain tissues of our findings that the lipid droplet-associated proteins HSD17B8 and DHRSX are differentially abundant across *APOE* genotypes (**Figure S5E**). Lastly, we observed a higher enrichment of *MAP1LC3B*, referred to as the autophagosome marker LC3B, in human samples of *APOE4* carriers compared to *APOE2* (**Figure S5E**), suggesting altered autophagy evident at the

brain tissue level. We have included these new data in the revised manuscript (data below and added to **Figure S5D,E**; **Supplementary Table 14**; and lines 274-286).

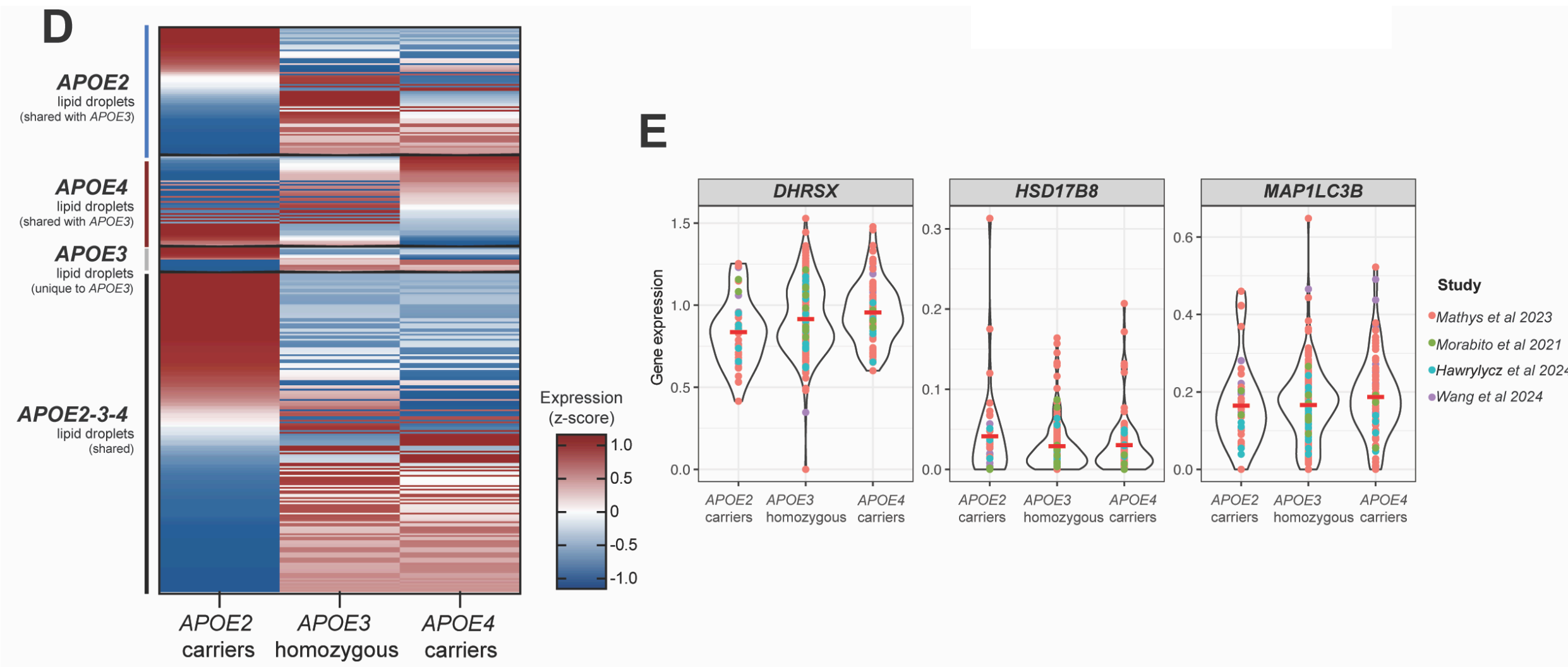

In the abstract, the phrase "impaired lipid droplet clearance, rather than accumulation" is confusing. Isn't accumulation the net result of synthesis and degradation? This phrasing seems paradoxical. Furthermore, as mentioned earlier, lipid droplet accumulation in APOE4 astrocytes is more pronounced with oleic acid treatment than other isoforms. Do the authors attribute this enhanced increase solely to impaired clearance?

Thank you for pointing this out. The reviewer is correct—this phrasing was not precise. We have clarified our language in the abstract to note that lipid droplet clearance is a feature of *APOE4*-associated risk. We have now also directly probed this to provide experimental support to this claim (**Figure 5** and **Figures S7**,**S8**). Lipid droplet accumulation occurs in both *APOE2* and *APOE4* astrocytes compared to *APOE3* astrocytes, but in this work, we show that *APOE4* astrocytes accumulate lipid droplets, at least in part, due to impaired lipophagy. In *APOE2* astrocytes, another biochemical pathway may underlie the accumulation.

Figure 1E: Is the GAPDH signal observed in the ER/mitochondria fraction comparable to the cytosol fraction? In addition, Coomassie blue staining is needed to confirm loading consistency.

Thanks for this comment. Yes, we detected similar GAPDH protein levels between our cytosolic and ER/mitochondria fractions. Indeed, past studies using electron microscopy and subcellular fractionation have shown that GAPDH localizes to other cellular compartments, including the ER and mitochondria [17] [18].

In *Figure 1E*, we probed our buoyant fractions against various organellar markers to confirm the enrichment of lipid droplets and rule out potential contamination from other

cellular compartments. Therefore, the purpose of this experiment was purely qualitative and served as quality control of a protocol that is well-established and has been used by many in the lipid droplet field [19,8,20,21,22]. Using a pilot dataset of iPSC-derived astrocytic buoyant and ER/mitochondria fraction samples loaded at equal protein amounts for mass spectrometry analysis, we confirm differential enrichment of canonical proteins (data below, *not included in manuscript*).

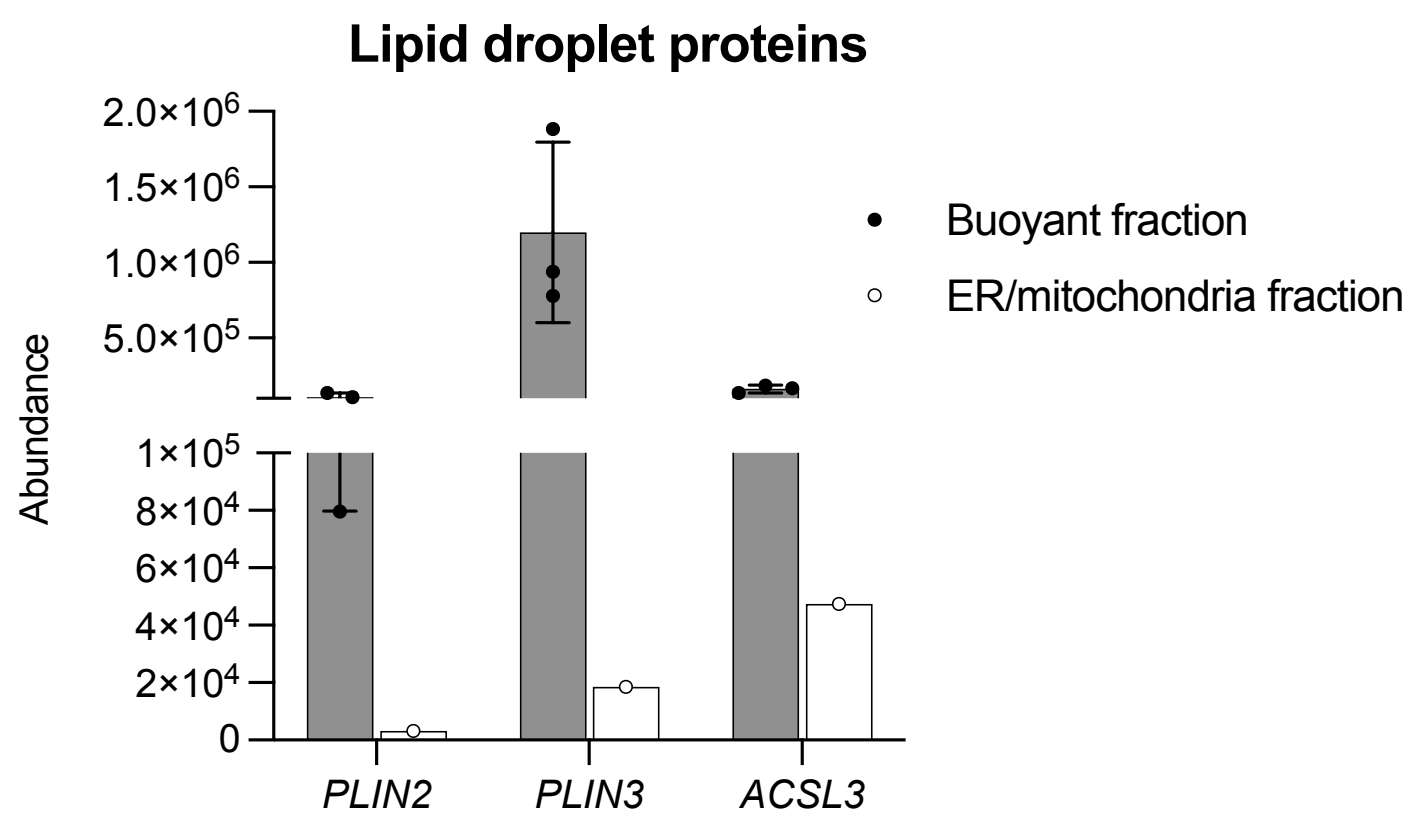

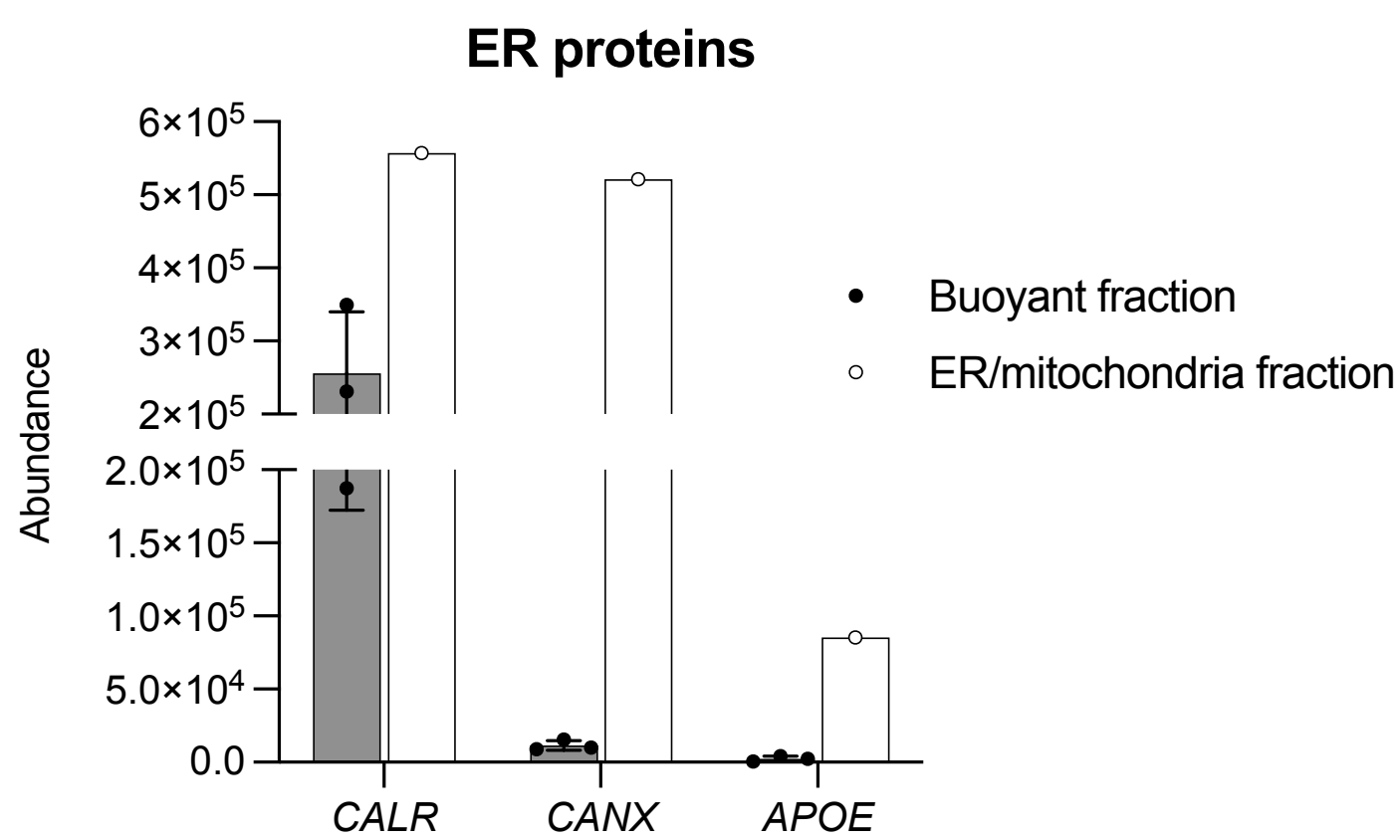

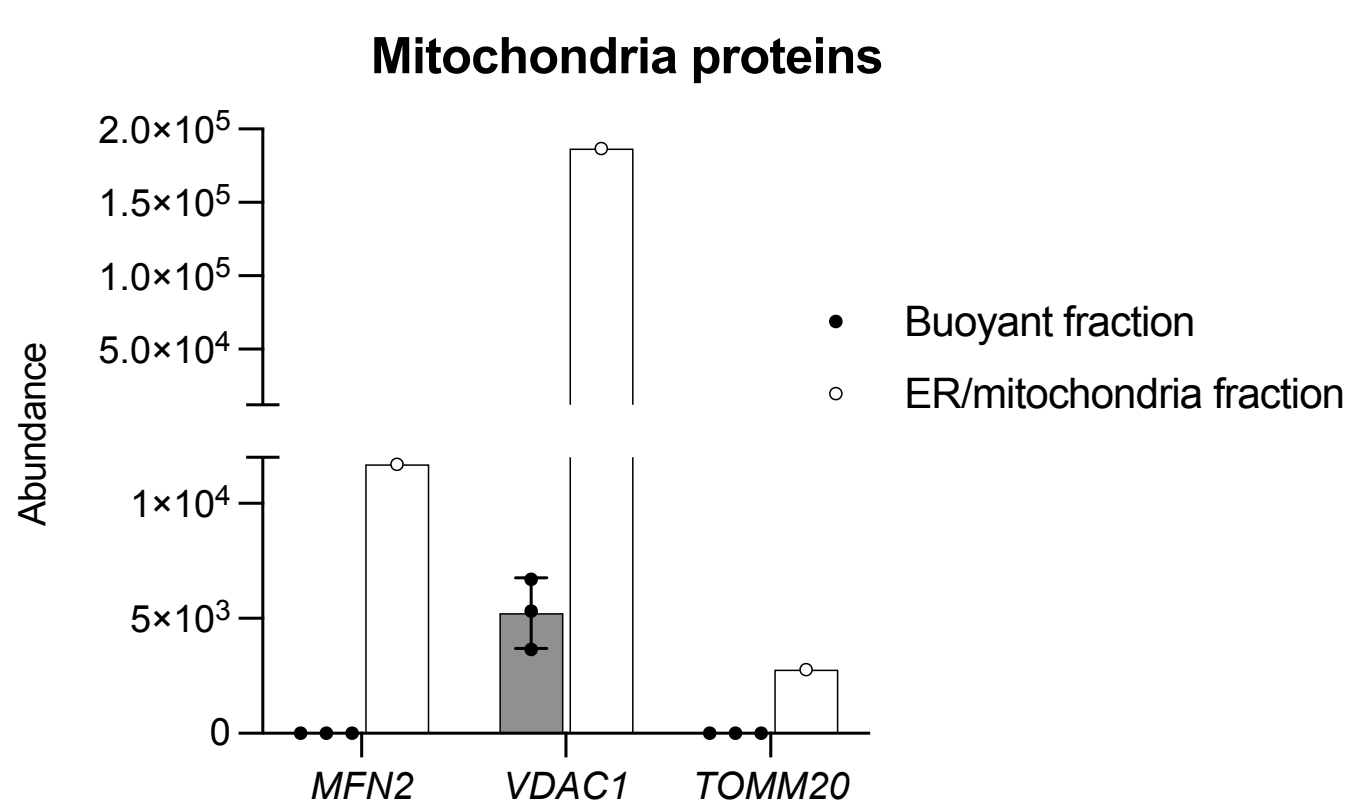

Line 163: The study identifies 403 lipid droplet-specific proteins. (The criteria for defining astrocytic lipid droplet-associated proteins are unclear. Why were the top 2.5% of proteins

selected?) Shouldn't the subsequent comparative analysis (Line 194) be restricted to this set of proteins?

We thank the reviewer for this important comment. We have now clarified the criteria used to define the astrocytic lipid droplet proteome and the rationale for the 2.5% threshold in the revised manuscript (lines 203-205). Please keep in mind that our addition of more replicates in the revised dataset resulted in a smaller number of total proteins as part of the astrocytic lipid droplet proteome (265 instead of 403).

To define the core astrocytic lipid droplet proteome (265 proteins), we applied a stringent cutoff (top 2.5% enrichment) to minimize false positives. This threshold was chosen based on a distribution of the top proteins enriched at least 2 standard deviations above the mean. Importantly, only proteins consistently detected and enriched in buoyant fractions across all *APOE* genotypes were included in this list. This conservative approach ensured that the chosen proteins represent a high-confidence, genotype-independent lipid droplet proteome. As noted in the text, these proteins also included many known lipid droplet proteins from other cell types, reinforcing confidence in our thresholding.

However, restricting the subsequent comparative analysis solely to these 265 proteins would have excluded proteins uniquely enriched in specific *APOE* genotypes. Because such genotype-specific proteins may provide biologically meaningful insight into *APOE*-dependent differences in lipid droplet biology, we performed genotype-specific analyses independently. These proteins were therefore analyzed separately and were not incorporated into the shared 265-protein core set.

We have revised the text to clarify this rationale and the distinction between the core lipid droplet proteome and genotype-specific lipid droplet proteomes (lines 244-251 and 670-685).

For Figures 2H, 3D, 3G, and S2C, quantification of the co-localization ratio (i.e., the proportion of the protein co-localizing with lipid droplets) should be provided to be more informative.

Thank you for this suggestion. We show *APOE* genotype-specific differences in the abundance of the lipid droplet-associated proteins HSD17B8 and DHRSX on lipid droplets (*Figure 3D,E,G,H*). For these analyses we quantified the proportion of lipid droplet volume that is in contact with the target protein of interest. To do this we used the 3D rendering tool in NIS-Elements to obtain the interacting volume between lipid droplets and target protein and then calculate as a percentage how much this volume represents over the total lipid droplet volume in that image. Further details on image acquisition parameters and image analysis can be found in the **Methods** section (lines 747-755) of the manuscript. The purpose of the lipid droplet co-localization with RUFY2 (*Figure 2H*), a novel protein that we have found to be associated to lipid droplets in astrocytes, is to show that they indeed

co-localize with each other in iPSC-derived astrocytes, which biochemically validates our proteomics findings.

Figure 5B, S5A: Are these analyses based on only 3-5 cell images per condition? If so, the sample size is too small to draw robust conclusions.

We thank the reviewer for raising this important point. Although 3–5 representative images per condition are shown, the analysis was performed at the subcellular level and is based on substantially larger sample sizes than the figure presentation may suggest. Images were acquired at 100X resolution, and for each condition we quantified lipid droplets across 10–20 cells per biological replicate, resulting in the analysis of hundreds of lipid droplets per condition. Importantly, statistical analyses were performed using biological replicates (not individual droplets) as the unit of analysis.

This sampling strategy is consistent with prior subcellular co-localization studies [1,2,3], which use comparable cell numbers and imaging-based quantification approaches.

The images in Figure 3G and Figure S5A do not appear to support the finding from Figure 1 that APOE4 astrocytes have more lipid droplets than other isoforms.

We thank the reviewer for this thoughtful observation. The images shown in *Figure 3G* and *Figure S5A* (now *Figure S7A*) are high-magnification (100X) representative fields that were selected to illustrate subcellular localization and co-localization analyses, rather than overall lipid droplet abundance. As such, they do not necessarily reflect the average lipid droplet number per cell across conditions.

The conclusion that *APOE4* astrocytes exhibit increased lipid droplet accumulation is based on the quantitative analysis presented in **Figure 1C** and revised **Figure S1D,F,G**, which was performed across hundreds of cells per condition and multiple biological replicates. These measurements provide a more accurate assessment of lipid droplet abundance than individual representative images. To avoid confusion, we have revised the figure legends to clarify that all our images used for 3D co-localization analyses are intended to illustrate subcellular localization rather than differences in total lipid droplet number.

As mentioned, it is well established that APOE4 astrocytes accumulate more lipids compared to APOE3 astrocytes. To support this, statistical information under vehicle conditions should be provided in Figure 1C. However, in Figure S1D, this trend is not observed in the BIONi037 line.

Thank you for noting this. To replicate previous findings on differential lipid droplet burden between *APOE3* and *APOE4* astrocytes, we have included an additional experiment comparing lipid droplet numbers across all *APOE* genotypes (including *APOE-KO/KO*) in

KOLF2.1J astrocytes that have not been treated with any compounds (**Figure S1C,D**; lines 116-118). As expected, our data showed higher lipid droplet levels in *APOE4* compared to *APOE3* astrocytes, matching previous reports[7 24 25]. For the deep characterization of isolated lipid droplets we have performed in this study, we have stimulated the astrocytes with oleic acid or vehicle control (BSA) to increase lipid droplet amounts and be able to extract them biochemically. Therefore, these treatments are not fully comparable to untreated conditions. Although we see trends retaining the *APOE* genotype-specific differences we observed in untreated cells (more evident in the KOLF2.1 lines than in BIONi037), our oleic acid and vehicle data might result not only from differential lipid droplet accumulation, but also differential lipid droplet biogenesis capacity across genotypes (which is not accounted for in untreated cells). We have also performed additional lipidomics analysis on astrocytes cultured under conditions with different serum withdrawal times and presence or absence of BSA (**Figure S3B,C**; lines 155-161, **Supplementary Table 22**). This comparison revealed important differences in whole cell lipid composition, particularly in neutral lipid levels, dependent on *APOE* genotype and culturing method.

Line 300: The authors state that APOE2 shows a trend toward reduced lipid droplet accumulation under starvation conditions. However, it is not statistically significant, and the description should be adjusted to avoid overinterpretation.

Thank you for pointing this out. We have adjusted the language (lines 424-425).

Reviewer #5 (Remarks to the Author):

I co-reviewed this manuscript with one of the reviewers who provided the listed reports. This is part of the Nature Communications initiative to facilitate training in peer review and to provide appropriate recognition for Early Career Researchers who co-review manuscripts.

Reviewer #6 (Remarks to the Author):

Review of the manuscript entitled APOE genotypes differentially remodel the astrocytic lipid droplet-associated proteome to shape lipid droplet dynamics.
The manuscript is well written, and the results could be of interest to the broader readership of Nature Communicaiton. However, based on the proteomics data presented here I have several concerns that has to be addressed to consider the manuscript for publication.

Major concerns:
The authors write that they are maximizing the number of lipid droplets in the astrocytes by stimulation with Oleic acid. Could this treatment influence the findings later?

The authors claim that the treatment with Oleic acid is not "substantially altering the overall lipid composition of the cell". The figure S1E contains quite a lot of changes, so what is this statement based on? Also, why does the Figure not show APOE3 without the treatment? The figure should be reorganized so that the Vehicle and the Oleic acid treatment for each cell line is next to each other to allow visual evaluation of the changes associated with the treatment.

We thank the reviewer for these helpful suggestions. To improve clarity, we have replaced *Figure S1E* with bar plots directly comparing lipid class abundances between vehicle- and oleic acid–treated conditions and reorganized the presentation so that treatments are shown side-by-side for each cell line (**Figure S3A**; lines 149-155). In addition, we have revised the text to more accurately reflect that oleic acid treatment does induce measurable changes in lipid composition, rather than stating that it does not substantially alter it. We have also included all oleic acid lipid data in **Supplementary Table 21**.

Our updated analysis shows that the effects of oleic acid are more pronounced in the buoyant fraction compared to whole cell extracts when considering all lipid classes collectively. For neutral lipid species, however, changes are observed in both the buoyant fraction and whole cell samples following treatment.

Regarding *APOE3*, the vehicle-treated buoyant fractions could only be obtained from the BIONi037 line, as the *APOE3* KOLF2.1J line produced very few lipid droplets under basal conditions, limiting our ability to isolate sufficient material for analysis. Please note that no vehicle-treated buoyant fractions are included for *APOE3* in the KOLF2.1J line.

The authors do not include how many replicates they are using for the analysis. From the supplementary tables it looks like they are using very few replicates, and maybe even only from technical replicates. This must be explained better. If it is only technical replicates, The study is not publishable as proteomics and especially DIA proteomics require at least 3 biological replicates. This reviewer has downloaded some of the data raw files and search them in Spectronaut. Here it is evident that some of the replicates are very far from each other in the number of proteins/peptides they identify. For example, the result from APOE3 droplet 1 give 2255 proteins whereas APOE droplet 2 give 4845 proteins using the same settings, indicating a very poor reproducibility in the enrichment and whole analysis. Therefore at least 3 biological replicates MUST be used for this analysis, and more replicates if the raw data files give so large difference in number of proteins.

We thank the reviewer for highlighting the importance of clearly reporting biological replicates and data reproducibility. We apologize for the lack of clarity in the original manuscript. All replicates presented in our study are biological replicates. Individual cell lines were plated, grown, treated, and extracted separately for each replicate. In our study, we used four biological replicates for the *APOE2* and *APOE4* genotypes, while only two biological replicates were initially available for *APOE3* due to sample loss caused by

instrumentation issues during acquisition. We agree that the limited number of *APOE3* biological replicates is suboptimal for robust DIA proteomics analysis.

To address this concern, we have now generated additional *APOE3* biological replicates (bringing the total to four) and reprocessed the dataset accordingly. The updated analysis demonstrates improved consistency across replicates, and the revised manuscript now clearly specifies the number and nature of replicates used in all experiments.

Regarding the variability noted by the reviewer (e.g., differences in protein identifications between *APOE3* runs), we acknowledge that the initial dataset showed variability likely due to a combination of sample handling and instrument performance during the affected runs. With the inclusion of additional biological replicates and re-analysis under consistent conditions, we observe substantially improved reproducibility and more stable protein identification numbers across samples.

We have revised the **Methods** and **Results** sections to explicitly describe the number of biological replicates per condition and the updated data processing workflow and quality control measures lines

We agree with the reviewer that at least three biological replicates are essential for reliable proteomics analysis, and we have now ensured that this standard is met (and exceeded) for all sample groups in the revised manuscript.

The authors used DIA on an Eclipse Mass spectrometer, that can run 14 Hz at 30.000 Resolution. The loop control which I believe is the time between each MS is set to 3 seconds. The authors are not including important details on how the data are processed in Spectronaut. Was the quantitation performed on the MS1 or the MS2 level? If the last strategy was used, then how many peaks over the elution profile were obtained? Enough to make a solid quantification? What are the CVs? The authors write that they have 75 windows per scan cycle with 8 Da windows and at a scan rate of 14 Hz the completion of the 75 windows will take more than 5 seconds. This will not give enough points over the elution profile to use MS2 based quantitation unless very bad chromatography. Such details need to be present in the manuscript and the setting for the DIA must be justified.

We thank the reviewer for raising these important points regarding DIA acquisition and data processing. We confirm that quantification was performed at the MS2 level. The DIA method was operated with a loop control of 3 seconds, and typical peptide elution peak widths were approximately 30 seconds. This results in roughly 10 data points across each chromatographic peak, which is sufficient for reliable area-under-the-curve (AUC) quantification.

Regarding the scan cycle, although 75 variable windows (8 Da) were used across the 400–1000 m/z range, the instrument was operated using automatic maximum injection time. This allows the instrument to dynamically adjust dwell times such that all windows are

completed within the defined 3-second loop control. This behavior can be verified directly in the RAW files and ensures that the effective cycle time does not exceed the reported loop control.

We acknowledge that the total ion chromatograms appear relatively sparse; however, this is expected given the low protein input from the lipid droplet (subcellular) fraction. Importantly, extracted ion chromatograms show sufficient signal intensity and well-defined peak shapes to support robust MS2-based quantification.

We have revised the **Methods** section (lines 632-639) to clarify the DIA acquisition parameters, the use of MS2-based quantification in Spectronaut, and the justification for cycle time and sampling density. This acquisition and analysis workflow has also been previously described and validated[26] where it has been successfully applied in similar contexts.

The authors write that they are using p-values of <0.1? Why such a high p-value? Normally in proteomics 0.05 is used and there needs to be a very good explanation for using 0.1 such as tissue samples or samples from individuals. Here the authors are using iPSC lines which is not enough to justify p<0.1.

We thank the reviewer for raising this important point. We use the threshold of p<0.1 in our comparison of the effects of our oleic acid treatment and vehicle (BSA) conditions on the iPSC-derived astrocyte lipid droplet proteome. Please do note that our study design incorporates isogenic iPSC lines derived from two independent individuals, introducing an additional layer of biological variability compared to studies using a single cell line. Our goal was to identify effects that are consistent across genetic backgrounds rather than specific to a single line. To account for this inter-individual variability and avoid over-stringency that could mask biologically meaningful trends shared across donors, we applied a threshold of p < 0.1. We note that this approach is often used in studies where capturing consistent trends across heterogeneous biological backgrounds is a priority. This p-value allows us to account for changes in the treatment condition more comprehensively than the stringent p-value of 0.05 and avoid false negatives. Even with the more permissive p-value <0.1 threshold we do not see significant differences in the lipid droplet proteomes of oleic acid and vehicle treated cells.

We have now clarified this rationale in the **Methods** section (lines 659-669).

Out of the 18 Oleic acid specific proteins there are 3 transcription factors. Can the authors explain this? Why would transcription factors be present in lipid droplets? Has this been observed before? Also, the Supplementary Table 1 I assume is excel sheet 1. BF_OAvsVeh? This should be labeled better.

We thank the reviewer for these helpful comments. Transcription factors have previously been reported to associate with lipid droplets, where they are thought to function as metabolic regulators or to be transiently sequestered [8 27,28]. We have now incorporated this context into the **Discussion** section (lines 442-446) to better explain the potential presence of transcription factors in this compartment.

Following reanalysis of the dataset and after including additional *APOE3* biological replicates, we no longer detect statistically significant differentially abundant proteins between oleic acid and control conditions. As a result, the previously noted transcription factors are no longer classified as oleic acid–specific. We have updated **Supplementary Table 2** (BSA vs oleic acid comparison) accordingly to reflect this revised analysis.

We also agree that the labeling of the supplementary tables was unclear. We have revised the table names and descriptions to ensure that all comparisons (including BF_OA vs Vehicle) are clearly defined and easily interpretable. In addition, we provide a README tab describing the contents and structure of all supplementary tables to further improve clarity.

There needs to be a dedicated Table with all identified proteins from the Spectronaut DIA analysis, including how many peptides that are identified per protein and abundances in all individual replicates for all the cell lines and lipid droplet enrichments and standard deviations including p-values and adjusted p-values must be included. Proteins that are only identified and quantified using 1 single peptide should be eliminated from the analysis. From the Spectronout searches of APOE2 droplet 1 and 2 and APOE3 droplet 1 and 2 there are significant amounts of the proteins that are identified with only one peptide (+12% or more). Having only one peptide in DIA is not good enough for a solid identification and normally in proteomics one would use 2 or more peptides for quantitation. At least 2-3 peptides in DIA should be present to make a valid quantitation. Furthermore, the number of missing values between the samples to compare is very high which is indicative of either very different samples or poor analysis in LC-MSMS. Imputation is not a valid method with very few replicates.

Thank you for this detailed and helpful suggestion. We have revised our analysis and supplementary materials accordingly to address these concerns.

During data processing, we excluded all proteins identified by a single peptide prior to downstream analysis, in line with established best practices for DIA-based proteomics. We have now included peptide count information for all identified proteins in **Supplementary Table 2 (column D)**. We have also expanded the supplementary tables to provide detailed quantitative information across all biological replicates, including protein abundances (**Supplementary Tables 3-6**).

Regarding missing values, we note that the observed missingness is largely attributable to the subcellular fractionation workflow, which can introduce variability in protein detection

between fractions. To avoid introducing bias, we restricted imputation to fractionation datasets where sufficient replicate numbers were available (≥10 replicates per condition) and did not apply imputation to datasets with low replicate counts.

These updates have been incorporated into the **Methods** (lines 650-651) and supplementary information to improve transparency and reproducibility.

In Figure 2A the authors describe their workflow to identify "the human iPSC-derived astrocyte lipid droplet-associated proteome". But from the lists in the supplementary tables, it is a bit difficult to see how the authors came to these numbers? Please make a thorough explanation here.

Thank you for this suggestion; we have now clarified this in the revised **Methods** section.

To generate these lists, we first screened all UniProt IDs against the Contaminant Repository database[29]. Proteins identified in more than 50% of human studies were classified as common contaminants (**Supplementary Table 1**) and excluded from further analysis. To identify proteins present in both the buoyant and cytosolic fractions, we applied z-score normalization to each dataset and calculated the mean z-score across oleic acid–treated samples for each protein. We then fitted a linear model relating the mean z-scores of the buoyant and cytosolic fractions. For proteins detected in the buoyant fraction, residuals from this model were calculated and ranked to assess enrichment relative to the cytosolic fraction. The top 2.5% of residuals (corresponding to approximately two standard deviations above the mean) were defined as astrocyte lipid droplet–associated proteins. To identify proteins detected exclusively in the buoyant fraction, we ranked proteins by their mean z-score (highest to lowest) and selected those with a z-score > -1.

We have also included detailed tables of Z-scores and residual ranks in **Supplementary Tables 5 and 6** to enable readers to easily reproduce these steps.

In line 125-131 the authors write that the 18 proteins specific in the Oleic acid treated fraction is probably because there are more lipid droplets in these cells. However, in figure 1C there are very large differences between the treated and non-treaded samples with respect to number of lipid droplets, and it is a bit strange that such a large difference only results in a difference of 18 proteins? Can the authors comment on this? How many of the 18 proteins were identified by only 1-2 peptides in the DIA analysis?

Thank you for raising this point. We apologize for the lack of clarity in the original text. Following the addition of two *APOE3* samples and re-analysis of the dataset with stricter filtering criteria (excluding proteins identified by a single peptide) we no longer observe a

distinct set of proteins uniquely present in the oleic acid–treated fraction compared to controls.

These updated results indicate that, although oleic acid treatment increases the number of lipid droplets, it does not substantially alter their proteomic composition. We have revised the text accordingly to avoid overstating differences between conditions (lines 168-171). In addition, we have included data comparing vehicle and oleic acid treatments in **Supplementary Table 2** to ensure transparency and allow readers to assess the data directly.

Line 172-175: The authors say that RNA biology has not been linked to lipid droplets before? This is not entirely true, as the spliceosome has previously been linked to lipid droplets (e.g. PMID: 24333419).

Thank you for bringing this to our attention this. We have adjusted our phrasing and included this reference (lines 220-221).

Line 196-205: The data used for generating the lists of protein in lipid droplets from APOE2-4 are highly questionable as it seems that there have only been analyzed technical replicates. This is not enough for statistical analysis and is normally not valid within the field. At least 3 biological replicates must be included in the study, and a proper statistical test must be applied. Single peptide proteins (one-hit wonders) are also not used in the field, especially not for quantitative DIA analyses.

Thank you for this comment. We apologize for the lack of clarity and have revised the manuscript to better describe our experimental design and analysis.

To clarify, all samples are biological replicates, not technical replicates. Each replicate represents an independent iPSC line that was cultured, treated, and processed separately, and therefore captures biological variability rather than technical repetition.

We used four biological replicates for the *APOE2* and *APOE4* samples. While initial analyses of *APOE3* were limited due to instrumentation failures, we have now added additional APOE3 biological replicates to ensure adequate statistical power across all groups. In addition, we have reanalyzed the dataset using stricter filtering criteria and have excluded proteins identified by a single peptide ("one-hit wonders"), in line with current best practices for DIA-based proteomics.

These updates have been incorporated into the revised manuscript, and appropriate statistical analyses have now been applied.

References

1. Das, D., Sharma, M., Gahlot, D., Nia, S.S., Gain, C., Mecklenburg, M., Zhou, Z.H., Bourdenx, M., Thukral, L., Martinez-Lopez, N., and Singh, R. (2024). VPS4A is the selective receptor for lipophagy in mice and humans. Mol Cell *84*, 4436-4453 e4438. 10.1016/j.molcel.2024.10.022.
2. Kajiwara, K., Osaki, H., Gressies, S., Kuwata, K., Kim, J.H., Gensch, T., Sato, Y., Glorius, F., Yamaguchi, S., and Taki, M. (2022). A negative-solvatochromic fluorescent probe for visualizing intracellular distributions of fatty acid metabolites. Nat Commun *13*, 2533. 10.1038/s41467-022-30153-6.
3. Parcon, P.A., Balasubramaniam, M., Ayyadevara, S., Jones, R.A., Liu, L., Shmookler Reis, R.J., Barger, S.W., Mrak, R.E., and Griffin, W.S.T. (2018). Apolipoprotein E4 inhibits autophagy gene products through direct, specific binding to CLEAR motifs. Alzheimers Dement *14*, 230-242. 10.1016/j.jalz.2017.07.754.
4. Lima, D., Hacke, A.C.M., Inaba, J., Pessoa, C.A., and Kerman, K. (2020). Electrochemical detection of specific interactions between apolipoprotein E isoforms and DNA sequences related to Alzheimer's disease. Bioelectrochemistry *133*, 107447. 10.1016/j.bioelechem.2019.107447.
5. Stephenson, R.A., Sepulveda, J., Johnson, K.R., Lita, A., Gopalakrishnan, J., Acri, D.J., Beilina, A., Cheng, L., Yang, L.G., Root, J.T., et al. (2025). Triglyceride metabolism controls inflammation and microglial phenotypes associated with APOE4. Cell Rep *44*, 115961. 10.1016/j.celrep.2025.115961.
6. Feringa, F.M., Koppes-den Hertog, S.J., Wang, L.Y., Derks, R.J.E., Kruijff, I., Erlebach, L., Heijneman, J., Miramontes, R., Pompner, N., Blomberg, N., et al. (2025). The Neurolipid Atlas: a lipidomics resource for neurodegenerative diseases. Nat Metab *7*, 2142-2164. 10.1038/s42255-025-01365-z.
7. Feringa, F.M., Hertog, S.J.K., Wang, L., Derks, R.J.E., Kruijff, I., Erlebach, L., Heijneman, J., Miramontes, R., Pömpner, N., Blomberg, N., et al. (2024). The Neurolipid Atlas: a lipidomics resource for neurodegenerative diseases uncovers cholesterol as a regulator of astrocyte reactivity impaired by ApoE4. bioRxiv. 10.1101/2024.07.01.601474.
8. Bersuker, K., Peterson, C.W.H., To, M., Sahl, S.J., Savikhin, V., Grossman, E.A., Nomura, D.K., and Olzmann, J.A. (2018). A Proximity Labeling Strategy Provides Insights into the Composition and Dynamics of Lipid Droplet Proteomes. Developmental cell.
9. Mejhert, N., Gabriel, K.R., Frendo-Cumbo, S., Krahmer, N., Song, J., Kuruvilla, L., Chitraju, C., Boland, S., Jang, D.K., von Grotthuss, M., et al. (2022). The Lipid Droplet Knowledge Portal: A resource for systematic analyses of lipid droplet biology. Dev Cell *57*, 387-397.e384. 10.1016/j.devcel.2022.01.003.
10. Salvado, L., Coll, T., Gomez-Foix, A.M., Salmeron, E., Barroso, E., Palomer, X., and Vazquez-Carrera, M. (2013). Oleate prevents saturated-fatty-acid-induced ER stress, inflammation and insulin resistance in skeletal muscle cells through an AMPK-dependent mechanism. Diabetologia *56*, 1372-1382. 10.1007/s00125-013-2867-3.

11. Prakash, P., Manchanda, P., Paouri, E., Bisht, K., Sharma, K., Rajpoot, J., Wendt, V., Hossain, A., Wijewardhane, P.R., Randolph, C.E., et al. (2025). Amyloid-β induces lipid droplet-mediated microglial dysfunction via the enzyme DGAT2 in Alzheimer's disease. Immunity. 10.1016/j.immuni.2025.04.029.
12. Haney, M.S., Pálovics, R., Munson, C.N., Long, C., Johansson, P.K., Yip, O., Dong, W., Rawat, E., West, E., Schlachetzki, J.C.M., et al. (2024). APOE4/4 is linked to damaging lipid droplets in Alzheimer's disease microglia. Nature.
13. Mathys, H., Peng, Z., Boix, C.A., Victor, M.B., Leary, N., Babu, S., Abdelhady, G., Jiang, X., Ng, A.P., Ghafari, K., et al. (2023). Single-cell atlas reveals correlates of high cognitive function, dementia, and resilience to Alzheimer's disease pathology. Cell *186*, 4365-4385.e4327. 10.1016/j.cell.2023.08.039.
14. Wang, C., Acosta, D., McNutt, M., Bian, J., Ma, A., Fu, H., and Ma, Q. (2024). A single-cell and spatial RNA-seq database for Alzheimer's disease (ssREAD). Nat Commun *15*, 4710. 10.1038/s41467-024-49133-z.
15. Morabito, S., Miyoshi, E., Michael, N., Shahin, S., Martini, A.C., Head, E., Silva, J., Leavy, K., Perez-Rosendahl, M., and Swarup, V. (2021). Single-nucleus chromatin accessibility and transcriptomic characterization of Alzheimer's disease. Nat Genet *53*, 1143-1155. 10.1038/s41588-021-00894-z.
16. Hawrylycz, M., Kaplan, E.S., Travaglini, K.J., Gabitto, M.I., Miller, J.A., Ng, L., Close, J.L., Hodge, R.D., Long, B., Mollenkopf, T., et al. (2024). SEA-AD is a multimodal cellular atlas and resource for Alzheimer's disease. Nat Aging *4*, 1331-1334. 10.1038/s43587-024-00719-8.
17. Tarze, A., Deniaud, A., Le Bras, M., Maillier, E., Molle, D., Larochette, N., Zamzami, N., Jan, G., Kroemer, G., and Brenner, C. (2007). GAPDH, a novel regulator of the pro-apoptotic mitochondrial membrane permeabilization. Oncogene *26*, 2606-2620. 10.1038/sj.onc.1210074.
18. Mazzola, J.L., and Sirover, M.A. (2003). Subcellular localization of human glyceraldehyde-3-phosphate dehydrogenase is independent of its glycolytic function. Biochim Biophys Acta *1622*, 50-56. 10.1016/s0304-4165(03)00117-x.
19. Peterson, C.W.H., Deol, K.K., To, M., and Olzmann, J.A. (2021). Optimized protocol for the identification of lipid droplet proteomes using proximity labeling proteomics in cultured human cells. STAR Protocols *2*, 100579. https://doi.org/10.1016/j.xpro.2021.100579.
20. Brasaemle, D.L., and Wolins, N.E. (2016). Isolation of Lipid Droplets from Cells by Density Gradient Centrifugation. Current Protocols in Cell Biology *72*, 3.15.11-13.15.13. https://doi.org/10.1002/cpcb.10.
21. Mathiowetz, A.J., Meymand, E.S., Parlakgul, G., van Hilten, N., Torres, E.F., Artico, L.L., Deol, K.K., Lange, M., Pang, S.P., Doubravsky, C.E., et al. (2026). CLCC1 promotes hepatic neutral lipid flux and nuclear pore complex assembly. Nature *652*, 462-470. 10.1038/s41586-025-10064-4.
22. Wu, L., Wang, J., Wang, Y., Yang, J., Yao, Y., Wang, Y., Huang, D., Hu, Y., Xu, X., Wang, R., et al. (2026). CLCC1 governs ER bilayer equilibration to maintain lipid homeostasis. Nature *652*, 471-480. 10.1038/s41586-026-10161-y.

23. Chung, J., Park, J., Lai, Z.W., Lambert, T.J., Richards, R.C., Zhang, J., Walther, T.C., and Farese, R.V., Jr. (2023). The Troyer syndrome protein spartin mediates selective autophagy of lipid droplets. Nat Cell Biol *25*, 1101-1110. 10.1038/s41556-023-01178-w.
24. Sienski, G., Narayan, P., Bonner, J.M., Kory, N., Boland, S., Arczewska, A.A., Ralvenius, W.T., Akay, L., Lockshin, E., He, L., et al. (2021). APOE4 disrupts intracellular lipid homeostasis in human iPSC-derived glia. Science Translational Medicine.
25. Farmer, B.C., Kluemper, J., and Johnson, L.A. (2019). Apolipoprotein E4 Alters Astrocyte Fatty Acid Metabolism and Lipid Droplet Formation. Cells.
26. Reilly, L., Lara, E., Ramos, D., Li, Z., Pantazis, C.B., Stadler, J., Santiana, M., Roberts, J., Faghri, F., Hao, Y., et al. (2023). A fully automated FAIMS-DIA mass spectrometry-based proteomic pipeline. Cell Rep Methods *3*, 100593. 10.1016/j.crmeth.2023.100593.
27. Mejhert, N., Kuruvilla, L., Gabriel, K.R., Elliott, S.D., Guie, M.A., Wang, H., Lai, Z.W., Lane, E.A., Christiano, R., Danial, N.N., et al. (2020). Partitioning of MLX-Family Transcription Factors to Lipid Droplets Regulates Metabolic Gene Expression. Mol Cell *77*, 1251-1264.e1259. 10.1016/j.molcel.2020.01.014.
28. Stephenson, R.A., Thomalla, J.M., Chen, L., Kolkhof, P., White, R.P., Beller, M., and Welte, M.A. (2021). Sequestration to lipid droplets promotes histone availability by preventing turnover of excess histones. Development *148*. 10.1242/dev.199381.
29. Mellacheruvu, D., Wright, Z., Couzens, A.L., Lambert, J.-P., St-Denis, N.A., Li, T., Miteva, Y.V., Hauri, S., Sardiu, M.E., Low, T.Y., et al. (2013). The CRAPome: a contaminant repository for affinity purification–mass spectrometry data. Nature Methods *10*, 730-736. 10.1038/nmeth.2557.

We thank the reviewers for their thoughtful evaluation of our revised manuscript. We are grateful that Reviewers 1, 3, and 6 found that their concerns had been addressed and that the manuscript was strengthened by the revision. We also thank the co-reviewers recognized through the Nature Communications early-career reviewer initiative. Below, we respond to the remaining concerns raised by Reviewer 4.

Reviewer #4

We thank the reviewer for their careful reading of the revised manuscript and for recognizing our significant efforts made to address the prior concerns. We have further revised the manuscript to avoid overgeneralization, clarify the experimental context of oleic acid treatment, and more clearly describe the proteomics reanalysis and validation strategy.

Comment 1:
The authors state in the main text that, 'With vehicle control treatment, all APOE genotypes of astrocytes still accumulated more lipid droplets than APOE3 astrocytes.' However, this interpretation is heavily biased. In the BIONi037 line, this trend is completely absent (as shown in Figure S1F). I previously pointed out that differences in media and differentiation methods between the cell lines could account for these variations, but the authors largely dismissed this as parental line variation. While it is acceptable to focus on common phenotypes across models, the authors must carefully avoid making biased or overly general statements (such as the one regarding baseline APOE3 lipid droplet accumulation) that are explicitly contradicted by their own data.

We agree with the reviewer that our previous wording was overly broad and did not adequately reflect the line-dependent nature of lipid droplet accumulation under vehicle-treated conditions. We have revised the text to avoid implying that the same baseline pattern is observed across both iPSC-derived astrocyte models.

Specifically, we now state that lipid droplet accumulation under vehicle-treated conditions varies between the two isogenic astrocyte sets, with genotype-dependent differences more apparent in the KOLF2.1J-derived astrocytes than in the BIONi037-derived astrocytes. We also explicitly acknowledge that differences between the KOLF2.1J and BIONi037 models may reflect a combination of parental genetic background, differentiation approach, and culture conditions. We no longer present baseline APOE3 lipid droplet accumulation as a generalizable finding across both models.

Revised text (lines 128-134):

"Under vehicle-treated conditions, lipid droplet accumulation varied across the two isogenic iPSC-derived astrocyte sets, with genotype-dependent differences more apparent in the KOLF2.1J-derived astrocytes (**Figure 1B,C**) than in the BIONi037-

derived astrocytes (Figure S1E,F). These differences may reflect parental genetic background, differentiation method, or culture conditions. Oleic acid treatment robustly increased lipid droplet accumulation across all *APOE* genotypes and both iPSC-derived astrocyte sets (**Figure 1B,C,**S1E-G)."

Comment 2:
The authors acknowledged in the rebuttal that Amyloid-beta treatment did not alter lipid droplet accumulation, whereas OA treatment did. Even though the authors claim there are no significant differences between the vehicle and OA-treated groups, quantitative differences are evident, and the authors' own data demonstrate that OA treatment alters APOE levels. This raises a fundamental question: what physiological or pathological condition does this OA treatment mimic? Even if OA treatment is primarily a strategy of 'experimental convenience' to increase lipid droplet yield, the authors must rigorously discuss what real-world environment this model represents. Furthermore, the reliance on this specific, artificial context must be explicitly stated in both the title and the main text.

We thank the reviewer for raising this important point. We agree that oleic acid treatment should not be interpreted as a direct model of a single physiological or pathological state. Rather, we use oleic acid treatment as a controlled lipid-loading paradigm to increase lipid droplet abundance and obtain sufficient lipid droplet material for organelle-level proteomic and lipidomic analyses.

We have revised the manuscript to make this experimental context more explicit. We now clarify that oleic acid treatment models a lipid-enriched cellular state rather than amyloid exposure or Alzheimer's disease itself. We also note that this approach is commonly used to stimulate lipid droplet biogenesis but remains an artificial experimental condition. Accordingly, the findings should be interpreted as APOE genotype-dependent differences in lipid droplet composition and dynamics under lipid-loading conditions.

We have also revised the Discussion to acknowledge that oleic acid treatment alters *APOE* abundance and that this may influence cellular lipid handling. We now explicitly identify this as a limitation of the study and emphasize that future work will be needed to determine how *APOE* genotype shapes lipid droplet biology under additional disease-relevant stressors.

Revised abstract (lines 31-37):
"Here we show that *APOE* genotype shapes the lipid droplet proteome, lipidome, and degradation dynamics in human induced pluripotent stem cell-derived astrocytes. By comparing oleic acid-treated astrocytes carrying *APOE2*, *APOE3*, or *APOE4*, we find that each variant is associated with distinct lipid droplet proteins and lipids. These molecular differences correspond to genotype-dependent changes in lipophagy, an autophagy-mediated pathway for lipid droplet clearance."

Revised main text (lines 134-139):
“We used oleic acid treatment as a controlled lipid-loading paradigm to increase lipid droplet biogenesis and obtain sufficient lipid droplet material for organelle-level molecular analyses. Although this approach does not model amyloid exposure or Alzheimer’s disease directly. Instead, it represents a lipid-enriched cellular state that facilitates detailed analysis of lipid droplet composition.”

Revised Discussion text (lines 507-514):
“A limitation of this study is that oleic acid treatment is an artificial lipid-loading condition and does not correspond to a single defined physiological or pathological stimulus. Oleic acid treatment also reduced intracellular and secreted APOE levels, indicating that lipid loading can alter *APOE* biology itself. Therefore, our findings should be interpreted in the context of lipid-loaded astrocytes, and future studies will be needed to determine how *APOE* genotype influences lipid droplet composition and dynamics under additional disease-relevant stresses, including inflammatory, oxidative, and amyloid-associated conditions.”

Comment 3:
During the revision process, the authors generated and added two new proteomics samples exclusively to the APOE3 group and re-analyzed the entire dataset. This reanalysis led to different results and interpretations than those in the original manuscript. Batch effects are an inevitable reality when repeating experiments at different times. Therefore, any additional runs must include samples from all experimental groups simultaneously. Adding new samples to only one specific group and merging them with old data is methodologically unacceptable. Given that this post-hoc addition altered the overall conclusions of the study, these results must be viewed with extreme caution, and this fundamental flaw in the experimental design must be addressed.

Response:
We thank the reviewer for raising this important concern. We agree that batch effects and sample-to-sample variability are important considerations in proteomic studies, particularly when additional samples are generated during revision. We did perform batch correction to harmonize additional samples with our existing datasets.

Importantly, the inclusion of the additional *APOE3* samples did not substantively alter the overall human astrocytic lipid droplet-associated proteome presented in Figure 2. The core lipid droplet proteome, including canonical lipid droplet proteins and newly identified astrocytic lipid droplet-associated proteins, remained consistent after reanalysis. Thus, the overall lipid droplet proteome resource was not dependent on the newly added *APOE3* samples.

We also agree that genotype-specific interpretations should not be driven by sample imbalance. We have equal number of samples in each genotype group. We have clarified in the manuscript that our biological interpretation focuses primarily on the

APOE2 versus *APOE4* comparison. This comparison addresses the central contrast between protective and risk-associated *APOE* genotypes and does not rely on emphasizing *APOE3*-specific results from the newly added samples.

In addition, because proteomics datasets can show variability across runs and samples, we did not rely on proteomics alone for our central conclusions. Key genotype-dependent findings were supported by multiple orthogonal approaches, including immunofluorescence and 3D colocalization assays for selected lipid droplet-associated proteins, lipidomics to assess lipid droplet composition, BODIPY-C12 pulse-chase assays to measure lipid droplet turnover, LipiDye™-M imaging to assess lipophagy, and autophagy marker colocalization under lipolysis inhibition and starvation conditions. These independent assays support the conclusion that *APOE2* and *APOE4* lipid droplets differ in composition and turnover dynamics.

We hope these clarifications address the reviewer's concern by making clear that batch correction was already incorporated, that the overall lipid droplet proteome was not substantively changed by inclusion of the *APOE3* samples, and that the genotype-specific conclusions emphasize *APOE2* versus *APOE4* differences supported by multiple independent lines of evidence.
