# Supplementary material for: APOE genotypes differentially remodel the astrocytic lipid droplet proteome to shape lipid droplet dynamics
Source: Nat Commun. 2026 Aug 19;17:9900. doi: 10.1038/s41467-026-76565-6 (PMC13582944; doi:10.1038/s41467-026-76565-6)
Supplement: Supplementary file 3 [file 41467_2026_76565_MOESM3_ESM.pdf]

nature portfolio

Corresponding author(s): Priyanka S. Narayan

Last updated by author(s): 4/30/2026

Reporting Summary

Nature Portfolio wishes to improve the reproducibility of the work that we publish. This form provides structure for consistency and transparency in reporting. For further information on Nature Portfolio policies, see our Editorial Policies and the Editorial Policy Checklist.

Statistics

For all statistical analyses, confirm that the following items are present in the figure legend, table legend, main text, or Methods section.

n/a	Confirmed	
☐	☒	The exact sample size (*n*) for each experimental group/condition, given as a discrete number and unit of measurement
☐	☒	A statement on whether measurements were taken from distinct samples or whether the same sample was measured repeatedly
☐	☒	The statistical test(s) used AND whether they are one- or two-sided<br>*Only common tests should be described solely by name; describe more complex techniques in the Methods section.*
☐	☒	A description of all covariates tested
☐	☒	A description of any assumptions or corrections, such as tests of normality and adjustment for multiple comparisons
☐	☒	A full description of the statistical parameters including central tendency (e.g. means) or other basic estimates (e.g. regression coefficient) AND variation (e.g. standard deviation) or associated estimates of uncertainty (e.g. confidence intervals)
☐	☒	For null hypothesis testing, the test statistic (e.g. *F*, *t*, *r*) with confidence intervals, effect sizes, degrees of freedom and *P* value noted<br>*Give P values as exact values whenever suitable.*
☒	☐	For Bayesian analysis, information on the choice of priors and Markov chain Monte Carlo settings
☒	☐	For hierarchical and complex designs, identification of the appropriate level for tests and full reporting of outcomes
☒	☐	Estimates of effect sizes (e.g. Cohen's *d*, Pearson's *r*), indicating how they were calculated

Our web collection on statistics for biologists contains articles on many of the points above.

Software and code

Policy information about availability of computer code

Data collection	No code was used to collect data
Data analysis	Microscopy data analysis was performed using Nikon Elements Software Colocalization analysis tools. Proteomics analysis was performed using previously published pipelines. Lipidomics analysis was performed using iSODA software (http://isoda.online/). Statistical analysis was performed in GraphPad Prism.

For manuscripts utilizing custom algorithms or software that are central to the research but not yet described in published literature, software must be made available to editors and reviewers. We strongly encourage code deposition in a community repository (e.g. GitHub). See the Nature Portfolio guidelines for submitting code & software for further information.

Data

Policy information about availability of data

All manuscripts must include a data availability statement. This statement should provide the following information, where applicable:

- Accession codes, unique identifiers, or web links for publicly available datasets
- A description of any restrictions on data availability
- For clinical datasets or third party data, please ensure that the statement adheres to our policy

All processed proteomics data is included in supplementary tables. The mass spectrometry proteomics data have been deposited to the ProteomeXchange Consortium via the PRIDE partner repository with the dataset identifier PXD067161. Lipidomics data can be found in supplementary tables and on the neurolipid atlas website via accession NLA_182 and NLA_183.

Research involving human participants, their data, or biological material

Policy information about studies with human participants or human data. See also policy information about sex, gender (identity/presentation), and sexual orientation and race, ethnicity and racism.

Reporting on sex and gender	*Use the terms sex (biological attribute) and gender (shaped by social and cultural circumstances) carefully in order to avoid confusing both terms. Indicate if findings apply to only one sex or gender; describe whether sex and gender were considered in study design; whether sex and/or gender was determined based on self-reporting or assigned and methods used. Provide in the source data disaggregated sex and gender data, where this information has been collected, and if consent has been obtained for sharing of individual-level data; provide overall numbers in this Reporting Summary. Please state if this information has not been collected. Report sex- and gender-based analyses where performed, justify reasons for lack of sex- and gender-based analysis.*
Reporting on race, ethnicity, or other socially relevant groupings	*Please specify the socially constructed or socially relevant categorization variable(s) used in your manuscript and explain why they were used. Please note that such variables should not be used as proxies for other socially constructed/relevant variables (for example, race or ethnicity should not be used as a proxy for socioeconomic status). Provide clear definitions of the relevant terms used, how they were provided (by the participants/respondents, the researchers, or third parties), and the method(s) used to classify people into the different categories (e.g. self-report, census or administrative data, social media data, etc.) Please provide details about how you controlled for confounding variables in your analyses.*
Population characteristics	*Describe the covariate-relevant population characteristics of the human research participants (e.g. age, genotypic information, past and current diagnosis and treatment categories). If you filled out the behavioural & social sciences study design questions and have nothing to add here, write "See above."*
Recruitment	*Describe how participants were recruited. Outline any potential self-selection bias or other biases that may be present and how these are likely to impact results.*
Ethics oversight	*Identify the organization(s) that approved the study protocol.*

Note that full information on the approval of the study protocol must also be provided in the manuscript.

Field-specific reporting

Please select the one below that is the best fit for your research. If you are not sure, read the appropriate sections before making your selection.

[x] Life sciences [ ] Behavioural & social sciences [ ] Ecological, evolutionary & environmental sciences

For a reference copy of the document with all sections, see nature.com/documents/nr-reporting-summary-flat.pdf

Life sciences study design

All studies must disclose on these points even when the disclosure is negative.

Sample size	Our human iPSC-derived astrocytes were derived from two different human iPSC isogenic sets--each containing 3 different lines isogenic at all loci except for APOE. The parental BIONI037A line was derived from a female donor while parental KOLF2.1J was derived from a male donor. We ensured that all data presented was consistent across both isogenic sets of lines and reproduced all data across all lines. No statistical method was used to predetermine sample size but sample sizes were based on previously published research on iPSC-derived astrocytes.
Data exclusions	We did not exclude any data.
Replication	The reproducibility of the experimental findings was supported by the use of two independent isogenic human iPSC-derived astrocyte sets, each carrying the common APOE2, APOE3, or APOE4 genotypes. Key proteomic and lipidomic analyses were performed across both genetic backgrounds, and genotype-specific findings were prioritized only when patterns were consistent across both iPSC lines. Several proteomics findings were further validated by independent immunofluorescence and 3D colocalization assays. Functional differences in lipid droplet turnover and lipophagy were confirmed using complementary approaches, including pulse-chase assays, lipolysis inhibition, LipiDye™-M imaging, autophagy marker colocalization, and starvation-induced autophagy assays. All replication attempts described in the study were successful, with no major findings reported as non-reproducible.
Randomization	We ensured that plating of cells were not standardized by genotypes to avoid plate effects on microscopy assays.
Blinding	Both proteomics and lipidomics analysis was performed using blinding of the end user. Samples were given a numeric code and were processed together. The analysis of data was performed by individuals who did not run the experiment.

Reporting for specific materials, systems and methods

We require information from authors about some types of materials, experimental systems and methods used in many studies. Here, indicate whether each material, system or method listed is relevant to your study. If you are not sure if a list item applies to your research, read the appropriate section before selecting a response.

Materials & experimental systems

n/a	Involved in the study
☐	☒ Antibodies
☐	☒ Eukaryotic cell lines
☐	☐ Palaeontology and archaeology
☐	☐ Animals and other organisms
☐	☐ Clinical data
☐	☐ Dual use research of concern
☐	☐ Plants

Methods

n/a	Involved in the study
☐	☐ ChIP-seq
☐	☐ Flow cytometry
☐	☐ MRI-based neuroimaging

Antibodies

Antibodies used

We have included a table in our supplementary information on all the antibodies used in the studies.

Validation

Primary antibodies

- Proteintech 15294-1-AP (PLIN2/ADRP) — KD/KO validated by vendor; validated for WB and ICC among their applications. https://www.ptglab.com/products/ADRP-Antibody-15294-1-AP.htm This antibody has been used in over 183 publications.
- Sigma/Merck G9545 (GAPDH, rabbit) — Affinity-purified against immunizing peptide; datasheet details immunogen and purification; commonly used for WB/IF with over 140 citations. Supported by vendor figures https://www.sigmaaldrich.com/US/en/product/sigma/g9545
- CST 12238 (Calreticulin, D3E6 XP) — Highly specific and rigorously validated in-house for WB, IF, flow; datasheet notes recognition of endogenous calreticulin and use on live cells under ICD conditions. 233 citations https://www.cellsignal.com/products/primary-antibodies/calreticulin-d3e6-xp-rabbit-mab/12238
- Proteintech 21327-1-AP (Cathepsin D) — KD/KO validated; validated for WB/IHC/IF with KO/sgRNA data shown by vendor. https://www.ptglab.com/products/CTSD-Antibody-21327-1-AP.htm Used in 134 publications.
- Abcam ab205236 (Mitofusin-2, EPR19796) — KO validated by Abcam. Recombinant rabbit mAb validated for WB among other applications; product page lists multiple publications and application images. https://www.abcam.com/en-us/products/primary-antibodies/mitofusin-2-antibody-epr19796-ab205236
- Abcam ab4674 (GFAP, chicken polyclonal) — Validated for WB, ICC and other applications; extensively cited (600+ publications on product page).
- Abcam ab9512 (Aquaporin-4, clone 4/18) — Validated for staining by vendor and in over 40 literature citations.
- Atlas Antibodies/Sigma HPA055173 (PNPLA2/ATGL) — Human Protein Atlas “Prestige” antibody; validated by Human Protein Atlas with extensive characterization; enhanced validation noted for ICC-IF.
- Proteintech CL488-16275 (DHRS1, CoraLite® Plus 488) — Validated for ICC/IF; vendor shows IF of A431 cells and provides “Selected Validation Data” on the datasheet. https://www.ptglab.com/products/DHRS1-Antibody-CL488-16275.htm
- CST 83319 (ACSL3, E2S9L) — CST has validated this antibody for IF among other applications in cells known to have high and low expression of ACSL3. https://www.cellsignal.com/products/primary-antibodies/acsl3-e2s9l-rabbit-mab/83319
- Thermo Fisher PA5-58931 (RUFY2) — Validated for ICC with images provided by manufacturer https://www.thermofisher.com/antibody/product/RUFY2-Antibody-Polyclonal/PA5-58931
- Thermo Fisher PA5-139939 (DHRSX) — Validated for ICC with images provided by manufacturer. https://www.thermofisher.com/antibody/product/DHRSX-Antibody-Polyclonal/PA5-139939
- Thermo Fisher PA5-88882 (HSD17B8) — Validated for WB and ICC/IF with vendor images https://www.thermofisher.com/antibody/product/HSD17B8-Antibody-Polyclonal/PA5-88882
- CST 7695 (SQSTM1/p62, D10E10) — Validated with chemical compounds known to disrupt localization for ICC use case. 65 citations. https://www.cellsignal.com/products/primary-antibodies/sqstm1-p62-d10e10-rabbit-mab/7695
- Abcam ab192890 (LC3B, EPR18709) — KO-validated recombinant mAb; validated for ICC among other applications; included in knockout-validated antibody listings. Cited in at least 468 publications. https://www.abcam.com/en-us/products/primary-antibodies/lc3b-antibody-epr18709-autophagosome-marker-ab192890
- Abcam ab52607 (APOE) --KO validated for confirmed specificity, Biophysical QC for unrivalled batch-batch consistency, cited in over 30 publications. https://www.abcam.com/en-us/products/primary-antibodies/apolipoprotein-e-antibody-ep1374y-ab52607
- Millipore Sigma AB947 (APOE) -- validated in this manuscript using an APOE knockout line.
- BD 610457 (EEA1 , clone 14) -- Validated by images provided from manufacturer. Cited in over 477 publications. https://www.bdbiosciences.com/en-at/products/reagents/microscopy-imaging-reagents/immunofluorescence-reagents/purified-mouse-anti-eea1.610457?tab=citations_references
- BD 610822 (GM 130, clone 35) -- Validated by images provided from manufacturer. Cited in over 1109 publications. https://www.bdbiosciences.com/en-us/products/reagents/microscopy-imaging-reagents/immunofluorescence-reagents/purified-mouse-anti-gm130.610822?tab=citations_references

Secondary antibodies (specificity/cross-adsorption validation)

- Thermo Fisher A-21206 (Donkey anti-Rabbit IgG (H+L), Alexa Fluor 488, HCA) — Highly cross-adsorbed to minimize species cross-reactivity; extensive published figures;
- Thermo Fisher A-11039 (Goat anti-Chicken IgY (H+L), Alexa Fluor 488) — Vendor page with testing data; designed for IgY detection with Alexa Fluor dye.
- Thermo Fisher A-21202 (Donkey anti-Mouse IgG (H+L), Alexa Fluor 488, HCA) — Highly cross-adsorbed; vendor and distributor pages note minimized cross-reactivity across many species; application images provided.

• ABclonal AS014 (HRP Goat anti-Rabbit IgG (H+L)) — Validated in WB/IHC/ELISA; datasheet shows WB and IHC images and recommended dilutions.
• Abcam AB97110 (HRP Donkey anti-Goat IgG (H+L)) -- Validated in WB/IHC/ELISA; website shows WB and IHC images and recommended dilutions.

Eukaryotic cell lines

Policy information about cell lines and Sex and Gender in Research

Cell line source(s)	Our human iPSC-derived astrocytes were derived from two different human iPSC isogenic sets-- BIONI037 (EBISC, FEMALE) and KOLF2.1J (JAX,MALE). BIONI037 was derived from a female donor while KOLF2.1J was derived from a male donor. We ensured that all data presented was consistent across both isogenic sets of lines, each which contained lines of 3 common APOE genotypes.
Authentication	All lines were tested to ensure that they were free from mycoplasma and were karyotypically normal.
Mycoplasma contamination	All lines were tested to ensure that they were free from mycoplasma using a PCR-based kit (ABI biosciences).
Commonly misidentified lines (See ICLAC register)	*Name any commonly misidentified cell lines used in the study and provide a rationale for their use.*

Palaeontology and Archaeology

Specimen provenance	*Provide provenance information for specimens and describe permits that were obtained for the work (including the name of the issuing authority, the date of issue, and any identifying information). Permits should encompass collection and, where applicable, export.*
Specimen deposition	*Indicate where the specimens have been deposited to permit free access by other researchers.*
Dating methods	*If new dates are provided, describe how they were obtained (e.g. collection, storage, sample pretreatment and measurement), where they were obtained (i.e. lab name), the calibration program and the protocol for quality assurance OR state that no new dates are provided.*

☐ Tick this box to confirm that the raw and calibrated dates are available in the paper or in Supplementary Information.

Ethics oversight	*Identify the organization(s) that approved or provided guidance on the study protocol, OR state that no ethical approval or guidance was required and explain why not.*

Note that full information on the approval of the study protocol must also be provided in the manuscript.

Animals and other research organisms

Policy information about studies involving animals; ARRIVE guidelines recommended for reporting animal research, and Sex and Gender in Research

Laboratory animals	*For laboratory animals, report species, strain and age OR state that the study did not involve laboratory animals.*
Wild animals	*Provide details on animals observed in or captured in the field; report species and age where possible. Describe how animals were caught and transported and what happened to captive animals after the study (if killed, explain why and describe method; if released, say where and when) OR state that the study did not involve wild animals.*
Reporting on sex	*Indicate if findings apply to only one sex; describe whether sex was considered in study design, methods used for assigning sex. Provide data disaggregated for sex where this information has been collected in the source data as appropriate; provide overall numbers in this Reporting Summary. Please state if this information has not been collected. Report sex-based analyses where performed, justify reasons for lack of sex-based analysis.*
Field-collected samples	*For laboratory work with field-collected samples, describe all relevant parameters such as housing, maintenance, temperature, photoperiod and end-of-experiment protocol OR state that the study did not involve samples collected from the field.*
Ethics oversight	*Identify the organization(s) that approved or provided guidance on the study protocol, OR state that no ethical approval or guidance was required and explain why not.*

Note that full information on the approval of the study protocol must also be provided in the manuscript.

Clinical data

Policy information about [clinical studies]
All manuscripts should comply with the ICMJE [guidelines for publication of clinical research] and a completed [CONSORT checklist] must be included with all submissions.

Clinical trial registration — *Provide the trial registration number from ClinicalTrials.gov or an equivalent agency.*

Study protocol — *Note where the full trial protocol can be accessed OR if not available, explain why.*

Data collection — *Describe the settings and locales of data collection, noting the time periods of recruitment and data collection.*

Outcomes — *Describe how you pre-defined primary and secondary outcome measures and how you assessed these measures.*

Dual use research of concern

Policy information about [dual use research of concern]

Hazards

Could the accidental, deliberate or reckless misuse of agents or technologies generated in the work, or the application of information presented in the manuscript, pose a threat to:

No	Yes	
☒	☐	Public health
☒	☐	National security
☒	☐	Crops and/or livestock
☒	☐	Ecosystems
☒	☐	Any other significant area

Experiments of concern

Does the work involve any of these experiments of concern:

No	Yes	
☒	☐	Demonstrate how to render a vaccine ineffective
☒	☐	Confer resistance to therapeutically useful antibiotics or antiviral agents
☒	☐	Enhance the virulence of a pathogen or render a nonpathogen virulent
☒	☐	Increase transmissibility of a pathogen
☒	☐	Alter the host range of a pathogen
☒	☐	Enable evasion of diagnostic/detection modalities
☒	☐	Enable the weaponization of a biological agent or toxin
☒	☐	Any other potentially harmful combination of experiments and agents

Plants

Seed stocks — *Report on the source of all seed stocks or other plant material used. If applicable, state the seed stock centre and catalogue number. If plant specimens were collected from the field, describe the collection location, date and sampling procedures.*

Novel plant genotypes — *Describe the methods by which all novel plant genotypes were produced. This includes those generated by transgenic approaches, gene editing, chemical/radiation-based mutagenesis and hybridization. For transgenic lines, describe the transformation method, the number of independent lines analyzed and the generation upon which experiments were performed. For gene-edited lines, describe the editor used, the endogenous sequence targeted for editing, the targeting guide RNA sequence (if applicable) and how the editor was applied.*

Authentication — *Describe any authentication procedures for each seed stock used or novel genotype generated. Describe any experiments used to assess the effect of a mutation and, where applicable, how potential secondary effects (e.g. second site T-DNA insertions, mosiacism, off-target gene editing) were examined.*

ChIP-seq

Data deposition

☐ Confirm that both raw and final processed data have been deposited in a public database such as GEO.

☐ Confirm that you have deposited or provided access to graph files (e.g. BED files) for the called peaks.

Data access links<br>*May remain private before publication.*	*For "Initial submission" or "Revised version" documents, provide reviewer access links. For your "Final submission" document, provide a link to the deposited data.*
Files in database submission	*Provide a list of all files available in the database submission.*
Genome browser session<br>(e.g. UCSC)	*Provide a link to an anonymized genome browser session for "Initial submission" and "Revised version" documents only, to enable peer review. Write "no longer applicable" for "Final submission" documents.*

Methodology

Replicates	*Describe the experimental replicates, specifying number, type and replicate agreement.*
Sequencing depth	*Describe the sequencing depth for each experiment, providing the total number of reads, uniquely mapped reads, length of reads and whether they were paired- or single-end.*
Antibodies	*Describe the antibodies used for the ChIP-seq experiments; as applicable, provide supplier name, catalog number, clone name, and lot number.*
Peak calling parameters	*Specify the command line program and parameters used for read mapping and peak calling, including the ChIP, control and index files used.*
Data quality	*Describe the methods used to ensure data quality in full detail, including how many peaks are at FDR 5% and above 5-fold enrichment.*
Software	*Describe the software used to collect and analyze the ChIP-seq data. For custom code that has been deposited into a community repository, provide accession details.*

Flow Cytometry

Plots

Confirm that:

☐ The axis labels state the marker and fluorochrome used (e.g. CD4-FITC).

☐ The axis scales are clearly visible. Include numbers along axes only for bottom left plot of group (a 'group' is an analysis of identical markers).

☐ All plots are contour plots with outliers or pseudocolor plots.

☐ A numerical value for number of cells or percentage (with statistics) is provided.

Methodology

Sample preparation	*Describe the sample preparation, detailing the biological source of the cells and any tissue processing steps used.*
Instrument	*Identify the instrument used for data collection, specifying make and model number.*
Software	*Describe the software used to collect and analyze the flow cytometry data. For custom code that has been deposited into a community repository, provide accession details.*
Cell population abundance	*Describe the abundance of the relevant cell populations within post-sort fractions, providing details on the purity of the samples and how it was determined.*
Gating strategy	*Describe the gating strategy used for all relevant experiments, specifying the preliminary FSC/SSC gates of the starting cell population, indicating where boundaries between "positive" and "negative" staining cell populations are defined.*

☐ Tick this box to confirm that a figure exemplifying the gating strategy is provided in the Supplementary Information.

Magnetic resonance imaging

Experimental design

Design type	*Indicate task or resting state; event-related or block design.*
Design specifications	*Specify the number of blocks, trials or experimental units per session and/or subject, and specify the length of each trial or block (if trials are blocked) and interval between trials.*

Behavioral performance measures

State number and/or type of variables recorded (e.g. correct button press, response time) and what statistics were used to establish that the subjects were performing the task as expected (e.g. mean, range, and/or standard deviation across subjects).

Acquisition

Imaging type(s)

Specify: functional, structural, diffusion, perfusion.

Field strength

Specify in Tesla

Sequence & imaging parameters

Specify the pulse sequence type (gradient echo, spin echo, etc.), imaging type (EPI, spiral, etc.), field of view, matrix size, slice thickness, orientation and TE/TR/flip angle.

Area of acquisition

State whether a whole brain scan was used OR define the area of acquisition, describing how the region was determined.

Diffusion MRI ☐ Used ☐ Not used

Preprocessing

Preprocessing software

Provide detail on software version and revision number and on specific parameters (model/functions, brain extraction, segmentation, smoothing kernel size, etc.).

Normalization

If data were normalized/standardized, describe the approach(es): specify linear or non-linear and define image types used for transformation OR indicate that data were not normalized and explain rationale for lack of normalization.

Normalization template

Describe the template used for normalization/transformation, specifying subject space or group standardized space (e.g. original Talairach, MNI305, ICBM152) OR indicate that the data were not normalized.

Noise and artifact removal

Describe your procedure(s) for artifact and structured noise removal, specifying motion parameters, tissue signals and physiological signals (heart rate, respiration).

Volume censoring

Define your software and/or method and criteria for volume censoring, and state the extent of such censoring.

Statistical modeling & inference

Model type and settings

Specify type (mass univariate, multivariate, RSA, predictive, etc.) and describe essential details of the model at the first and second levels (e.g. fixed, random or mixed effects; drift or auto-correlation).

Effect(s) tested

Define precise effect in terms of the task or stimulus conditions instead of psychological concepts and indicate whether ANOVA or factorial designs were used.

Specify type of analysis: ☐ Whole brain ☐ ROI-based ☐ Both

Statistic type for inference

(See Eklund et al. 2016)

Specify voxel-wise or cluster-wise and report all relevant parameters for cluster-wise methods.

Correction

Describe the type of correction and how it is obtained for multiple comparisons (e.g. FWE, FDR, permutation or Monte Carlo).

Models & analysis

n/a	Involved in the study
☐	☐ Functional and/or effective connectivity
☐	☐ Graph analysis
☐	☐ Multivariate modeling or predictive analysis

Functional and/or effective connectivity

Report the measures of dependence used and the model details (e.g. Pearson correlation, partial correlation, mutual information).

Graph analysis

Report the dependent variable and connectivity measure, specifying weighted graph or binarized graph, subject- or group-level, and the global and/or node summaries used (e.g. clustering coefficient, efficiency, etc.).

Multivariate modeling and predictive analysis

Specify independent variables, features extraction and dimension reduction, model, training and evaluation metrics.
