# Supplementary material for: APOE genotypes differentially remodel the astrocytic lipid droplet proteome to shape lipid droplet dynamics
Source: Nat Commun. 2026 Aug 19;17:9900. doi: 10.1038/s41467-026-76565-6 (PMC13582944; doi:10.1038/s41467-026-76565-6)
Supplement: Supplementary file 4 [file 41467_2026_76565_MOESM4_ESM.pdf]

Description of Additional Supplementary Files

Supplementary Data 1-14

Description: Proteomics source data.

Supplementary Data 15-26

Description: Lipidomics source data.

Supplementary Data 27

Description: Antibodies used in this study.
